# Leucine-rich alpha-2-glycoprotein 1 (LRG1) as a novel ADC target†

**DOI:** 10.1039/d1cb00104c

**Published:** 2021-05-31

**Authors:** Faiza Javaid, Camilla Pilotti, Carlotta Camilli, David Kallenberg, Calise Bahou, Jack Blackburn, James R. Baker, John Greenwood, Stephen E. Moss, Vijay Chudasama

**Affiliations:** UCL Department of Chemistry 20 Gordon Street London WC1H 0AJ UK v.chudasama@ucl.ac.uk; UCL Institute of Ophthalmology 11-43 Bath Street London EC1V 9EL UK j.greenwood@ucl.ac.uk s.moss@ucl.ac.uk

## Abstract

Leucine-rich alpha-2-glycoprotein 1 (LRG1) is present abundantly in the microenvironment of many tumours where it contributes to vascular dysfunction, which impedes the delivery of therapeutics. In this work we demonstrate that LRG1 is predominantly a non-internalising protein. We report the development of a novel antibody–drug conjugate (ADC) comprising the anti-LRG1 hinge-stabilised IgG4 monoclonal antibody Magacizumab coupled to the anti-mitotic payload monomethyl auristatin E (MMAE) *via* a cleavable dipeptide linker using the site-selective disulfide rebridging dibromopyridazinedione (diBrPD) scaffold. It is demonstrated that this ADC retains binding post-modification, is stable in serum and effective in *in vitro* cell studies. We show that the extracellular LRG1-targeting ADC provides an increase in survival *in vivo* when compared against antibody alone and similar anti-tumour activity when compared against standard chemotherapy, but without undesired side-effects. LRG1 targeting through this ADC presents a novel and effective proof-of-concept *en route* to improving the efficacy of cancer therapeutics.

## Abstract 2

LRG1 is present abundantly in the microenvironment of many tumours. LRG1 targeting through the reported non-internalising ADC presents a novel and effective proof-of-concept *en route* to improving the efficacy of cancer therapeutics.

## Graphical abstract

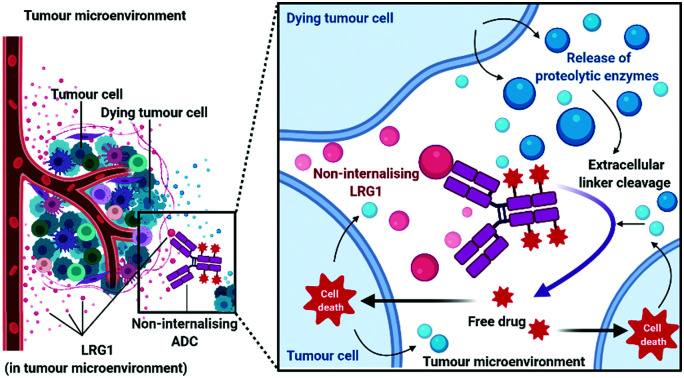

## Introduction

An antibody–drug conjugate (ADC) comprises an antibody that recognises tumour specific antigens to which a highly potent cytotoxic agent is conjugated *via* a chemical linkage.^1^ ADCs couple the targeting selectivity of an antibody moiety with the cell killing capacity of a cytotoxic payload. To date, nine ADCs have been approved by the FDA^2–11^ and there are currently over 80 ADCs undergoing evaluation in clinical trials worldwide.^12^

Traditionally, upon binding to its target antigen an ADC–antigen complex internalises *via* receptor-mediated endocytosis to enable intracellular release of a cytotoxic payload.^2–11^ In recent years however, there has been a considerable shift from the belief that ADCs are strictly dependent upon selective binding and internalisation of the antibody into tumour cells to release their cytotoxic payload in order for them to be effective.^13^ For example, it has emerged that internalisation is not essential to cause tumour cell death.^14^ Through the use of extracellular cleavable linker-bearing ADCs, non-internalising ADCs have shown great promise.^14^ Indeed, it has been shown that targeting ADCs to components in the tumour extracellular space or to non-internalising tumour markers has considerable therapeutic activity.^15–18^ Evidence of potent preclinical activity in cancer models has been reported for non-internalising ADC products directed against a number of targets including fibrin^15^ and collagen IV,^16^ as well as splice variants of tenascin-C,^17,18^ which are all components of the tumour extracellular matrix.

Leucine-rich alpha-2 glycoprotein-1 (LRG1) is a secreted glycoprotein which is commonly induced in pathological lesions where, amongst other properties, it promotes dysfunctional vessel growth.^19,20^ LRG1 contributes to pathological angiogenesis by corrupting the homeostatic influence of TGFβ signalling,^20^ and promotes vessel dysfunction by interfering with vessel stabilisation and maturation.^21^ Increasingly, therefore, LRG1 is seen as an important factor in determining vessel abnormality in a wide range of diseases, including cancer. Accumulating evidence suggests that LRG1 is involved in the growth and progression of a variety of cancer types as significantly elevated expression of LRG1 in serum and solid tumours has been found to be associated with a poor prognosis.^22–27^

Vessel normalisation approaches, that promote the growth of functional vessels by enhancing oxygen and nutrient delivery to the vessels, have gained much attention in recent years as a means to improve the outcome of anti-cancer drugs.^28–30^ As LRG1 is dispensable for developmental angiogenesis,^20^ attempts to neutralise the pro-angiogenic and vasculopathic activity of LRG1 have been investigated and have led to the development of a function-blocking fully humanised IgG4 antibody against LRG1.^31^ The Moss and Greenwood groups have shown that inhibiting LRG1 reverses its detrimental effects on the vasculature and leads to partial restoration of normal vascular function^21,31^ and consequently improvement in the delivery of cytotoxic and immune co-therapies.^21^ These observations suggest that LRG1 is a promising therapeutic target in pathological angiogenesis, particularly as blockade of this protein targets an orthogonal pathway to VEGF and, in the context of TGFβ, inhibits the activator of the pathogenic signalling arm without interfering with homeostatic activities. The expression of LRG1 in many cancers and the presence of LRG1 at high concentrations in the tumour microenvironment makes it a promising target.

In this study, we attempt to evaluate whether LRG1 is a suitable target for an ADC based cancer therapy. We demonstrate in an *in vitro* cell assay that, upon secretion, LRG1 does not associate with the cell membrane or become internalised. We report the development of a novel non-internalising ADC comprising an anti-LRG1 hinge-stabilised IgG4 monoclonal antibody named Magacizumab^31^ coupled to the anti-mitotic payload monomethyl auristatin E (MMAE) *via* a cleavable dipeptide linker, using the site-selective disulfide rebridging dibromopyridazinedione (diBrPD) scaffold.^32^ It is well understood that in order for ADCs to deliver their full potential, sophisticated conjugation strategies to connect the drug to the linker are required.^33–35^ We chose to apply pyridazinediones (PDs) that can functionally rebridge cysteine residues liberated upon reduction of interchain disulfide bonds as the antibody conjugation method. This method was selected in view of their favourable properties in terms of reproducibility, homogeneity, serum stability and exemplification *in vitro* and *in vivo*.^32,36–39^ We demonstrate that this ADC retains binding post-modification, is stable in serum and effective in *in vitro* cell studies. We developed a human LRG1 (hLRG1)-expressing B16F0 mouse melanoma cell line and demonstrate that Magacizumab binds to LRG1 and localises to the tumour site. We also report that the novel LRG1-targeting ADC liberates its cytotoxic payload, which is coupled to the antibody *via* a cleavable dipeptide linker, presumably upon being metabolised by appropriate proteases (*e.g.* cathepsin B) and increases survival when applied to a subcutaneous mouse melanoma tumour model. Targeting of LRG1 through this reported ADC presents a novel and effective proof-of-concept *en route* to improving the efficacy of cancer therapeutics.

## Results and discussion

### Choice of antibody

Magacizumab,^31^ an IgG4 monoclonal antibody against human LRG1 (hLRG1) was selected as the tumour-targeting moiety for the development of the ADC. Magacizumab incorporates a hinge-stabilising S228P mutation, which is commonly introduced into an IgG4 to overcome this isotype associated issue of Fab arm exchange and hemibody formation.^40–42^ Many ADCs including Mylotarg^2,3^ and Besponsa^43^ that employ IgG4s have this modification to the hinge region. Magacizumab has also successfully shown the inhibition of vascular leakage in mouse models of pathological angiogenesis.^31^

### Choice of linker and fluorophore

**Fig. 1:**
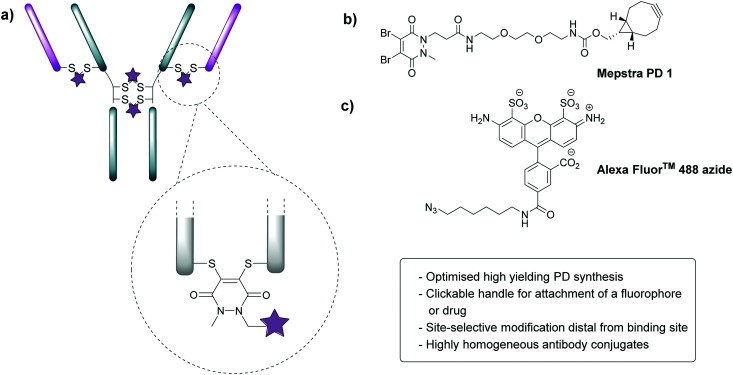
Properties of dibromopyridazinediones (diBrPDs) and the structure of PD **1**. (a) diBrPDs can be used to functionally rebridge antibodies. Incorporation of a clickable handle in their design enables attachment of a fluorophore or drug, represented by a star; (b) chemical structure of PD **1** with a strained alkyne handle; (c) chemical structure of Alexa Fluor^TM^ 488 (fluorophore) bearing an azide handle.

In order to appraise the internalisation potential of Magacizumab into cancer cells, it needed to be modified with a suitable fluorophore. To achieve this, we employed the thiol-selective dibromopyridazinedione (diBrPD) scaffold to site-selectively modify the solvent accessible disulfide bonds on Magacizumab.^36^ In addition to producing conjugates with generally higher levels of homogeneity, antibody-PD conjugates provide serum stable bioconjugates and a versatile platform for the functionalisation of antibodies.^38^ In view of this, we chose to employ PD-strained alkyne **1** as our choice of linker in this study.^32^ The incorporation of a ‘clickable’ stained alkyne handle in the design of our PD linker (Fig. 1) enables the covalent and controlled attachment of an azide-bearing fluorophore (or a drug for later studies) by strain-promoted azide–alkyne cycloaddition (SPAAC), allowing chemical conjugation under aqueous conditions without the need for toxic catalysts. By using this technology, conjugation efficiency of the antibody functional rebridging by PD **1** could be appraised by SDS-PAGE and UV-Vis spectroscopy, taking advantage of the characteristic absorbance of PDs at 335 nm. We also needed to select an appropriate fluorophore to ‘click’ to the antibody-PD conjugate. To this end, the photostable, water-soluble Alexa Fluor^TM^ 488 azide (to facilitate ‘click’ reaction with the strained alkyne on the PD moiety) with a maximum absorbance at 490 nm was selected. Reaction with this fluorophore would enable us to confirm if the PD loading obtained by UV-Vis is a good measure of loading on an antibody as the ‘click’ should proceed completely, *i.e.* can PD loading obtained by UV-Vis be used to ascertain drug loading onto the antibody.

### Appraisal of PD chemistry on Magacizumab

In view of the above, our study began with the synthesis of PD-strained alkyne **1** (Fig. 1b, see ESI,† for synthesis details) using an optimised procedure reported by Bahou *et al.*^32^ We next focused on optimising the conjugation of PD **1** to Magacizumab. Complete reduction of Magacizumab's interchain disulfide bonds was achieved with 100 molar equivalents (eq.) of the reducing agent tris(2-carboxyethyl)phosphine (TCEP) (see ESI,† Fig. S6). Whilst *in situ* protocols (reduction and rebridging agents added at once) are usually employed for PD modification to obtain near homogeneous conjugates, *in lieu* of the large excess of TCEP required for the complete reduction of Magacizumab, we favoured a step-wise conjugation protocol where reduction is performed first, followed by a wash step to remove unreacted reducing reagent prior to adding the PD **1** to enable rebridging.

**Fig. 2:**
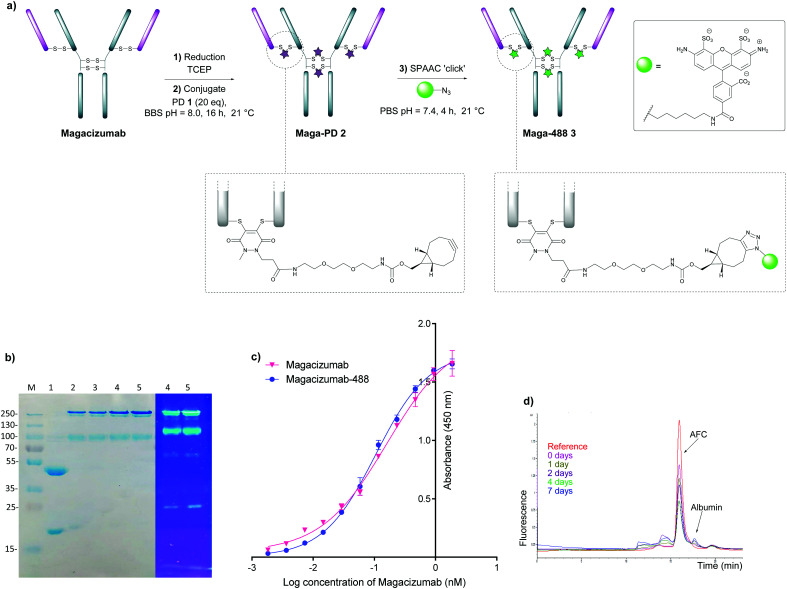
Step-wise modification of Magacizumab by PD 1 to form the antibody-fluorophore conjugate (AFC) **3**. (a) Conditions for generation of Magacizumab-Alexa Fluor^TM^ 488 conjugate **3** (Maga-488 **3**); (b) SDS-PAGE analysis. M – marker, Lane 1 – reduced Magacizumab, Lane 2 – Rebridged Magacizumab **2a** at 21 °C, Lane 3 – Rebridged Magacizumab **2b** at 4 °C, Lane 4 – AFC **3a** at 21 °C, Lane 5 – AFC **3b** at 4 °C; (c) ELISA showing binding affinity of AFC **3a** and Magacizumab to hLRG1; (d) serum stability analysis of AFC **3a** by HPLC-SEC.

To this end, reduction of Magacizumab (20 μM, pH 8.0 in borate buffer (BBS) with EDTA) was achieved by incubation of the antibody with TCEP (20 mM final concentration, 100 eq.) for 4 h at 37 °C. The excess reducing agent was removed by ultrafiltration into EDTA-containing BBS (pH 8.0) and this was followed by addition of the bridging reagent, PD **1** (20 mM final concentration, 20 eq.). An average PD-to-antibody ratio (PAR) of *ca.* 4 (determined by UV-Vis analysis) was obtained for Magacizumab-PD **2**, confirming that all solvent accessible interchain disulfide bonds had been modified. Whilst, SDS-PAGE analysis revealed a small proportion of the hinge region disulfide bonds had been rebridged in a non-native conformation (resulting in the presence of ‘half antibody’ isomer)^44–47^ (Fig. 2b) reports by *Bahou et al.* have demonstrated that the presence of this species does not affect antigen binding.^44^

Following this, the site-selectively modified full antibody bearing *ca.* 4 strained alkynes was reacted with a fluorophore-azide (Alexa Fluor^TM^ 488 azide) using strain-promoted azide–alkyne cycloaddition (SPAAC) to obtain antibody-fluorophore conjugate (AFC) **3**. Confirmation of a fluorophore loading (fluorophore-to-antibody ratio; FAR) of *ca.* 4 was obtained by UV-Vis spectroscopy, demonstrating that PD loading obtained by UV-Vis was a good measure of loading onto Magacizumab (see ESI,† for details). The effect of temperature on the bridging efficiency was also examined. Incubation over 16 hours at 21 °C and at 4 °C displayed no significant temperature dependence on the overall composition of the final construct AFC **3a** and **3b** respectively, generating ∼31% and ∼26% (calculated using ImageJ software, see Fig. S11, ESI†) ‘half-antibody’ product respectively. Conjugate **3a** was shown to have comparable binding activity by enzyme-linked immunosorbent assay (ELISA) to native Magacizumab (Fig. 2c). The stability of AFC **3a** was also examined in blood serum by incubation over a 7 day period and demonstrated excellent stability in serum for 7 days, as evidenced by minimal transfer of fluorescence to serum albumin over the period of incubation (Fig. 2d). The reduction in relative intensity observed for the AFC could be attributed to a minor amount of aggregation of the conjugate (Fig. 2d). These results provided proof-of-concept for the ability to site-selectively modify an IgG4-based antibody in a controlled manner using the PD scaffold.

### Analysis of Magacizumab cell internalisation

**Fig. 3:**
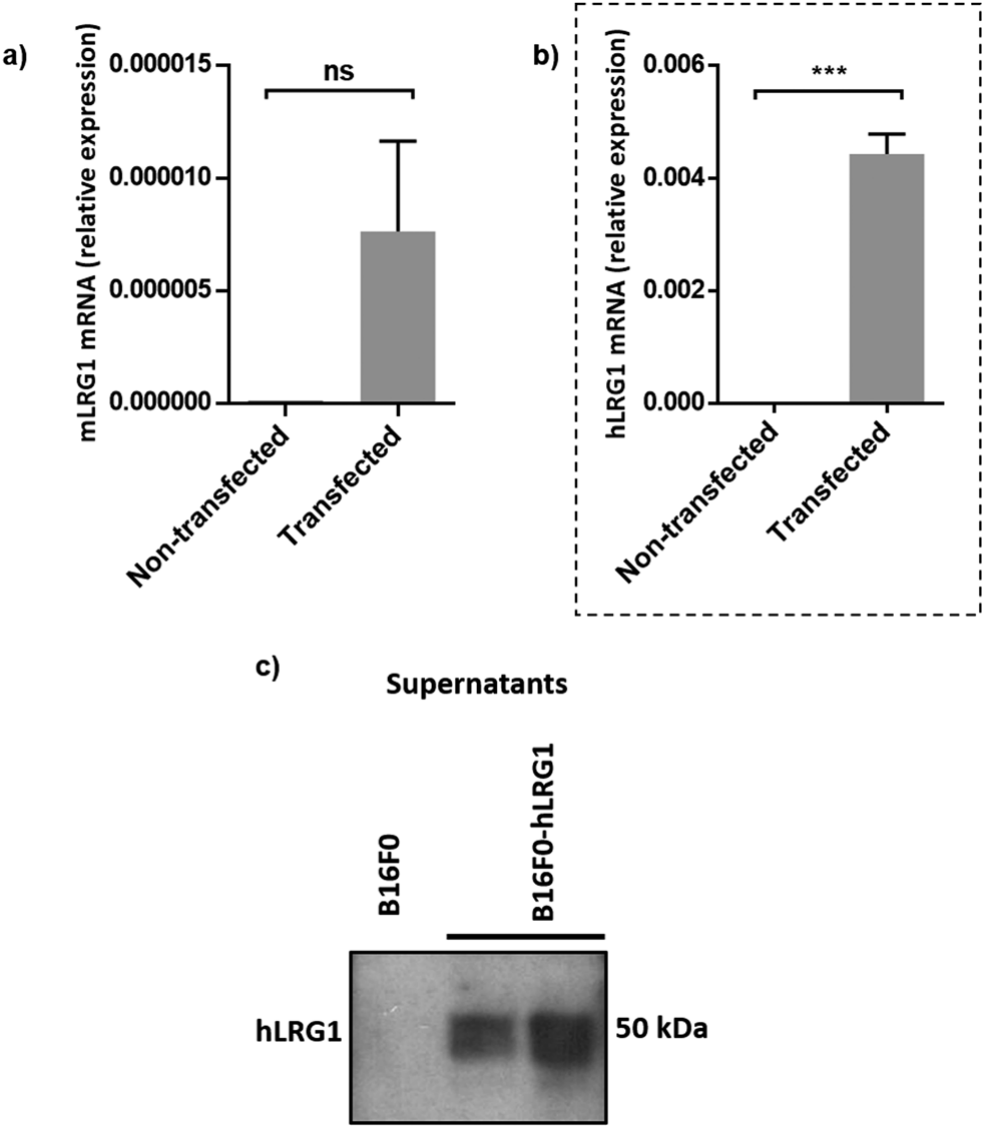
Generation of a stable B16F0 cell line expressing hLRG1. (a and b) qPCR was used to determine expression levels of mouse Lrg1 (a) and human Lrg1 (b) in transfected and non-transfected cells. Expression values were normalised to expression of the housekeeping gene mouse gapdh. Data are shown as mean ± SEM. Experiments were performed in triplicate (*n* = 3) and statistical analysis performed using Student's *t*-test (ns = not significant, *** = *P* ≤ 0.001). (c) western blot analysis of hLRG1 expression in B16F0 cells. Supernatants were probed using a rabbit anti-hLRG1 antibody.

Next, we investigated whether Magacizumab and/or hLRG1 (or a complex thereof) are internalised, as this was an important consideration for designing an appropriate drug linker for the efficient release of the payload at the target site. As it was not known whether secreted hLRG1 alone is internalised following adherence to the tumour cell surface, *via* its association with TGFβ family receptors or whether bound Magacizumab could stimulate internalisation, an *in vitro* assay was employed. First, we selected the B16F0 mouse melanoma cell line as this has previously been used to investigate the effect of LRG1 on vessel destabilisation.^21^ As the B16F0 parent cells were *Lrg1* null these were stably transfected with the human LRG1 protein. Success of stable transfection was confirmed by qPCR (Fig. 3a and b) and western blot (Fig. 3c). The development of this hLRG1-expressing cell line could also be beneficial to researchers interested in exploring the role of LRG1 in disease.

**Fig. 4:**
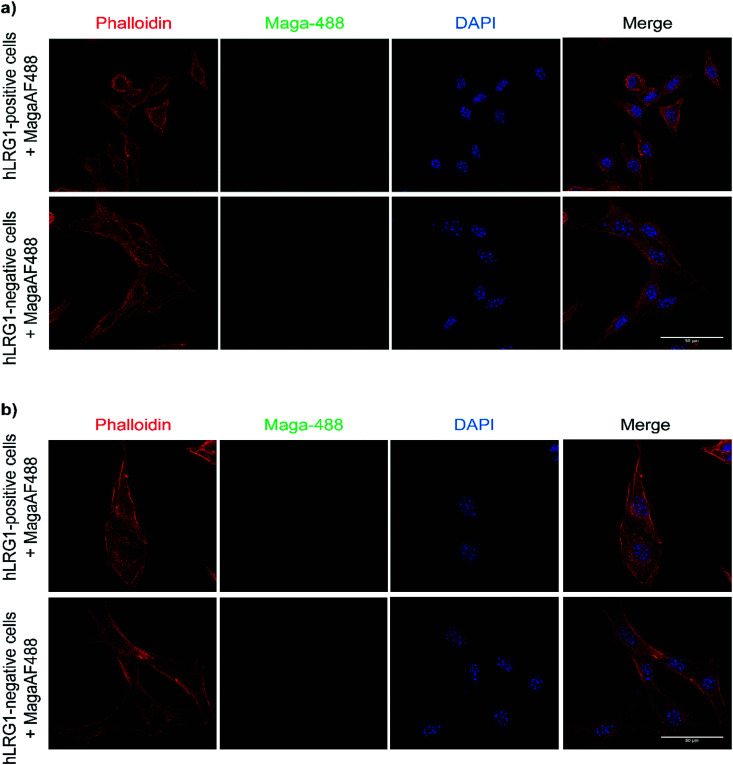
Analysis of Maga-488 (conjugate **3a**) binding and internalisation. (a) hLRG1-positive and hLRG1-negative B16F0 cells incubated with Maga-488 **3a** at 4 °C; (b) hLRG1-positive and hLRG1-negative cells incubated with Maga-488 **3a** at 37 °C. Phalloidin was used to stain actin and DAPI was used to stain nuclei. Scale bar, 50 μm.

Maga-488 **3a** was then incubated with hLRG1-positive B16F0 mouse melanoma cells and the parent non-transfected B16F0 cells as a control. Incubation of conjugate **3a** with cells was performed at 4 °C, to allow for antibody-LRG1-cell binding but not internalisation, and at 37 °C to permit internalisation of the construct. However, analysis by confocal microscopy revealed that under these *in vitro* conditions no signal was detectable for Maga-488 **3a**, either attached to the cell surface or internalised into the cells (Fig. 4). These findings indicate that Maga-LRG1 complex remains soluble (and therefore non-cell bound) and is washed out during the immunocytochemistry processing, resulting in no signal being observed.

**Fig. 5:**
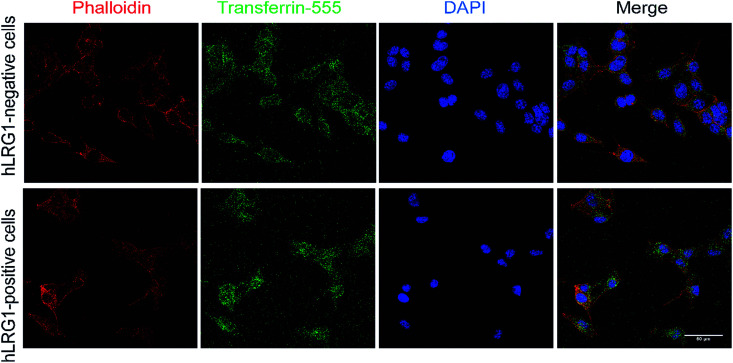
Internalisation analysis of transferrin-555 by confocal microscopy (positive control). Incubation of transferrin-555 with hLRG1-positive and hLRG1-negative B16F0 cells at 37 °C. Phalloidin was used to stain actin and DAPI was used to stain nuclei. Scale bar, 50 μm.

Similar methodology was performed using the transferrin receptor ligand as a positive control. Accordingly, B16F0 cells were incubated with labelled transferrin (transferrin-555) at 37 °C, which resulted in cytoplasmic localisation of the labelled transferrin (Fig. 5).

These findings indicated that the methodology used to investigate the internalisation of Magacizumab was indeed appropriate and confirmed that Magacizumab does not apparently bind and subsequently internalise into B16F0 cells that secrete hLRG1. These data suggest that Magacizumab binds to hLRG1 and may prevent it from binding to its molecular partners^20^ and, perhaps also entering the cell. In order to determine whether LRG1 internalisation is blocked by Magacizumab, or indeed if LRG1 is a non-internalising or cell surface binding protein, hLRG1 was labelled with Alexa Fluor^TM^ 555 (see ESI,† for details) and the internalisation assay performed again. hLRG1-positive B16F0 cells were incubated with labelled hLRG1 (hLRG1-555) alone or in the presence of Maga-488 **3a**. Confocal analysis revealed that despite the absence of Maga-488 **3a**, no signal for hLRG1 could be observed (see Fig. S22, ESI†), indicating that under these conditions hLRG1 is a non-internalising/cell surface binding, or at the very least is a poorly internalising/cell surface binding antigen. These observations suggest that under these conditions the hLRG1 detected earlier in western blotting (Fig. 3) remains free in the supernatant, and is not located at the cell surface.

Having established that secreted LRG1 is predominantly extracellular, and that consequently LRG1 bound Magacizumab will most likely remain in the extracellular space, an appropriate linker strategy was then required. Even in the event of other cancer cell types being able to internalise LRG1, the proportion available in the extracellular space for an anti-LRG1 ADC to target will far outweigh that available internally.

### Development of the anti-LRG1 ADC

Having demonstrated the inability of Magacizumab to internalise into B16F0 cells, it was rationalised that a non-internalising ADC bearing an extracellular cleavable linker could display anti-cancer activity by liberating its payload in the tumour extracellular space upon binding to hLRG1 in this region.^48^ MMAE has been successfully employed in many of the ADCs on the market and in late stage clinical trials, its cell permeability is essential in the design of a non-internalising ADC and it also possesses the favourable property of mediating killing of surrounding cells *via* the bystander effect.^14^ It was thus chosen as the cytotoxic payload for the anti-hLRG1 non-internalising ADC.

**Fig. 6:**
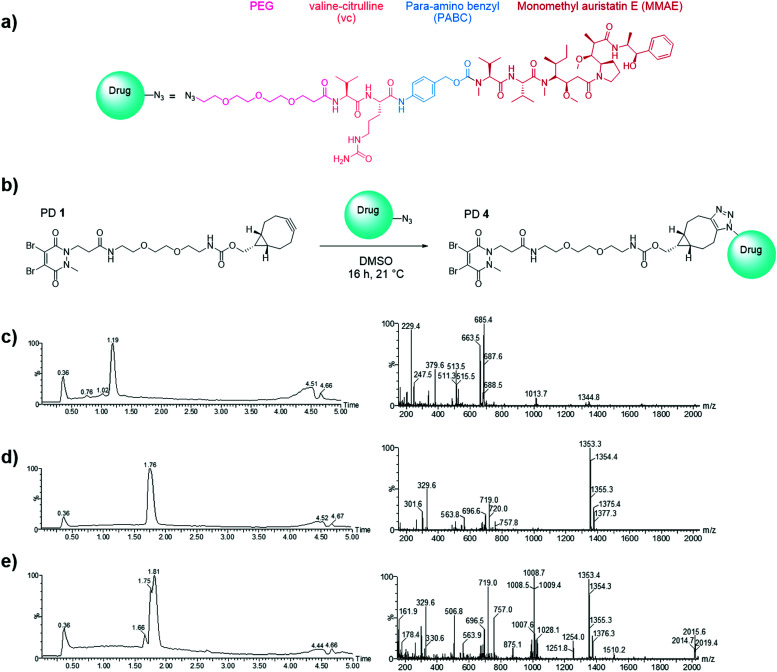
Selection of drug for the generation of the hLRG1-targeting ADC. (a) Structure of N_3_-PEG_3_-vc-PABC-MMAE; (b) SPAAC ‘click’ functionalisation (the pre-click strategy) reaction between the strained alkyne bearing PD **1** and azide-linked MMAE to form PD **4**. (c) LCMS analysis of PD **1** (662 Da); (d) N_3_-PEG_3_-vc-PABC-MMAE (1353 Da) and (e) pre-click reaction of PD **1** with N_3_-PEG_3_-vc-PABC-MMAE showing formation of PD **4** (2015 Da) and complete consumption of PD **1**.

Having already developed a strained alkyne PD that successfully site-selectively modified Magacizumab, we chose a linker-payload combination consisting of the antimitotic payload MMAE with an azide handle adjacent to an enzymatically cleavable dipeptide valine-citrulline (vc) linker equipped with a self-immolative *para*-aminobenzylcarbonyl (PABC) spacer and a solubilising PEG_3_ moiety (N_3_-PEG_3_-vc-PABC-MMAE, Fig. 6a). The incorporation of the azide group in the payload moiety satisfied the requirement for the presence of a functional handle on the drug to enable facile ‘click’ conjugation to Magacizumab-PD **2** conjugate to form the desired ADC. The PABC spacer hydrolytically decomposes upon decarboxylation, spontaneously releasing the free drug MMAE.^48^ As with others, we have previously experienced problems with ‘click’ conjugation of a site-selectively modified antibody bearing strained alkyne ‘click’ handles with hydrophobic-azide molecules. The conjugation reaction to form the ADC was therefore made more efficient by performing a ‘pre-click’ reaction where the disulfide-reactive PD-strained alkyne **1** is SPAAC ‘clicked’ with the azide-linked MMAE prior to site-selective conjugation to Magacizumab resulting in more efficient use of MMAE. Previous work in the group has also shown that direct conjugation of a hydrophobic PD is feasible and reproducible.^38^ As with previously labelled Magacizumab conjugates, quantification of drug loading could be determined by UV-Vis analysis, by taking advantage of the characteristic absorbance of PDs at 335 nm. This approach is substantiated by the results obtained by ‘click’ functionalisation of Magacizumab-PD **2** conjugate with Alexa Fluor^TM^ 488 azide. Determination of linker loading using this method aligns with what we, and others, have previously reported in literature.^32,37^

In view of the above, we first appraised the reaction of PD **1** with MMAE-azide using a 1 : 1.1 molar ratio of PD **1**: MMAE-azide as we wanted complete consumption of PD **1** (Fig. 6). After 4 h, LCMS analysis revealed that all the PD had been consumed in the ‘click’ reaction, furnishing disulfide reactive PD-linked MMAE **4** and a small excess of (unreacted) MMAE-azide (Fig. 6c–e).

**Fig. 7:**
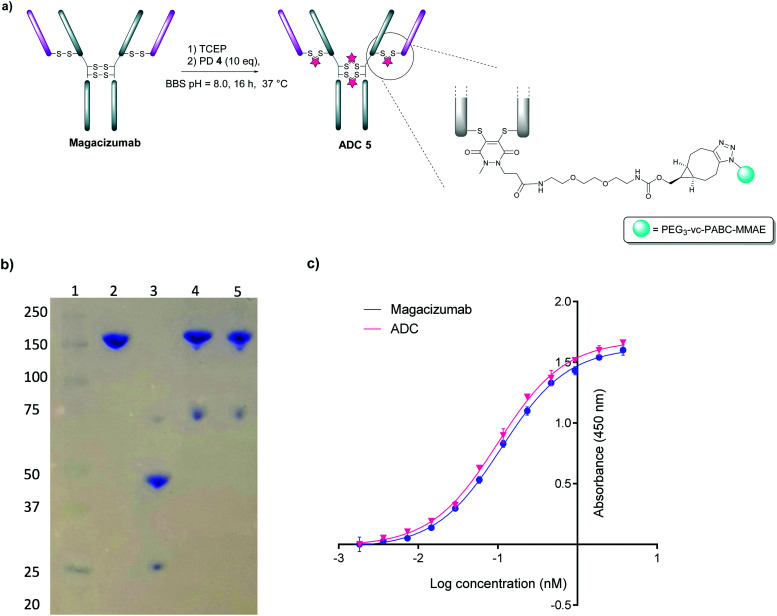
Construction of Magacizumab PD-MMAE ADC **5**, a hLRG1-targeting ADC. (a) Conditions for generation of Magacizumab PD-MMAE ADC **5**; (b) SDS-PAGE analysis of ADC products. Lane 1 – marker, Lane 2 – Native Magacizumab, Lane 3 – reduced Magacizumab, Lane 4 – Magacizumab PD-MMAE ADC **5**, Lane 5 – reduced Magacizumab PD-MMAE ADC **5** (reduction performed with 100 eq. TCEP at 37 °C; c) ELISA showing binding affinity of Magacizumab PD-MMAE ADC **5** and Magacizumab to LRG1.

We next appraised conjugation of PD-MMAE **4** with Magacizumab. In view of the need to scale up the ADC to evaluate efficacy, several distinct parameters were investigated for the formation of the ADC. The different reaction conditions that were tested for conjugation of pre-clicked PD **4** to Magacizumab to form the ADC included varying reaction temperature, equivalents of PD **4** used for rebridging and the final DMSO% of the conjugate. UV-Vis analysis was used to assess PAR (see Fig. S15, ESI†). It was found that all reaction conditions tested yielded ADCs with a PAR of ∼4 (see Fig. S15 and Table S3, ESI†). In the interests of scaling up the ADC reaction for cytotoxicity and therapy studies, the conditions that used the lowest equivalents of PD (10 eq.), had the lowest final DMSO% (6%) in the ADC and gave a high PAR were taken forward for large scale reactions (Fig. 7a). An Alexa Fluor^TM^ 488 azide ‘click’ control was also performed on this prepared ADC to demonstrate that all clickable handles on the functionally rebridged antibody had reacted with MMAE-azide and that no, or negligible, unreacted strained alkyne was present and to corroborate that the value for PD loading reflected drug loading. This gave a minimal fluorophore readout that was in line with control experiments, confirming that no/minimal strained alkyne clickable handles remained (see Fig. S16 and S17 for more details, ESI†).

The reaction to generate ADC **5** was successfully scaled up for *in vitro* and *in vivo* studies using the optimised conjugation conditions (Fig. 7a), with success of conjugation confirmed by UV-Vis and SDS-PAGE (Fig. 7b). As a control, we added TCEP to the ADC constructs (Fig. 7b, Lane 5) to assess whether the disulfide bonds are unmodified or if they are rebridged with PD-MMAE **4** (no reduction would occur), and we observed the latter result. Retention of binding affinity of ADC **5** to hLRG1 was also demonstrated by ELISA (Fig. 7c).

### *In vitro* cytotoxicity analysis

**Fig. 8:**
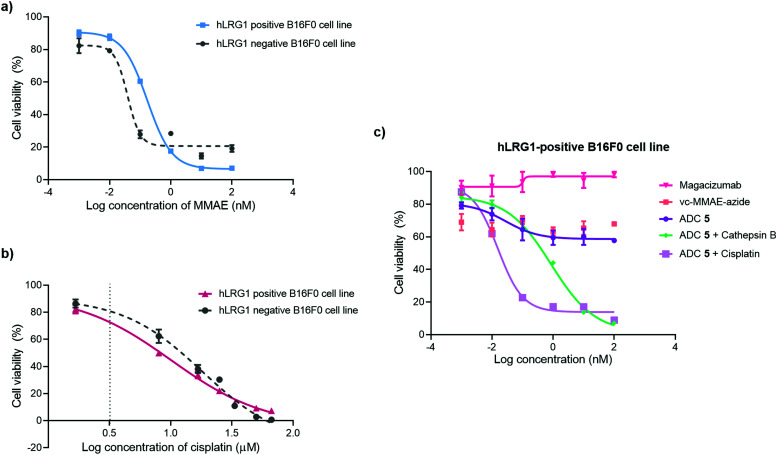
Inhibition of cell proliferation in cancer cell lines with different levels of hLRG1 expression. Cytotoxicity of (a) MMAE and (b) cisplatin in hLRG1-positive and hLRG1-negative B16F0 cells; (c) hLRG1-positive cells: ADC **5**, ADC **5** and cathepsin B, ADC **5** and cisplatin, vc-MMAE-azide in comparison with Magacizumab.

The *in vitro* cytotoxic potency of Magacizumab PD-MMAE ADC **5** was evaluated in hLRG1-positive and hLRG1-negative B16F0 cell lines. Initially, both cell lines were exposed to MMAE and exhibited a comparable reduction in cell viability at a similar concentration of the cytotoxic payload, with IC_50_ values of 0.2 nM and 0.1 nM for hLRG1 positive and negative B16F0 cell lines respectively (Fig. 8a). Next, the potency of PD-MMAE ADC **5** was evaluated against Magacizumab and vc-MMAE-N_3_ using the hLRG1-positive cell line. As predicted, Magacizumab alone had little influence on cell viability. Due to the non-internalising nature of the ADC, incubation of ADC **5** alone with cells did not impact cell viability, as release of payload from the ADC requires the presence of an adequate concentration of a specific protease in the extracellular environment (Fig. 8c). Similarly, vc-MMAE-N_3_ also had no cytotoxic effect on cells as the absence of dipeptide-cleaving protease prevents the release of free MMAE to exert its cytotoxic effect (Fig. 8c).

Following the above, we designed a novel assay for determining the potency of our non-internalising ADC. In order to mimic the tumour microenvironment and trigger the release of cytotoxic drug from the ADC, we prepared serial dilutions of ADC **5** in culture medium containing activated cathepsin B.^18^ This protease is highly abundant in the extracellular space of solid tumours and can cleave the dipeptide val-cit linkage promoting self-immolative release of free MMAE. As the pH of the tumour environment is often acidic,^49–53^ the pH of the culture medium was adjusted accordingly in all experimental groups (pH 6.5); this is particularly important for this experimental group as cathepsin B is preferentially active in an acidic environment.^54^ Gratifyingly, in the presence of exogenous cathepsin B, ADC **5** showed potency against the hLRG1-positive cell line.

We also investigated the effect of combination therapy on cell viability. This involved treating cells with a therapeutic combination comprising the hLRG1-targeting ADC **5** and a suboptimal amount of the chemotherapeutic agent cisplatin. The rationale of this combined therapy approach was to induce tumour cell death by the chemotherapeutic agent, resulting in increased amounts of cathepsin B being released from dead cells, which would amplify the liberation of free drug from ADC **5** and lead to an increased reduction in cell viability. First, the susceptibility of hLRG1-positive and hLRG1 negative B16F0 cell lines to cisplatin was determined (Fig. 8b). Both cell lines were sensitive to cisplatin over a 48 h period, showing a comparable reduction in cell viability, with IC_50_ values of 10 μM and 18 μM for hLRG1 positive and negative B16F0 cell lines respectively. To investigate how cells would respond to a combination of ADC **5** and a suboptimal amount of cisplatin (Fig. 8b, dotted line), cells were initially incubated with cisplatin for 48 h. Following this, serial dilutions of ADC **5** were prepared in growth medium and cells were treated with ADC **5**. In the presence of a sub-optimal amount of cisplatin (Fig. 8b, dotted line), ADC **5** reduced cell viability significantly, affording an IC_50_ value of 15 pM. In comparison to the combination approach, ADC **5** (plus cathepsin B) showed relatively moderate toxicity with IC_50_ values of 0.9 nM. These results demonstrate that the drug release process could be improved with progression of cancer cell death, which is amplified in the presence of an additional chemotherapeutic agent such as in the case of cisplatin.

### Target engagement *in vivo*

**Fig. 9:**
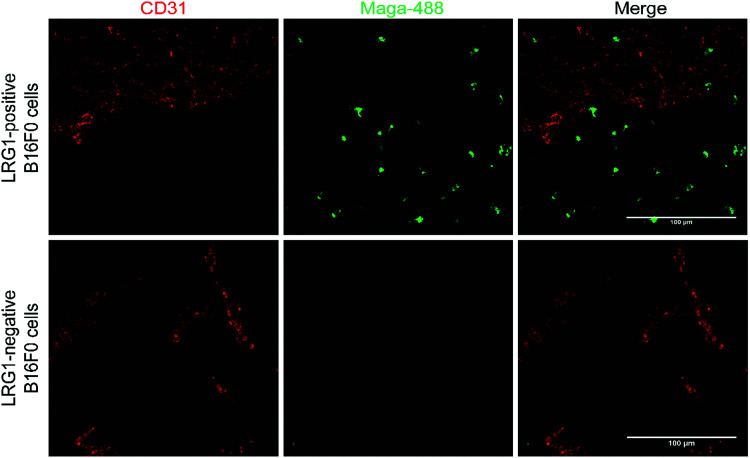
Magacizumab-488 **3a** accumulates in tumours expressing hLRG1. Sections collected from mice receiving a single injection of Maga-488 **3a** at a dose of 100 μg. After ADC was in circulation, tumours were excised, sectioned and subjected to immunofluorescence staining. Blood vessels were stained using anti-CD31 (red). Scale bars, 100 μm.

Having demonstrated that site-selectively modified Magacizumab (Maga-488 **3a**) retains its ability to bind to its target antigen *in vitro*, we next evaluated whether Magacizumab is able to bind to hLRG1 and localise to tumours *in vivo*. Human LRG1 knock-in (KI) mice were injected subcutaneously with hLRG1-negative or hLRG1-positive B16F0 mouse melanoma cells into the lower back and left to develop into tumours. When the tumours became palpable, labelled Maga-488 **3a** was injected into the peritoneum. After 4 h, mice were sacrificed and the tumours and livers excised for analysis by fluorescence microscopy. Analysis of tumour tissues revealed selective localisation and targeted binding of Maga-488 **3a** in hLRG1-expressing tumours in contrast to the non-hLRG1 expressing tumours (Fig. 9). Furthermore, analysis of liver tissue revealed no signal for Maga488 **3a** in the liver (see Fig. S23, ESI†), indicating that Magacizumab is specifically targeted at the tumour. These results also provide evidence of LRG1 presence specifically within the tumour environment, further validating LRG1 as a target for a non-internalising ADC. LRG1 is primarily synthesised by the liver and secreted into circulation,^55^ however as the dipeptide val-cit linker employed in ADC **5** is sensitive to cleavage by lysosomal proteases (*e.g.* cathepsin B), which are commonly observed to have overexpression and extracellular activity exclusively within the tumour microenvironment,^56,57^ it was not expected to cause any off-target toxicity. Furthermore, preliminary data from our labs has shown circulation levels of LRG1 to be much lower than in the tumour, providing a rationale for the targeting of LRG1 for cancer therapy.

### *In vivo* proof-of-concept therapy studies

Having demonstrated *in vivo* target engagement of the humanised anti-LRG1 antibody Magacizumab to an experimental hLRG1 expressing tumour we undertook an *in vivo* study to evaluate the efficacy of hLRG1-targeting ADC **5** in tumour-bearing mice. A fundamental aspect of the use of ADCs as anti-cancer agents is to limit the potential for off-target toxicity. However, ADCs can exert unexpected dose-limiting toxicities which can be mediated *via* any of the components of the drug. We therefore compared our ADC to standard treatment to ensure that the toxicity for the ADC was equivalent or less than that of standard treatment.^58^

Hence, a study was conducted where the novel ADC **5** was compared against a number of treatments including a suboptimal dosing regimen of the native parent antibody Magacizumab and a maximum tolerated dose, as determined by O’Connor *et al.*^21^of the chemotherapeutic agent cisplatin. In order to obtain more insights into the susceptibility of ADC products to proteolytic cleavage, ADC **5** was also investigated in combination with the chemotherapeutic agent cisplatin, where the ADC and cisplatin were administered as a single injection at the same time. In accordance with most ADC therapy studies, treatment in this study was initiated when tumours were palpable in order to be clinically relevant.

**Fig. 10:**
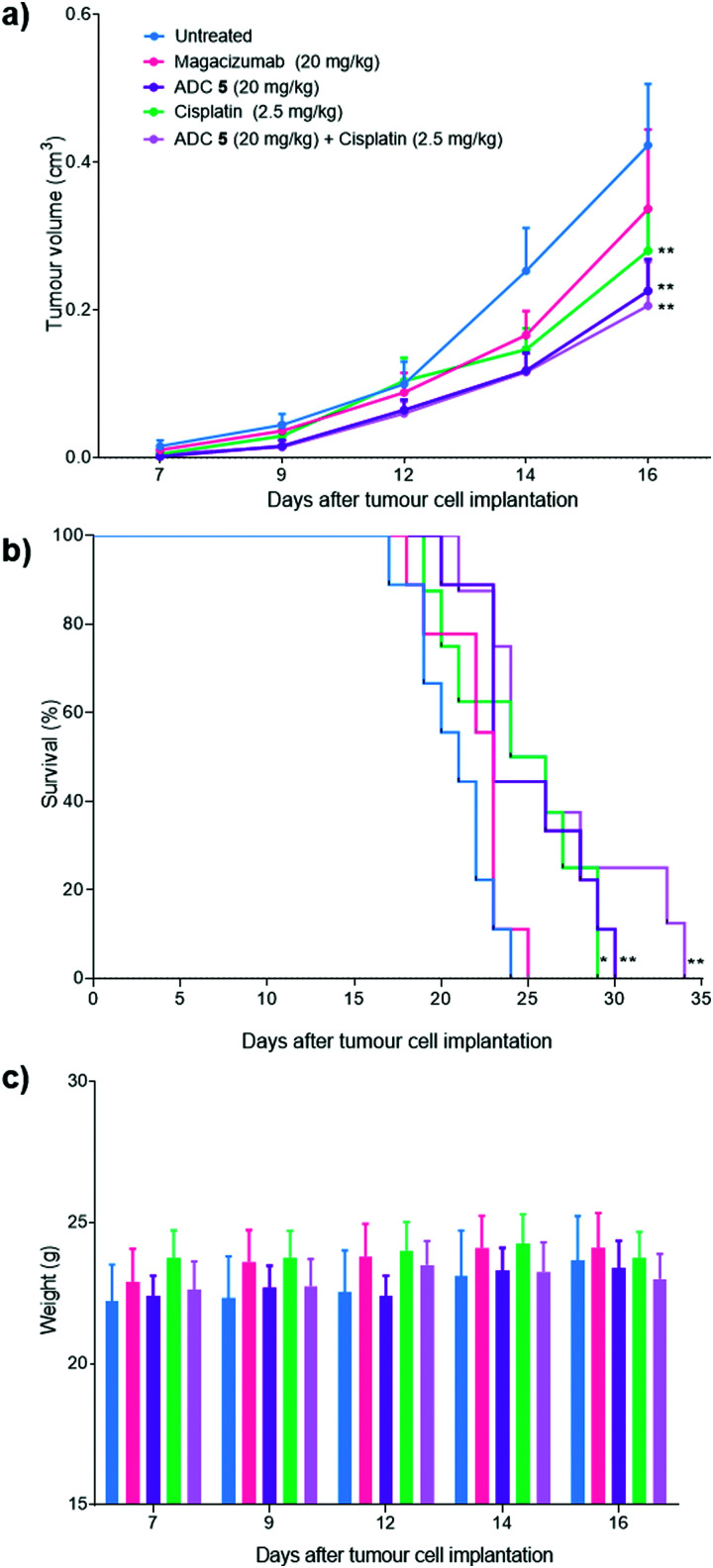
Therapeutic efficacy of ADC **5** in a mouse melanoma model. (a) 6–11 week old hLRG1 knock-in mice bearing subcutaneous hLRG1-expressing B16F0 tumours were treated intraperitoneally with 20 mg kg^−1^ of ADC **5** (*n* = 9), 20 mg kg^−1^ of Magacizumab (*n* = 9), 2.5 mg kg^−1^ of Cisplatin (*n* = 8) or left untreated (*n* = 9). Treatment was performed once every 7 days for a period of three weeks. Therapy was initiated when tumours reached a size of 0.1 cm^3^. (a) Data represents mean tumour volumes, expressed as mean ± SEM (**P < 0.01, two-way ANOVA with Dunnett's multiple comparisons). Tumour growth curves are displayed until day 16, when the first mouse was sacrificed. (b) Survival curves of treatment and control groups; prolongation of survival for mice that received cisplatin (*P < 0.05), ADC **5** (**P < 0.01) and combination therapy (**P < 0.01) as determined by the Log rank test. (c) Body weight variations of the mice during therapy. No detectable weight loss was observed.

Magacizumab and ADC **5** were administered at 20 mg kg^−1^ and cisplatin at 2.5 mg kg^−1^*via* intraperitoneal (i.p.) injection once every 7 days for a total of three doses for mice in the treatment groups. This dosing regimen for the ADC is in line with other interchain disulfide bridging ADCs.^38,59^ Such a high injected dose of ADC would also ensure that the injected dose is sufficient to exceed what may be adsorbed by the circulating LRG1 pool. This dose of cisplatin was selected as it has been demonstrated as the maximum tolerated dose by O’Connor *et al.*^21^ in the B16F0 subcutaneous model. Tumours were measured and mice were weighed regularly throughout the duration of the study. Measurements of tumour volumes showed a significant tumour growth retardation was observed for mice receiving ADC, cisplatin and combination therapy (ADC **5** + cisplatin) (Fig. 10a, *P* < 0.01, two-way ANOVA with Dunnett's multiple comparison test). Survival analysis reflected this result and revealed that treatment led to a significant prolongation of survival for mice that received cisplatin (*P* < 0.05), ADC **5** (*P* < 0.01) and ADC **5** + cisplatin (*P* < 0.01). As corroborative evidence, we also probed for apoptosis in tumour sections and found there to be increased apoptosis in ADC-treated tumours compared to untreated and Magacizumab-treated tumours. This suggests that the cytotoxic component of the ADC leads to increased cell death in the tumour relative to Magacizumab alone (see Fig. S24, ESI†).

Magacizumab alone resulted in a non-significant reduction in tumour growth (Fig. 10a). The lack of effectiveness contrasts with a previous report showing significant tumour inhibition following LRG1 blockade with the parent antibody 15C4.^21^ This is most likely due to the sub-optimal dose of 20 mg kg^−1^ of Magacizumab used in this study and the less frequent administration. However, ADC **5** administered on its own and in combination with cisplatin gave a pronounced tumour growth delay which was evident even after receiving just a single treatment dose, and extended overall survival from 24 days as seen for untreated mice to 30 and 34 days respectively (Fig. 10b). These results are comparable to the survival of mice that received the chemotherapeutic agent cisplatin on its own. When mice were treated with a therapeutic combination of ADC **5** and cisplatin, the effect of ADC **5** was amplified, although not significantly. The difference in efficacy for this group between the *in vitro* and the *in vivo* study can possibly be attributed to the differences in dosing regime; *i.e.* sequential treatment with cisplatin followed by ADC **5** in the *in vitro* study in comparison to simultaneous treatment with cisplatin and ADC **5** in a single injection for the *in vivo* study. Cisplatin-induced tumour cell death in these mice could have resulted in increased amounts of appropriate proteases (*e.g.* cathepsin B) being released into the tumour environment, which would then amplify the release of more free MMAE from the non-internalising ADC **5** and lead to an increase in survival. Although all treatments were tolerated throughout the duration of the study, with no significant weight loss observed (Fig. 10c), alterations in weight were detected in 20–40% of all mice receiving cisplatin as a monotherapy or in combination with the ADC. These mice displayed between 10% and 15% weight loss (mice were sacrificed if weight loss exceeded >15%) and also appeared to show signs of fatigue which could be attributed to the off-target toxicity caused by cisplatin. Although the ADC was not curative, which could in part be explained by the non-internalising nature of LRG1 and that more work needs to be carried out as it is such a novel target, this is the first experimental demonstration that an extracellular hLRG1-targeting ADC can be used to mediate antitumor activity *in vivo*. Different dosing regimes and drug loadings can certainly be considered in future studies.

Although the vc-PABC-based linkers have been shown to exhibit long term instability in rodent plasma studies, which may impact rodent-based pre-clinical studies,^18,60–62^ we note that this is often in a way that reduces the ‘true’ efficacy of the ADC,^53–55^ which could explain the lower than expected efficacy of our ADC as it was not tested in Ces1C knock-out mice.^48,62^ Nevertheless, we show that our ADC was active *in vivo*, especially when compared to various control groups. Moreover, we demonstrate that on this novel target, the ADC exhibited similar efficacy to an existing chemotherapeutic without the off-target effects associated with non-targeted delivery of cisplatin, and that combination therapy could be an interesting strategy to increase the potency of this non-internalising ADC.

## Conclusion

ADCs have the potential to improve the treatment of tumours over chemotherapy as they allow the use of toxins with orders of magnitude greater potencies to be delivered in a selective manner. Current FDA-approved ADCs target tumour cell surface markers and rely on ADC internalisation into the cells for drug delivery. Instead, the approach reported herein relies on targeting the novel extracellular vasculopathic factor LRG1, offering coverage of a wide range of tumours as LRG1 is induced in disease lesions where it disrupts the angiogenic process through interfering with vessel maturation, a common feature of many tumours. By applying the disulfide-rebridging PD scaffold to the hinge-stabilised hLRG1-targeting IgG4 antibody Magacizumab, we demonstrated that this ADC retains binding post-modification, displays excellent serum stability and is effective in *in vitro* cell viability assays. We demonstrate that LRG1 and Magacizumab-LRG1 are predominantly non-internalising, and that a Magacizumab-fluorophore conjugate localises at the tumour site in an *in vivo* model. We also report that upon binding to the novel target LRG1 in the tumour environment, the ADC liberates its cytotoxic payload and shows, *in vivo*, enhanced tumour activity relative to antibody alone and similar anti-tumour activity when compared against the maximum tolerated dose of the chemotherapeutic agent cisplatin but crucially without any undesired side-effects and with scope for higher/more frequent dosage of the ADC to gain further efficacy in future studies. The ADC presented here allows the progressive amplification of drug release, which is linked to the antibody *via* a cleavable dipeptide linker, presumably upon being metabolised by appropriate proteases (*e.g.* cathepsin B), which are released into the surrounding tissue upon induction of tumour cell death. This effect seems to be amplified by administration of a therapeutic combination of ADC and cisplatin. Overall, the proof-of-concept findings of this study provide novel opportunities for cancer therapy. They are especially significant in the context of the need for finding novel ADC targets (in general) but are particularly pertinent in this study as the target we exploit is a common feature of virtually all types of aggressive solid cancers (*i.e.* LRG1 in abnormal angiogenesis).

## Materials and methods

### General experimental

All chemical reagents were purchased from Sigma Aldrich, ThermoFisher Scientific, Molecular probes, Alfa Aesar, Acros, New England Biolabs and Medchem Express. Compounds and solvents were used as received. Petrol refers to petroleum ether (b.p. 40–60 °C). Chemical reactions were monitored using thin layer chromatography (TLC) on pre-coated silica gel plates (254 μm) purchased from VWR. Flash column chromatography was carried out with pre-loaded GraceResolv^TM^ flash cartridges on a Biotage® Isolera Spektra One flash chromatography system (Biotage®). ^1^H NMR spectra were obtained at 600 MHz or 700 MHz. ^13^C NMR spectra were obtained at 150 MHz or 175 MHz. All results were obtained using Bruker NMR instruments, the models are as follows: Avance III 600, Avance Neo 700. All samples were run at the default number of scans and at 21 °C. Chemical shifts (*δ*) for ^1^H NMR and ^13^C NMR are quoted on a parts per million (ppm) scale relative to tetramethylsilane (TMS), calibrated using residual signals of the solvent. Where amide rotamers are the case, and when possible, only the chemical shifts of the major rotamer has been assigned and areas underneath all rotameric peaks have been considered for the integral intensity calculations. Coupling constants (*J* values) are reported in Hertz (Hz) and are reported as *J*_H–H_ couplings between protons. Infrared spectra were obtained on a PerkinElmer Spectrum 100 FTIR spectrometer operating in ATR mode. Melting points were measured with Gallenkamp apparatus and are uncorrected.

### Formation and characterisation of antibody–fluorophore conjugate **3** (AFC **3**) and antibody–drug conjugate **5** (ADC **5**)

See Supplementary Methods in the ESI.†

### Cell culture

B16F0 mouse melanoma cells (ATCC) were maintained in Dulbecco's Modified Eagle's Medium (DMEM) supplemented with glucose (4.5 g L^−1^), sodium pyruvate (110 mg L^−1^), 10% Fetal bovine serum (FBS), penicillin (10 000 U L^−1^) and streptomycin sulphate (100 mg L^−1^). Transfected B16F0 cells were also cultured as described above and additionally supplemented with G418 antibiotic (1.0 mg mL^−1^). All cultures were maintained at 37 °C in 5% CO_2_ and checked to be clear of contamination. For cell culture experiments, sterile PBS was purchased from ThermoFisher.

### Human *Lrg1* expression in mammalian cells

See Supplementary Methods in ESI.†

### *In vitro* Internalization analysis by confocal microscopy

hLRG1-transfected and wild type B16F0 cells on coverslips at 70% confluency were incubated with labelled constructs at 10 μg mL^−1^ for 1 h at 4 °C and then at 37 °C. Cells were washed three times with PBS to remove unbound antibodies followed by fixation with 4% formaldehyde for 15 min at room temperature. Coverslips were permeabilised and blocked as described previously. Actin was detected with phalloidin-568 (Invitrogen) and DAPI was used to stain cell nuclei.

As a positive control, cells were incubated with Transferrin-555 at 4 °C and then incubated at 37 °C. Cells were fixed and stained with phalloidin-680 for actin and DAPI for nuclear staining.

### *In vitro* cytotoxicity assessment

*In vitro* cytotoxicity of compounds was evaluated in hLRG1-positive and wild type B16F0 cell lines by the MTT [3-(4,5-dimethylthiazol-2-yl)-2,5-diphenyltetrazolium bromide] colourimetric assay. Briefly, 5 × 10^4^ cells were seeded in 96-well plates and incubated at 37 °C overnight. Cells were then exposed to a range of concentrations of the test compounds diluted in growth medium at pH 6.5 and at 37 °C as follows: MMAE (0–100 nM, 72 h), Cisplatin (0–66 μM, 48 h), Magacizumab (0–100 nM, 72 h), N_3_-PEG_3_-vc-PABC-MMAE (0–100 nM, 72 h) and ADC **5** (0–100 nM, 72 h). Following each treatment, cells were washed twice with PBS and the medium was replaced with growth medium free of phenol red. MTT reagent (12 mM) was then added to each well and cells were incubated for 4 h at 37 °C, followed by the addition of DMSO and further incubation at 37 °C for 1 h. Optical density (OD) was measured at 540 nm. The percentage of viable cells was calculated as follows: cell viability (%) = ((OD_treated cells_ − OD_blank_)/(OD_untreated cells_)) × 100.

ADC **5** + Cathepsin B:^18^ 3.3μL of Cathepsin B (human liver, Sigma-Aldrich, 13.8 μM) was added to 11.7 μL sodium acetate buffer (2.2 M, pH = 5.8), 24 μL 30 mM DTT and 1 μL 500 mM EDTA. The resulting mixture was activated by incubation at room temperature for 15 min, then 360 μL sodium acetate buffered medium (pH 6.5) was added and to this solution was added ADC **5** (100 μL at 10 μM). Cells were treated with this ADC **5** + Cathepsin B mixture as described above for ADC **5** (0–100 nM, 72 h at 37 °C).

ADC **5** + Cisplatin: Cells were initially incubated with 5 μM of Cisplatin for 48 h. Following this, serial dilutions of ADC **5** (0–100 nM) were prepared in growth medium and cells were treated as described for ADC **5** (72 h at 37 °C).

### Tumour models

C57BL/6 mice were purchased from Charles River Laboratories. Human LRG1 knock-in mice were generated by the Moss and Greenwood laboratories as described by Kallenberg *et al.*^29^ Single-cell suspensions of 1 × 10^6^ B16F0 cells (human-LRG1 transfected and wild type cells) were injected subcutaneously into the lower back of *Lrg1*^+/+^ C57BL/6 mice in 100 μL PBS. Mice were randomised by age prior to inoculation. Tumours were measured at defined intervals using calipers and tumour volume was calculated using the formula: *V* = 4π/3 (1/2 length × 1/2 width × 1/2 height). Mice were sacrificed at the end of the experiment, or when tumours reached a maximum of 1.5 cm^3^ or weight loss exceeded 15% of the total body weight. All procedures were performed in accordance with the UK Animals (Scientific Procedures) Act and the Animal Welfare and the Ethical Review Bodies of the UCL Institute of Ophthalmology.

### Therapy studies

*Lrg1* ^+/+^ C57BL/6 mice (age, 6–11 wk; weight, 19–31 g) were injected subcutaneously into the back with single-cell suspensions of 1 × 10^6^ hLRG1-transfected B16F0 cells in 100 μL PBS. Animals were randomised and allocated to the following groups prior to treatment: (1) untreated (*n* = 9); (2) Magacizumab (*n* = 9); (3) Cisplatin (*n* = 8); (4) Magacizumab PD-MMAE ADC **5** (*n* = 9); (5) Magacizumab + Cisplatin (*n* = 7); (6) ADC **5** + Cisplatin (*n* = 8). Tumours were measured and therapy was initiated when tumour volumes reached 0.1 cm^3^. Magacizumab and ADC **5** were administered at a dose of 20 mg kg^−1^, and cisplatin at 2.5 mg kg^−1^. All treatments were administered by a single intraperitoneal injection every 7 days for 3 weeks. Tumour volumes were measured, and mouse weights were monitored throughout the duration of the study. In addition to weight loss, disease progression was also evaluated qualitatively by observation of behaviour and muscle wasting. Tumour growth curves and survival curves were used to evaluate treatment efficacy.

### Immunofluorescence studies

Subcutaneous B16F0 tumour models were fresh frozen on dry ice in Optimal cutting temperature compound (OCT). Contiguous frozen tissue sections were cut at a thickness of 10 μm for therapy studies and 30 μm for localisation studies and stored at −80 °C. Sections were fixed in 4% paraformaldehyde for 15 min at room temperature or 100% methanol for 5 min at −20 °C, depending on antibodies used. After this, sections were washed with PBS and permeabilised with 0.1% Tween in PBS for 10 min. Sections were blocked in 1% BSA prior to overnight incubation with primary antibody at 4 °C. Sections were washed in 0.01% Tween-20 in PBS and incubated with secondary antibodies for 1h at room temperature. Antibodies used to label mouse endothelium were anti-CD31 (Dianova), mouse cathepsin B was labelled using an anti-cathepsin B polyclonal antibody (Invitrogen) and apoptosis was measured using an anti-γ-h2ax antibody (Abcam). Alexa-fluor labelled secondary antibodies were from Thermofisher. Sections were imaged using a using Zeiss 710 confocal microscope. Maximum intensity projections of z-stacks were analysed using NIS elements software (Nikon).

### Statistical analysis

Statistical analysis was performed using Graphpad Prism version 6.0 for Windows, Graphpad software (La Jolla California USA, www.graphpad.com). Error bars and statistical tests used for each experiment are indicated in the figure legends. Data are expressed as mean ± standard error of the mean (SEM). A *P* value of less than or equal to 0.05 was considered statistically significant (non-significant *P* > 0.05; *P ≤ 0.05; **P ≤ 0.01; ***P ≤ 0.001; ****P ≤ 0.0001).

## Author contributions

V. C., J. G. and S. E. M. conceived and designed the project; F. J. and C. B. performed the chemistry experiments, F. J. performed the chemical biology experiments; F. J., V. C., J. R. B. and C. B analysed the chemistry and chemical biology experiments; F. J. performed the *in vitro* biology experiments; F. J., C. C., C. P., J. B. and D. K. performed the *in vivo* biology experiments; F. J. and V. C. analysed the data; V. C. and F. J. co-wrote the paper.

## Conflicts of interest

S. E. M. and J. G. are founders of and shareholders in PanAngium Therapeutics that owns the commercialisation rights to Magacizumab. S. E. M. and J. G. are named inventors on patents relating to Magacizumab. V. C. and J. R. B. are directors of the spin-out ThioLogics, but there are no competing financial interests to declare.

## Supplementary Material

CB-002-D1CB00104C-s001
