# Supplementary material for: Leucine-rich alpha-2-glycoprotein 1 (LRG1) as a novel ADC target
Source: RSC Chem Biol. 2021 May 31;2(4):1206–20. doi: 10.1039/d1cb00104c (PMC8341842; doi:10.1039/d1cb00104c)
Supplement: CB-002-D1CB00104C-s001 [file CB-002-D1CB00104C-s001.pdf]

Electronic Supplementary Material (ESI) for RSC Chemical Biology.
This journal is © The Royal Society of Chemistry 2021

General Experimental

All chemical reagents were purchased from Sigma Aldrich, ThermoFisher Scientific, Molecular probes, Alfa Aesar, Acros, New England Biolabs and Medchem Express. Compounds and solvents were used as received. Petrol refers to petroleum ether (b.p. 40–60 °C). Chemical reactions were monitored using thin layer chromatography (TLC) on pre-coated silica gel plates (254 μm) purchased from VWR. Flash column chromatography was carried out with pre-loaded GraceResolv™ flash cartridges on a Biotage® Isolera Spektra One flash chromatography system (Biotage®). ^{1}H NMR spectra were obtained at 600 MHz or 700 MHz. ^{13}C NMR spectra were obtained at 150 MHz or 175 MHz. All results were obtained using Bruker NMR instruments, the models are as follows: Avance III 600, Avance Neo 700. All samples were run at the default number of scans and at 21 °C. Chemical shifts (δ) for ^{1}H NMR and ^{13}C NMR are quoted on a parts per million (ppm) scale relative to tetramethylsilane (TMS), calibrated using residual signals of the solvent. Where amide rotamers are the case, and when possible, only the chemical shifts of the major rotamer has been assigned and areas underneath all rotameric peaks have been considered for the integral intensity calculations. Coupling constants (*J* values) are reported in Hertz (Hz) and are reported as $J_{H\text{-}H}$ couplings between protons. Infrared spectra were obtained on a Perkin Elmer Spectrum 100 FTIR spectrometer operating in ATR mode. Melting points were measured with Gallenkamp apparatus and are uncorrected.

Synthesis of PD 1

Di-*tert*-butyl-1-methylhydrazine-1,2-dicarboxylate [1]

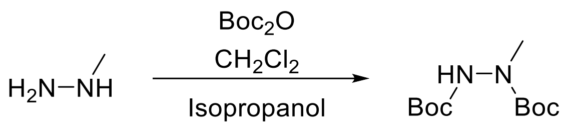

To a solution of methyl hydrazine (1.14 mL, 21.7 mmol) in IPA (16 mL), was added drop wise di-*tert*-butyl dicarbonate (11.85 g, 54.3 mmol) pre-dissolved in in CH_2Cl_2 (12 mL) over 30 min. The reaction was then stirred at 21 °C for 16 h. Following this, the solvents were removed *in vacuo* and the crude residue purified by flash column chromatography (0% to 20% EtOAc/petrol) to afford di-*tert*-butyl-1-methylhydrazine-1,2-dicarboxylate (4.48 g, 18.2 mmol, 84%) as a white solid **m.p.** 58–62 °C (*lit m.p.* 55-56 °C). **^{1}H NMR** (600 MHz, $CDCl_3$) δ 6.41–6.16 (m, 1H) 3.11 (s, 3H), 1.47–1.46 (m, 18H). **^{13}C NMR** (150 MHz, $CDCl_3$) δ 155.9 (C), 81.3 (C), 37.5 (CH_3), 28.3 (CH_3). **IR** (solid) 3315, 2981, 1702 cm^{-1}.

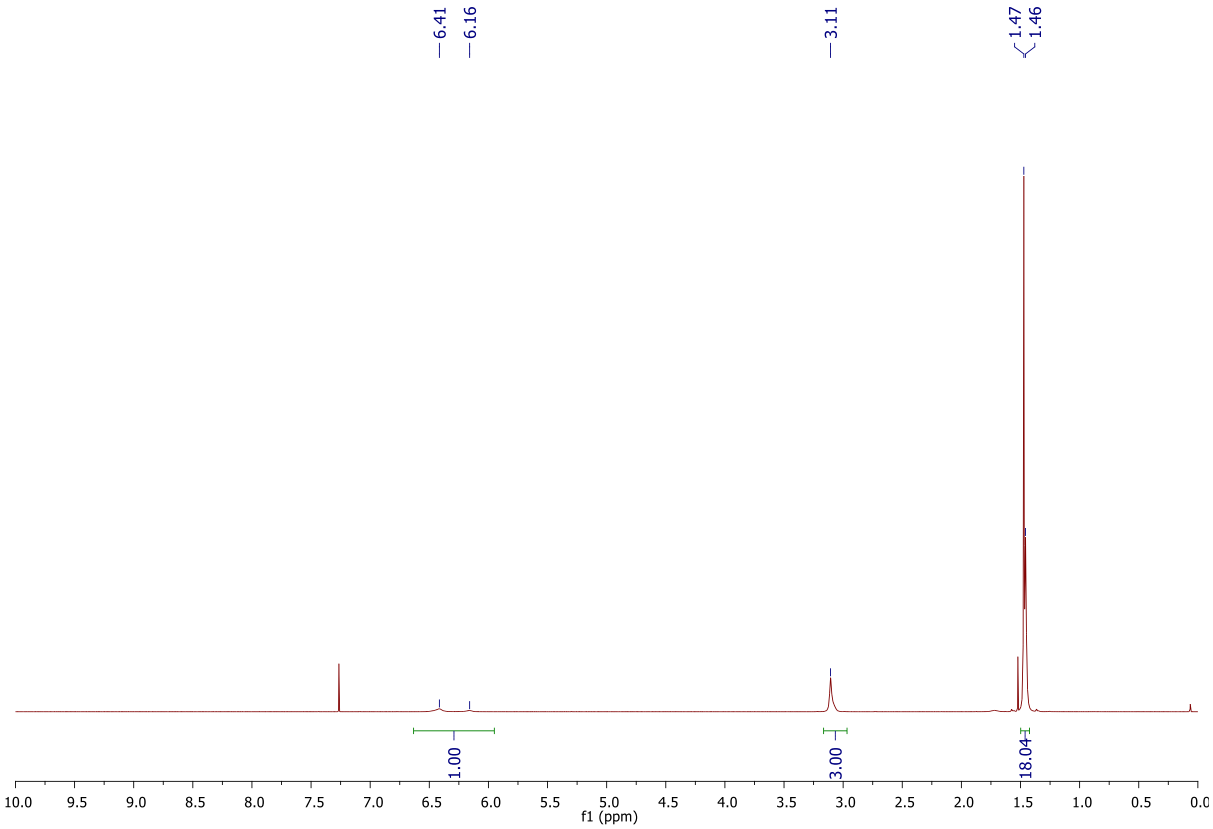

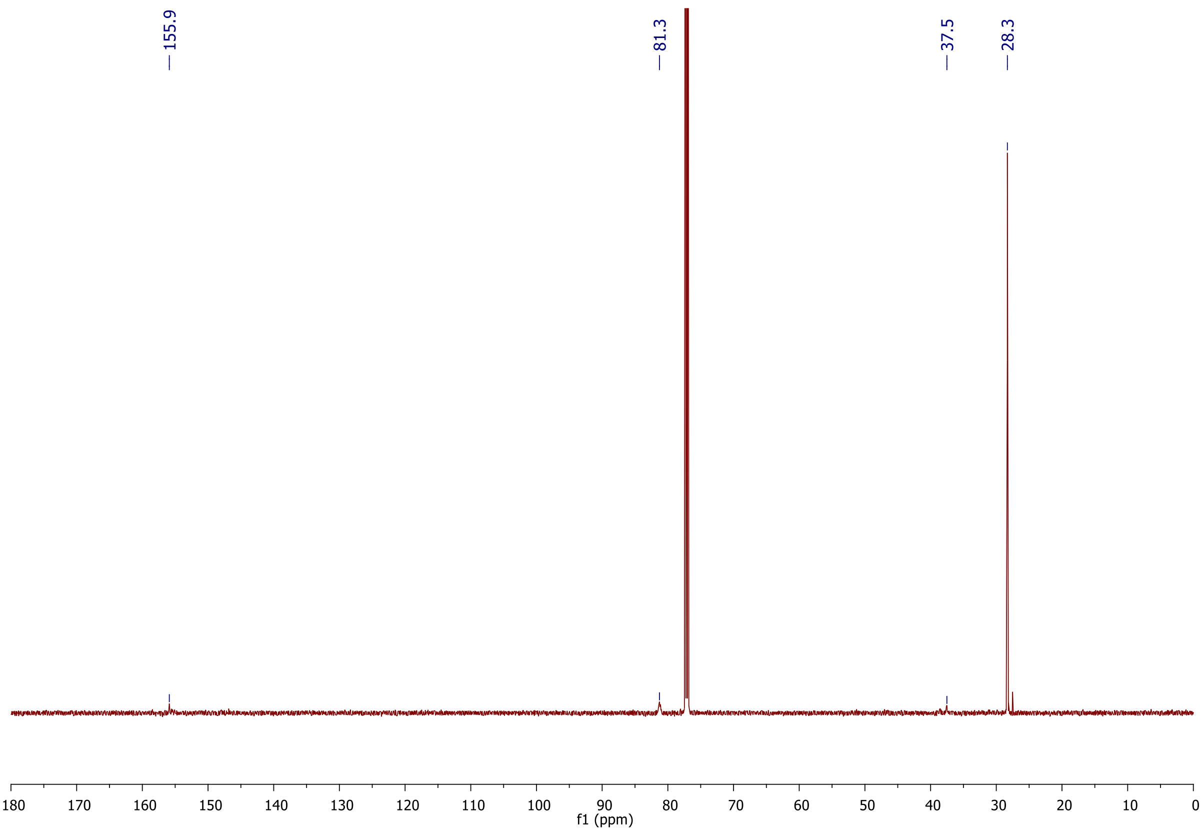

Figure S1 *^{1}H and ^{13}C NMR data for di-tert-butyl-1-methylhydrazine-1,2-dicarboxylate.*

Di-*tert*-butyl-1-(3-(*tert*-butoxy)-3-oxopropyl)-2-methylhydrazine-1,2-dicarboxylate [2]

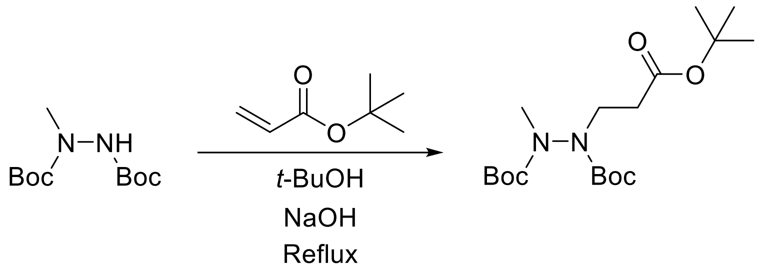

To a solution of di-*tert*-butyl 1-methylhydrazine-1,2-dicarboxylate (3.75 g, 15.2 mmol) in *tert*-butanol (25 mL), was added 0.5 mL of 2 M NaOH and the reaction mixture stirred at 21 °C for 10 min. After this, *tert*-butyl acrylate (6.63 mL, 45.67 mmol) was added to the solution and the reaction mixture was heated under reflux for 72 h. The solvent was then removed *in vacuo* and the crude residue purified by flash column chromatography (0% to 20% EtOAc/petrol) to afford di-*tert*-butyl-1-(3-(*tert*-butoxy)-3-oxopropyl)-2-methylhydrazine-1,2-dicarboxylate (4.73 g, 12.6 mmol, 86%) as a clear oil. **^{1}H NMR** (700 MHz, $CDCl_3$, rotamers) δ 3.84–3.53 (m, 2H), 3.06–2.98 (m, 3H), 2.51 (t, J=7.2 Hz, 2H), 1.47–1.43 (m, 27H). **^{13}C NMR** (175 MHz, $CDCl_3$, rotamers) 171.0 (C), 155.4 (C), 154.4 (C), 81.0 (C), 44.6 (CH_3), 36.6 (CH_2), 34.1 (CH_2), 28.3 (CH_3)). **IR** (thin film) 2976, 2933, 1709 cm^{-1}.

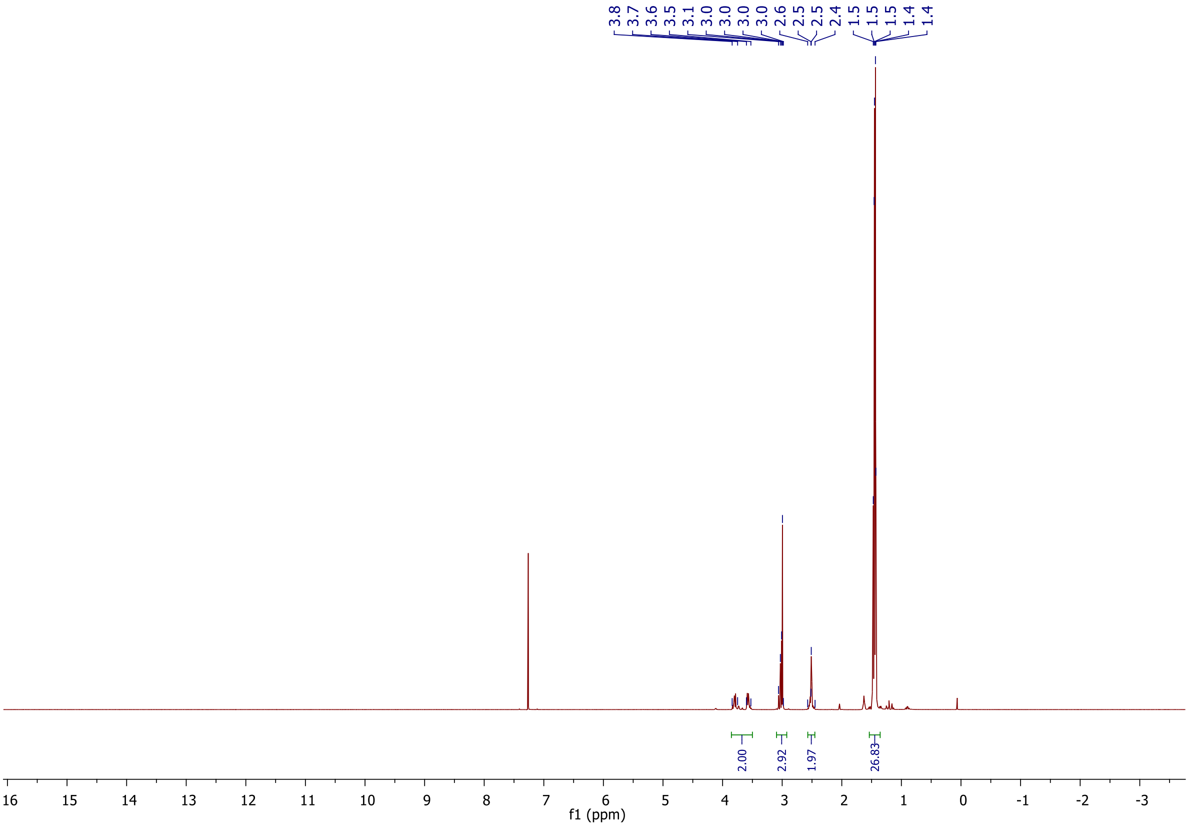
3.8
3.7
3.6
3.5
3.1
3.0
3.0
3.0
3.0
2.6
2.5
2.5
2.4
1.5
1.5
1.5
1.4
1.4
2.00
2.92
1.97
26.83
16
15
14
13
12
11
10
9
8
7
6
5
4
3
2
1
0
-1
-2
-3
f1 (ppm)

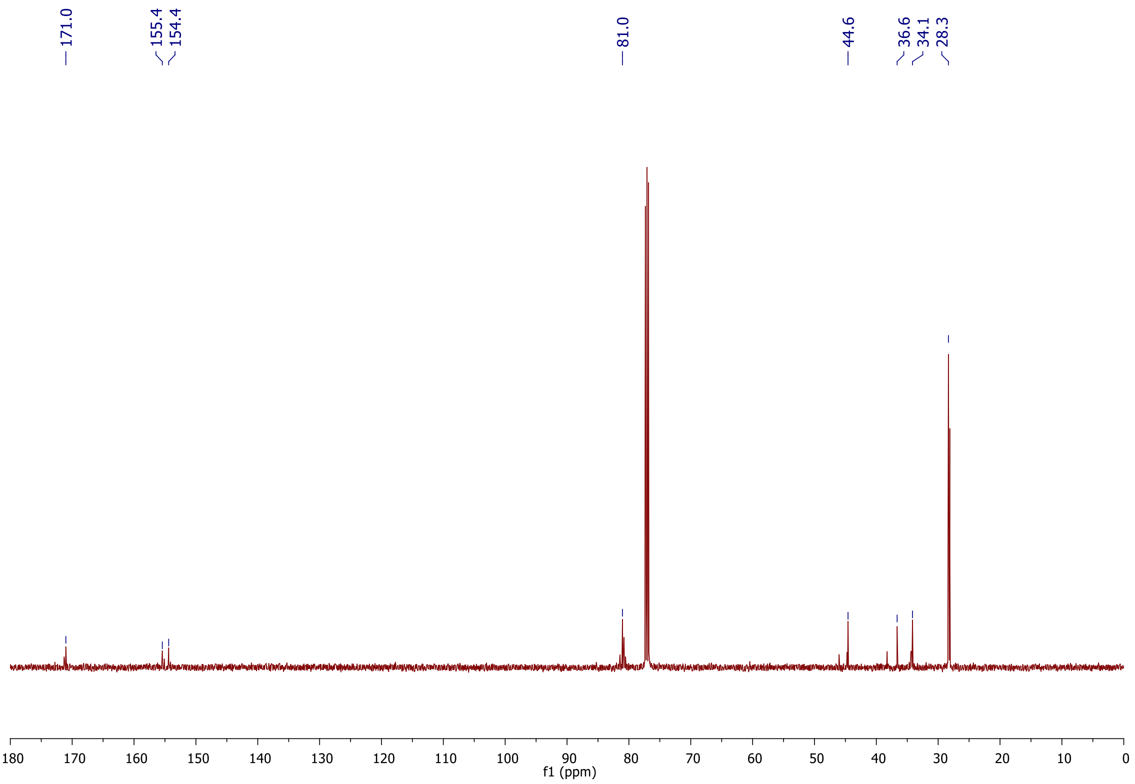

Figure S2 *^{1}H and ^{13}C NMR data for di-tert-butyl-1-(3-(tert-butoxy)-3-oxopropyl)-2-methylhydrazine-1,2-dicarboxylate.*

3-(4,5-Dibromo-2-methyl-3,6-dioxo-3,6-dihydropyridazin-1(2*H*)-yl) propanoic acid [2]

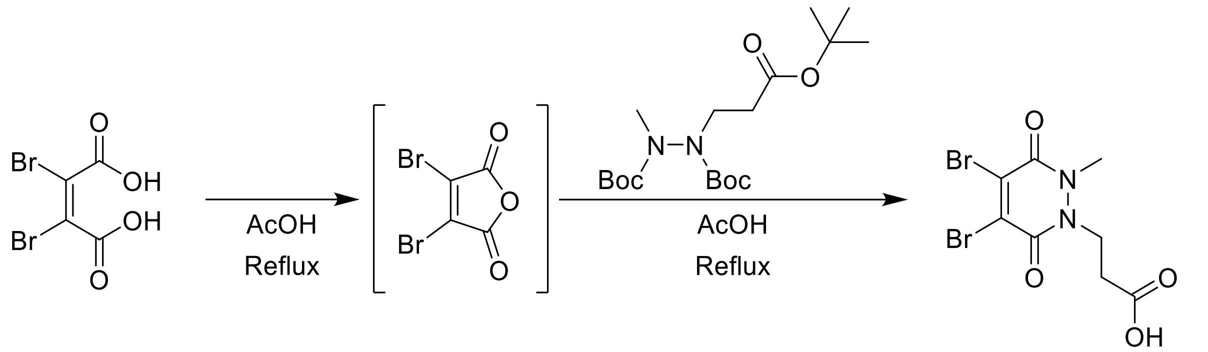

Dibromomaleic acid (1.82 g, 6.68 mmol) was dissolved in AcOH (75 mL) and heated under reflux for 30 min. To this solution, was added di-*tert*-butyl-1-(3-(*tert*-butoxy)-3-oxopropyl)-2-methylhydrazine-1,2-dicarboxylate (3.00 g, 8.01 mmol) and the reaction heated under reflux for a further 4 h. After this time, the reaction mixture was concentrated *in vacuo* with toluene co-evaporation (3 × 30 mL, as an azeotrope) and the crude residue purified by flash column chromatography (50% to 100% EtOAc/petrol (1% AcOH)) to afford 3-(4,5-dibromo-2-methyl-3,6-dioxo-3,6-dihydropyridazin-1(2*H*)-yl) propanoic acid (1.44 g, 4.05 mmol, 61%) as a yellow solid. **m.p.** 139 –142 °C (*lit m.p.* 140–144 °C).[2] **^{1}H NMR** (700 MHz, MeOD) δ 4.44 (t, J = 7.3 Hz, 2H), 3.69 (s, 3H), 2.75 (t, J = 7.3 Hz, 2H). **^{13}C NMR** (175 MHz, MeOD) δ 173.8 (C), 154.8 (C), 154.5 (C), 136.7 (C), 136.4 (C), 44.9 (CH_3), 35.4 (CH_2), 32.6 (CH_2). **IR** (solid) 3044, 1725, 1606, 1570 cm^{-1}.

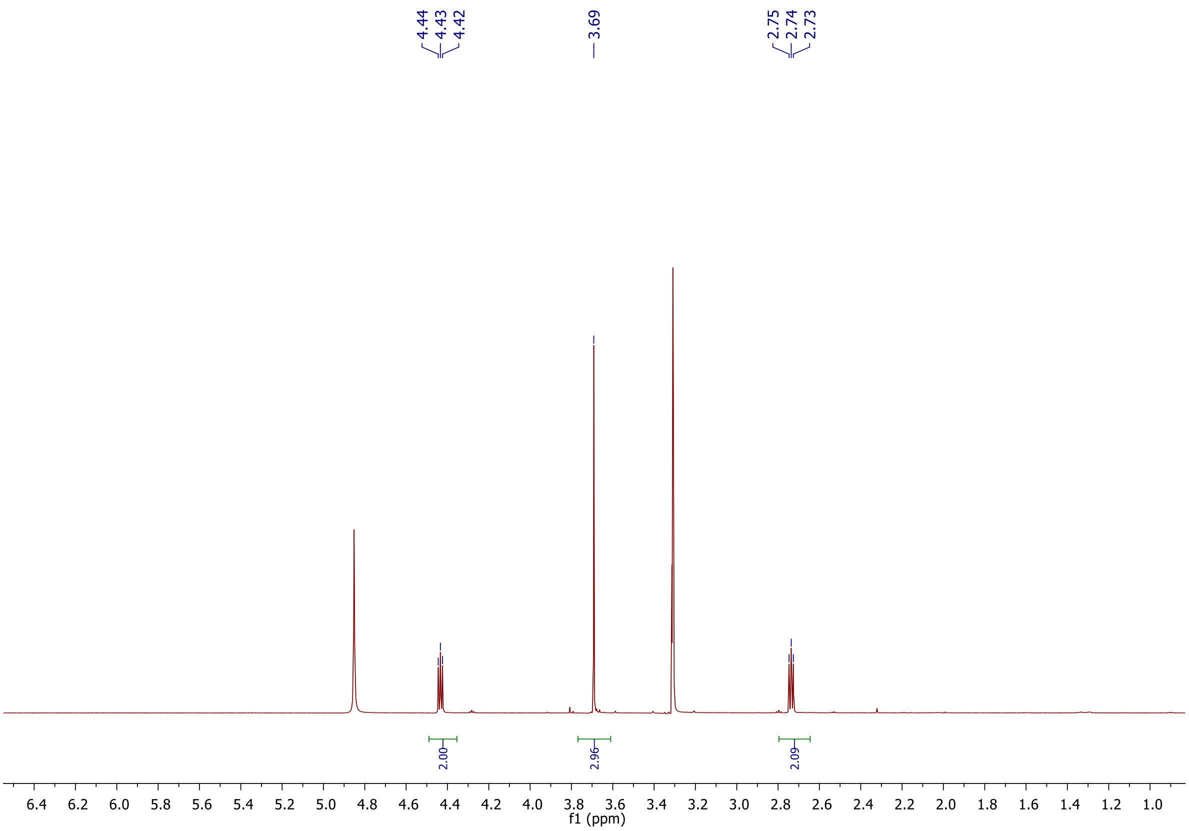

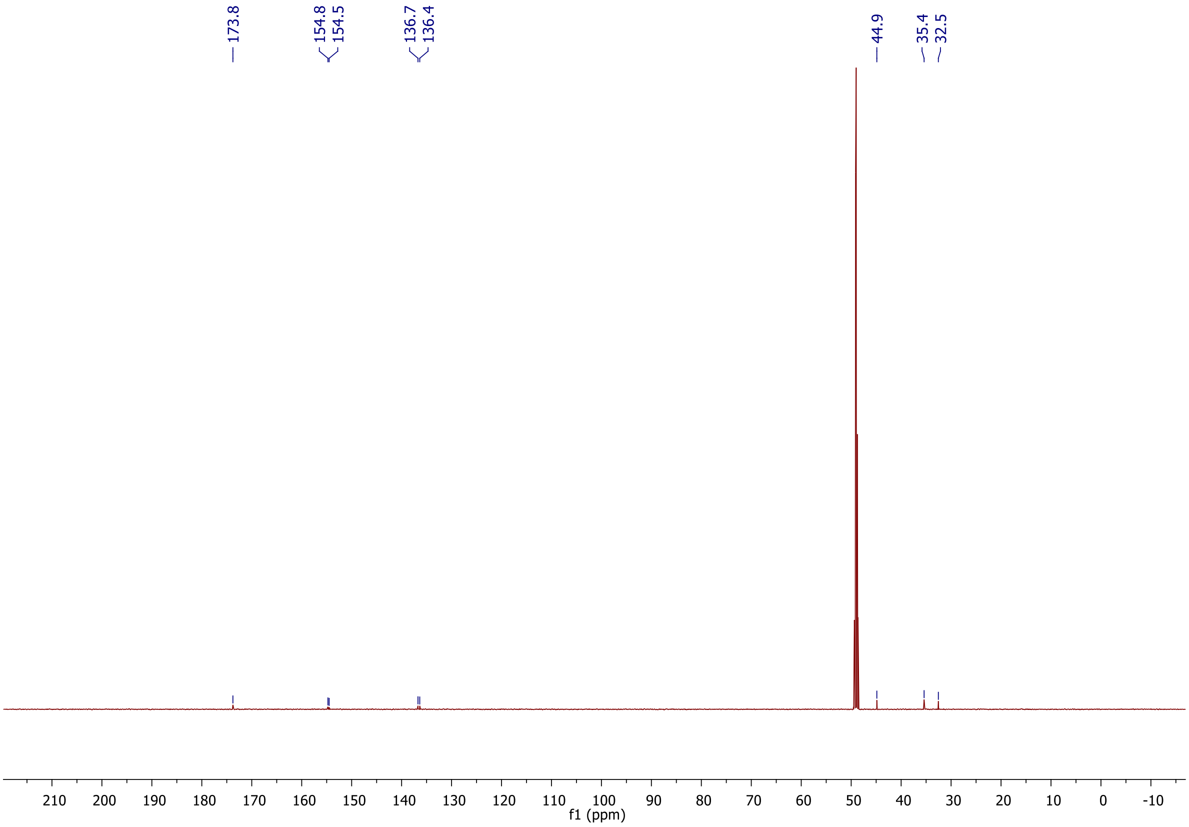

Figure S3 *^{1}H and ^{13}C NMR data for 3-(4,5-dibromo-2-methyl-3,6-dioxo-3,6-dihydropyridazin-1(2H)-yl) propanoic acid.*

2,5-Dioxopyrrolidin-1-yl 3-(4,5-dibromo-2-methyl-3,6-dioxo-3,6-dihydropyridazin-1(2*H*)-yl) propanoate [2]

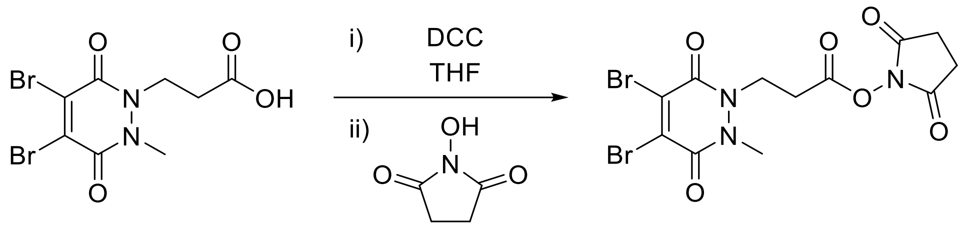

To a solution of 3-(4,5-dibromo-2-methyl-3,6-dioxo-3,6-dihydropyridazin-1(2*H*)-yl) propanoic acid (700 mg, 1.97 mmol) in THF (20 mL), pre-cooled to 0 °C, was added *N,N'*-dicyclohexylcarbodiimide (445.7 mg, 2.16 mmol). The homogenous solution was then stirred at 0 °C for 30 min. Following this, was added *N*-hydroxysuccinimide (249 mg, 2.16 mmol) and the reaction stirred at 21 °C for a further 16 h. The newly formed heterogenous mixture was then filtered and the filtrate concentrated *in vacuo*. Purification of the crude residue by flash column chromatography (30% to 100% EtOAc/petrol) afforded 2,5-dioxopyrrolidin-1-yl 3-(4,5-dibromo-2-methyl-3,6-dioxo-3,6-dihydropyridazin-1(2*H*)-yl) propanoate (70 mg, 0.15 mmol, 8%) as a white solid. **m.p.** 101–104 °C (*lit m.p.* 100–104 °C). **^{1}H NMR** (700 MHz, $CDCl_3$) δ 4.48 (t, *J* = 6.9 Hz, 2H), 3.68 (s, 3H), 3.10 (t, *J* = 6.9 Hz, 2H), 2.85 (s, 4H). **^{13}C NMR** (175 MHz, $CDCl_3$) δ 168.7 (C), 166.0 (C), 153.4 (C), 153.2 (C), 136.9 (C), 135.3 (C), 43.0 (CH_2), 35.3 (CH_3), 29.1 (CH_2), 25.7 (CH_2). **IR** (solid) 2992, 1814, 1782, 1735, 1634, 1576 cm^{-1}.

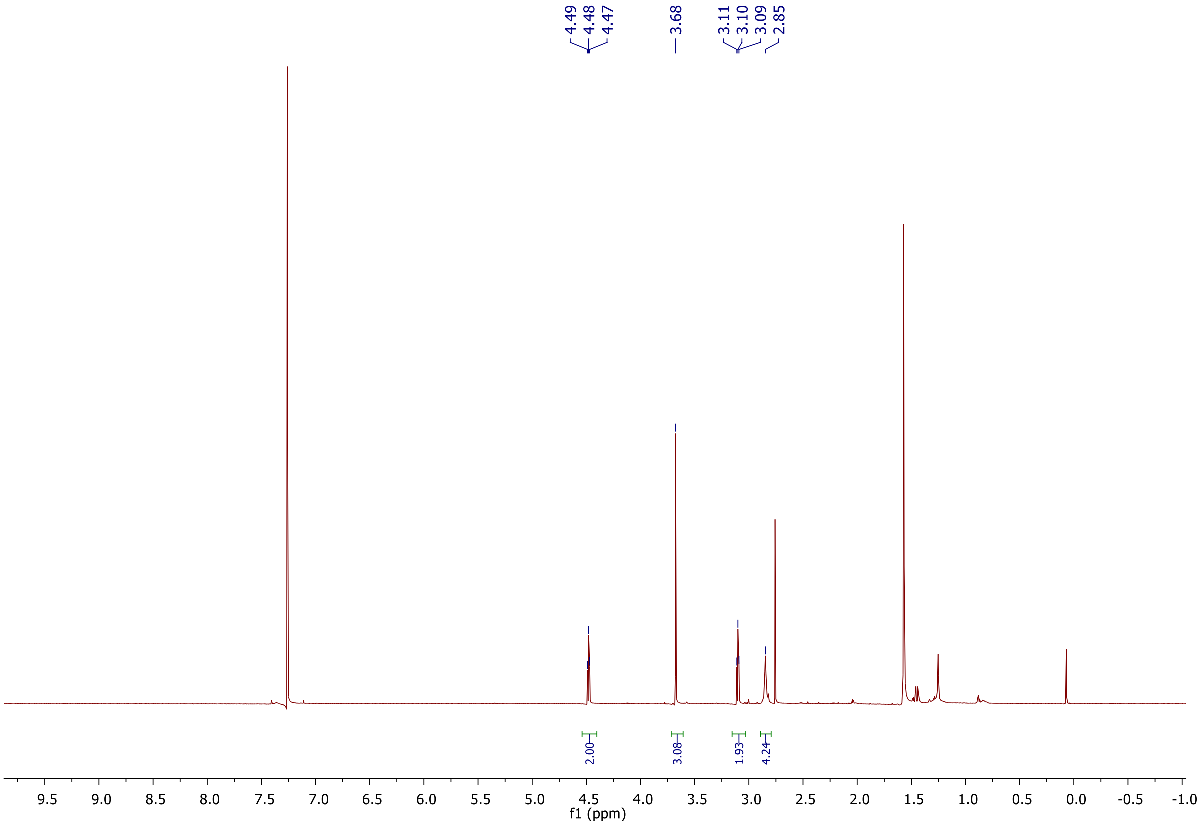

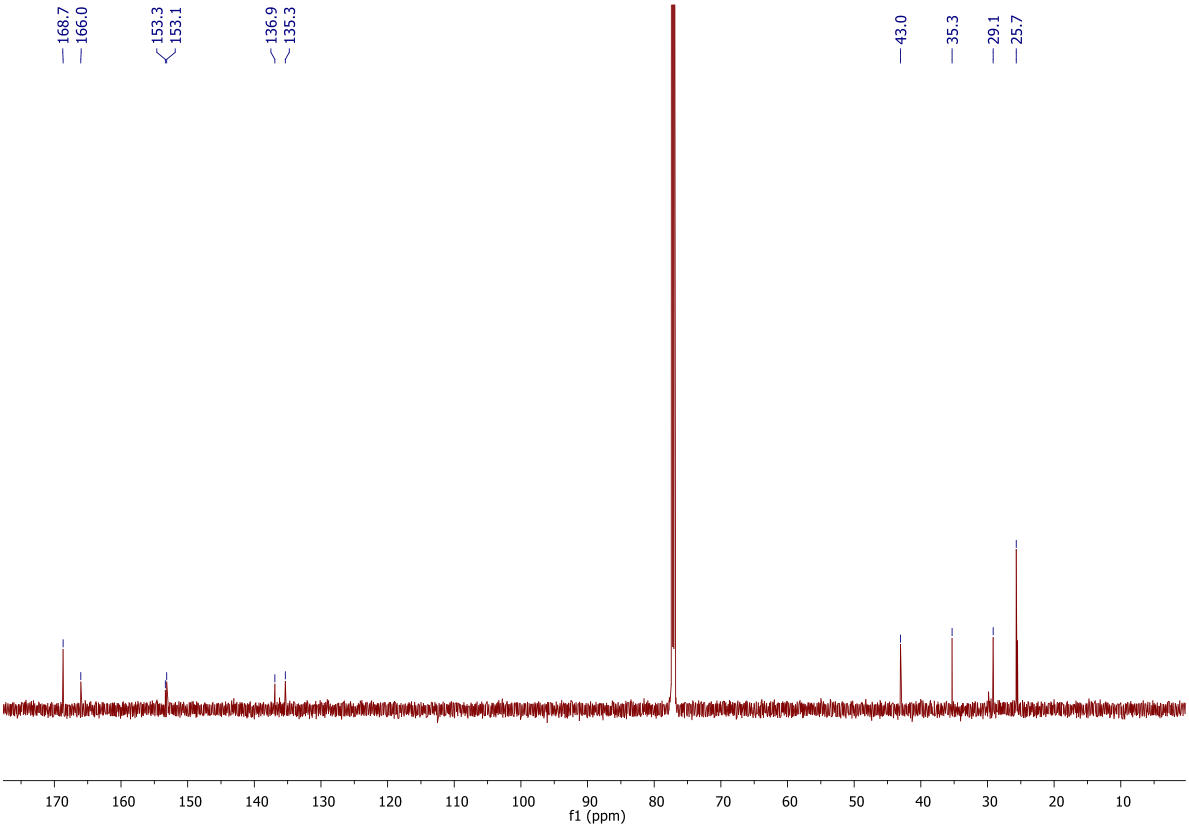

Figure S4 *^{1}H and ^{13}C NMR data for 2,5-dioxopyrrolidin-1-yl 3-(4,5-dibromo-2-methyl-3,6-dioxo-3,6-dihydropyridazin-1(2H)-yl) propanoate.*

((1*R*,8*S*,9*S*)-Bicyclo[6.1.0]non-4-yn-9-yl)methyl (2-(2-(2-(3-(4,5-dibromo-2-methyl-3,6-dioxo-3,6-dihydropyridazin-1(2*H*)-yl)propanamido)ethoxy)ethoxy)ethyl) carbamate [2]

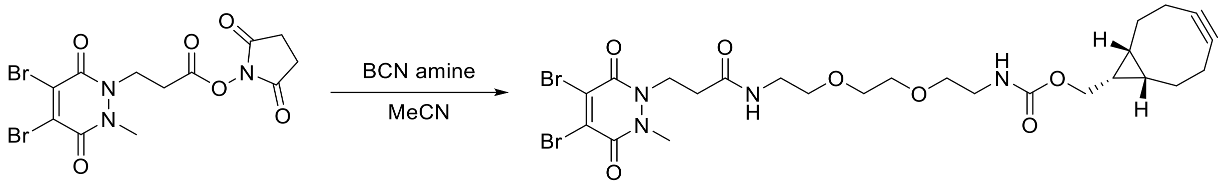

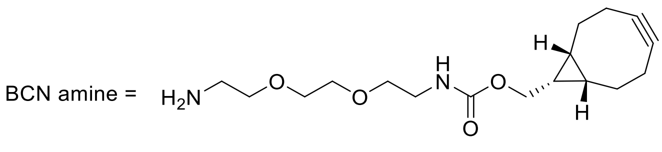

To a solution of 2,5-dioxopyrrolidin-1-yl 3-(4,5-dibromo-2-methyl-3,6-dioxo-3,6-dihydropyridazin-1(2*H*)-yl) propanoate (70 mg, 0.15 mmol) in MeCN (10 mL) was added *N*-[(1*R*,8*S*,9*S*)-bicyclo[6.1.0]non-4-yn-9-ylmethyloxycarbonyl]-1,8-diamino-3,6-dioxaoctane (50 mg, 0.15 mmol) and the reaction stirred at 21 °C for 16 h. After this time, MeCN was removed *in vacuo* and the crude residue dissolved in $CHCl_3$ (50 mL), and washed with water (2 × 30 mL) followed by saturated aq. K_2CO_3 (30 mL). The organic layer was then dried ($MgSO_4$) and concentrated *in vacuo.* Purification of the crude residue by flash column chromatography (0% to 10% MeOH/EtOAc) afforded ((1*R*,8*S*,9*S*)-Bicyclo[6.1.0]non-4-yn-9-yl)methyl (2-(2-(2-(3-(4,5-dibromo-2-methyl-3,6-dioxo-3,6-dihydropyridazin-1(2*H*)-yl)propanamido)ethoxy)ethoxy)ethyl) carbamate (73 mg, 0.11 mmol, 72%) as a yellow oil. **^{1}H NMR** (600 MHz, $CDCl_3$, rotamers) δ 7.84 (br s, 0.3H), 6.38 (br s, 0.6H), 5.78 (br s, 0.3H), 5.24 (br s, 0.7H), 4.44 (t, *J* = 6.6 Hz, 2H), 4.14–4.12 (m, 2H), 3.73–3.71 (m, 3H), 3.60–3.44 (m, 12H), 2.62 (t, *J* = 6.6 Hz, 2H), 2.29–2.2 (m, 6H), 1.61–1.57 (m, 2H), 1.35–1.32 (m, 1H), 0.96–0.94 (m, 2H). **^{13}C NMR** (150 MHz, $CDCl_3$, rotamers) δ 169.1 (C), 157.0 (C), 153.1 (C), 153.0 (C), 136.6 (C), 135.5 (C), 99.0 (C), 70.4 (CH_2), 70.3 (CH_2), 69.7 (CH_2), 63.0 (CH_2), 44.6 (CH_2), 40.8 (CH_2), 39.6 (CH_2), 35.1 (CH_3), 34.1 (CH_2), 29.3 (CH_2), 29.2 (CH_2), 21.6 (CH_2), 20.2 (CH_2), 17.9 (CH), 14.3 (CH). **IR** (thin film) 3329, 2920, 2858, 1708, 1630, 1572, 1534 cm^{-1}.

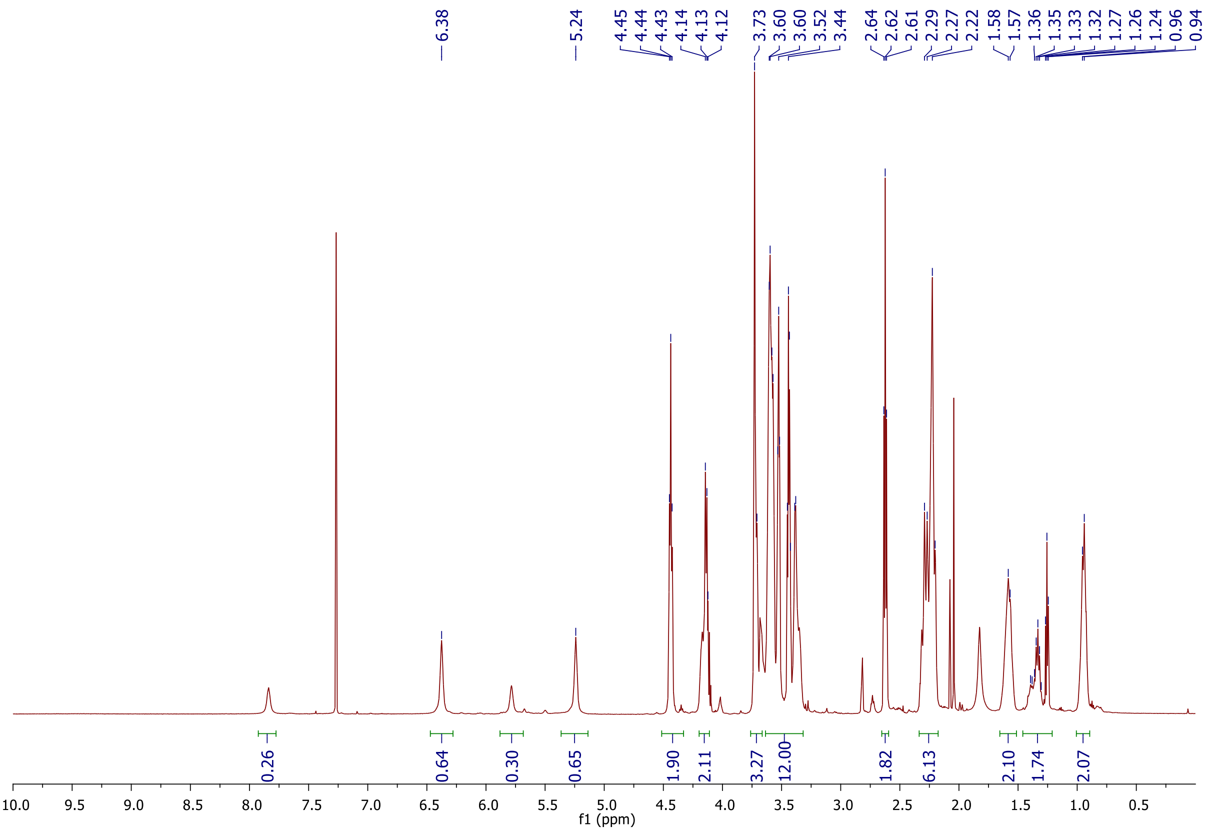

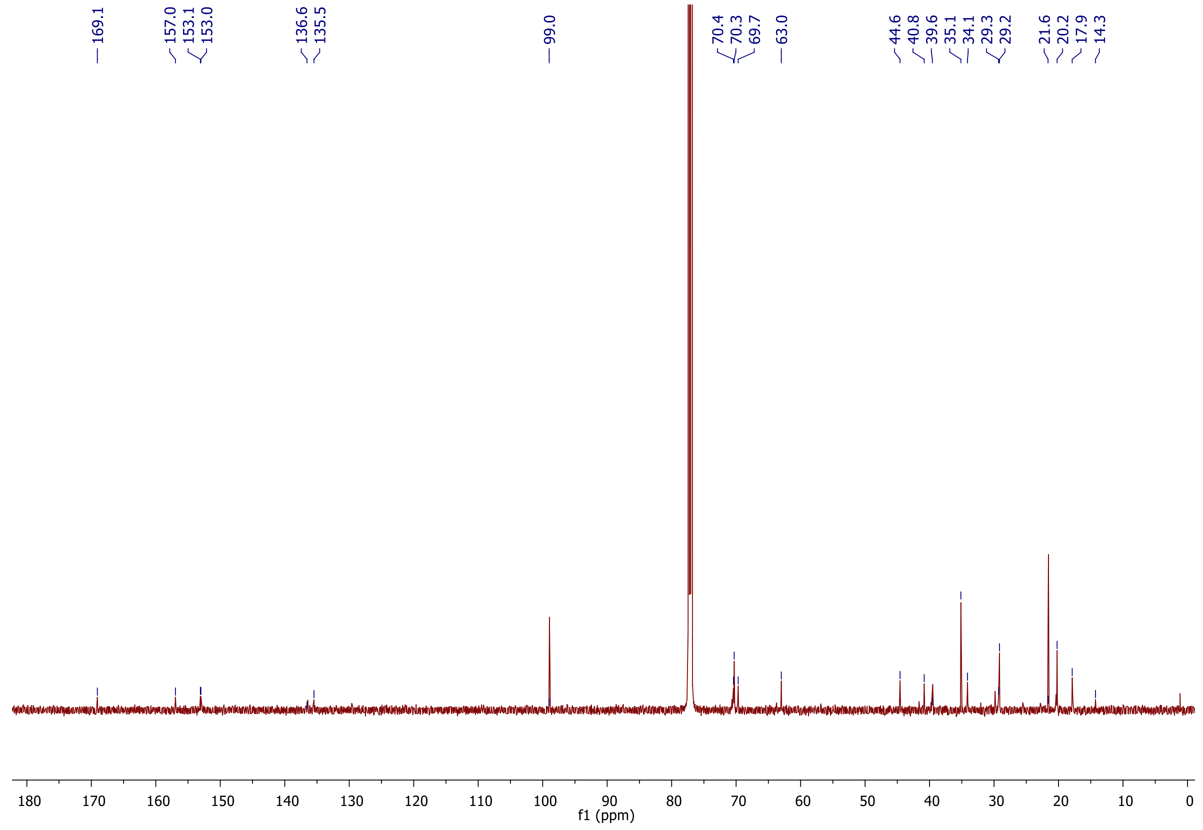

Figure S5 *^{1}H and ^{13}C NMR data for ((1R,8S,9S)-Bicyclo[6.1.0]non-4-yn-9-yl)methyl (2-(2-(2-(3-(4,5-dibromo-2-methyl-3,6-dioxo-3,6-dihydropyridazin-1(2H)-yl)propanamido)ethoxy)ethoxy)ethyl) carbamate.*

Chemical Biology

General Remarks

Reagents and solvents were purchased from commercial sources and used as supplied. All buffer solutions were prepared with double-distilled water (ddH_2O) and filter-sterilised. Borate-buffered saline (BBS) was made up of 25 mM sodium borate, 25 mM sodium chloride and 0.5 mM EDTA at pH 8.0. Phosphate buffered saline (PBS) was made up of 140 mM sodium chloride and 12 mM sodium phosphates at pH 7.4. Phosphate-buffered saline for SEC-HPLC was 140 mM NaCl, 100 mM sodium phosphates and 0.02% sodium azide at pH 7.0. Solutions of tris(2-carboxyethyl)phosphine hydrochloride (TCEP) 20 mM were prepared in ddH_2O. Filtration of particulates was carried out through Spin-X 0.22 µm cellulose acetate filters. Ultrafiltration was carried out in vivaspin 500 polyethersulfone (PES) membrane concentrators with a molecular weight cut-off (MWCO) of 10,000 Da.

Magacizumab is a humanised IgG4 full length antibody directed against hLRG1.[3] The antibody was obtained in its clinical formulation (15 mM sodium citrate, pH 6.9, 6.75% sucrose, 0.05% polysorbate 80, 104.5 mg/mL) from Abzena, dissolved in sterile water and then buffer exchanged completely for borate buffer pH 8.0 *via* ultrafiltration (MWCO 10,000 Da) prior to bioconjugation reactions. Concentration was determined by UV-vis absorbance as described.

SDS-PAGE

Non-reducing glycine-SDS-PAGE 12% acrylamide (10% for Fab) gels were performed following standard lab procedures. A 6% stacking gel was used and a broad-range molecular weight marker (10-250 kDa, Prestained PageRuler Plus Protein Standards, ThermoScientific) was run alongside the samples to estimate protein weights. Samples (10 µL at ~ 6 µM) were mixed with loading buffer (2 µL, composition for 5 × SDS: 1 g SDS, 3 mL glycerol, 6 mL 0.5 M Tri buffer pH 6.8, 2 mg bromophenol blue in 10 mL), heated at 75 °C for 5 min, and centrifuged at 16,000 RPM for 5 min. Samples were subsequently loaded into the wells in a volume of 5 µL. All gels were run at a constant current of 30 mA for 40 minutes using 1 × SDS running buffer. Gels were stained using a modified Coomassie stain (25 g ammonium sulfate, 250 mg Coomasie G-250, 8.8 mL 85% ortho-phosphoric acid, 50 mL ethanol, made up to a total of 250 mL with d.d. H_2O) at 21 °C for 16 h.

UV-vis spectroscopy

UV-Vis spectroscopy was used to determine protein concentrations and payload to antibody ratios, using a nanodrop ND-2000 spectrophotometer operating at 21 °C. Baseline correction was performed using sample buffer as a blank. Extinction coefficients for proteins (at A_{280}) and payloads (at A_{max}) are listed below. A correction factor was applied in the event that the conjugated payload had a competing absorption at A_{280}, which is listed in the table below. Pyridazinedione to antibody ratio (PAR) and fluorophore to antibody ratio (FAR) values were calculated by comparing concentrations of the payloads and the protein (calculated with corrected A_{280} values).

**Protein**	**Extinction Coefficient ε_{280} (M^{-1} cm^{-1})**
**Magacizumab**	230,690

**Payload**	**Extinction Coefficient (M^{-1} cm^{-1})**			**Correction Factor**
	ε_{280}	ε_{335}	ε_{495}	
**Pyridazinedione**	2,275	9,100	-	0.25
**AlexaFluor™ 488 azide**	8,030	-	73,000	0.11

Calculation of the molecule over antibody ratio, r, follows the formula below:

$$r = \frac{(A_\lambda)/\varepsilon_\lambda}{(A_{280} - \sum_\lambda \times CF_\lambda \times A_\lambda\ )/\varepsilon_{280}}$$

With A_λ the absorbance at wavelength λ, and ε_λ the extinction coefficient of the molecule of interest.

LCMS analysis – Method 1

LCMS was performed on small molecules using a Waters Acquity uPLC connected to Waters Acquity Single Quad Detector (SQD). All samples were diluted in deionised water and run with the following parameters. Column: Hypersil Gold C4, 1.9 µm, 2.1 µm × 50 µm. Mobile Phase: 95:5 Water (0.1% Formic Acid): MeCN (0.1% Formic Acid) Gradient over 4 min (to 5:95 Water (0.1% Formic Acid): MeCN (0.1% Formic Acid). Flow Rate: 0.6 mL/min. MS Mode: ES+. Scan Range: *m/z* = 150 – 2000. Scan time: 0.25 s. Data obtained in continuum mode. The electrospray source of the MS was operated with a capillary voltage of 3.5 kV and a cone voltage of 50 V. Nitrogen was used as the nebulizer and desolvation gas at a total flow

of 600 L/h. Ion series were generated by integration of the total ion chromatogram (TIC) over the appropriate range.

LCMS analysis – Method 2

Molecular masses of proteins were measured using an Agilent 6510 QTOF LCMS system (Agilent, UK). Agilent 1200 HPLC system was equipped with an Agilent PLRP-S, 1000A, 8 µM, 150 mm × 2.1 mm column. 10 µL of a protein sample (diluted to 0.2 mg/mL in d.d. H_2O) was separated on the column using mobile phase A (water-0.1% formic acid) and B (acetonitrile-0.1% formic acid) with an eluting gradient (as shown below) at a flow rate of 300 µL/min. The oven temperature was maintained at 60 °C. Full antibody samples (100 µL, 1.0 mg/mL, in ammonium acetate buffer, 0.2 M, pH 6.8) were deglycosylated by incubating with PNGase F (1 µL, New England BioLabs) at 37 °C for 24 h.

LCMS mobile phase gradient for A/B elution

Time (min)	Solvent A (%)	Solvent B (%)
0	85	15
2	85	15
3	68	32
4	68	32
14	65	35
18	5	95
20	5	95
22	85	15
25	85	15

Agilent 6510 QTOF mass spectrometer was operated in a positive polarity mode, coupled with an ESI ion source. The ion source parameters were set up with a VCap of 3500 V, a gas temperature at 350 °C, a dry gas flow rate at 10 L/min and a nebulizer of 30 psig. MS Tof was acquired under conditions of a fragmentor at 350 V, a skimmer at 65 V and an acquisition rate at 0.5 spectra/s in a profile mode, within a scan range between 700 and 4500 *m/z*. The data was then analysed by deconvoluting a spectrum to a zero-charge mass spectrum using a maximum entropy deconvolution algorithm within the MassHunter software version B.07.00.

Deconvoluted spectra were avoided where possible in the quantification of conjugates due to differing ionisation tendencies between species with significantly different masses.

Enzyme-linked immunosorbent assay (ELISA) against human LRG1

ELISA was used in order to determine the binding affinity of modified antibody conjugates for their antigen, human LRG1. The assay was performed as follows: a 96-well Nunc Maxisorp plate was coated overnight at 4 °C with human LRG1 (50 µL of a 4 µg/mL solution in PBS). Next, the coating solutions were removed and each well washed with 0.1% Triton ×100 in PBS (wash buffer) three times. Then, the wells were coated with a 3% BSA solution in PBS (200 µL) for 1 h at 21 °C. After this time, the wells were emptied and washed with wash buffer 3 times. Modified and non-modified antibody conjugates were diluted in PBS yielding the following concentrations (ng/mL): 280, 140, 70, 35, 17.5, 8.75, 4.38, 2.19, 1.09, 0.547, 0.273. Wells were coated with the dilution series solutions, each in triplicate, and incubated for 2 h at 21 °C. Then, the solutions were removed, and the wells washed with wash buffer 6 times. The detection antibody; anti-human IgG HRP conjugated (Sigma; 1:10,000) was added and incubated for 1 h at 21 °C. Then, the solutions were removed, and the wells washed 6 times with wash buffer. Finally, equal amounts of substrate A (stabilised hydrogen peroxide) and substrate B (stabilised tetramethylbenzidine) (ELISA substrate reagent kit; R&D Systems) were premixed and added to each well (50 µL). After *ca.* 20 min the reaction was stopped by the addition of 25 µL of 2 N sulfuric acid. Absorbance was measured at 450 nm. PBS-only controls were included in each experiment. Each sample was tested in triplicate, and errors are shown as the standard deviation of the average.

Alexa Fluor™ 488 conjugate serum stability [4]

Alexa Fluor™ 488 antibody–fluorophore conjugate (AFC) **3a** was prepared as a 0.2 mg/mL solution in PBS 140 mM sodium chloride, 12 mM sodium phosphates and 2 mM sodium azide at pH 7.4. The conjugate was diluted with 50% of human blood serum to give a final concentration of 0.1 mg/mL of AFC and 1 mM sodium azide. A single aliquot (50 µL) was taken, flash frozen and stored at -80 °C. The remaining solution was incubated at 37 °C under shaking (300 rpm) and under the cover of light. Aliquots (50 µL) were taken at 1, 2, 4 and 7 days, flash frozen and stored at -80 °C. Aliquots were thawed, spin-filtered (0.22 µm filter) and diluted 100× with elution buffer. Samples (20 µL) of diluted aliquots were analysed by SEC-

HPLC on a TSK gel G3000SWXL (7.8 mm x 30 cm) column connected to an Agilent 1200 HPLC system equipped with a 1200 series diode array detector and a fluorescence detector. Samples were eluted using PBS 140 mM NaCl, 100 mM sodium phosphates and 0.02% sodium azide at pH 7.0 as mobile phase at a flow rate of 0.5 mL/min. over 30 min. Fluorescence was detected with an excitation wavelength of 495 nm and emission wavelength of 525 nm.

Ellman’s test

A 1 mM solution of 5,5’-dithio-bis-(2-nitrobenzoic acid) (Ellman’s reagent) was prepared in PBS. The assay was performed by mixing 5 µL of this solution with 5 µL of reduced Magacizumab at 20 µM and diluting with PBS (60 µL). The solution was incubated at 20 °C for 2 min before measuring absorption at 280 nm (protein concentration) and 412 nm (2-nitro-5-thiobenzoic acid). For baseline correction, a sample of 5 µL of the 1 mM Ellman’s reagent diluted with 65 µL PBS was used as a blank. The sulfhydryl per antibody ratio (SAR) was calculated as follows with ε_{412} = 14150 M^{-1} cm^{-1} for 2-nitro-5-thiobenzoic acid and ε_{280} = 210000 M^{-1} cm^{-1} for Magacizumab, where A_{280} and A_{412} define the absorption measured at 280 nm in the absence of Ellman’s reagent and at 412 nm in the presence of the reagent respectively.

$$\mathrm{SAR} = \frac{A412/\varepsilon 412}{A280/\varepsilon 280}$$

Conjugation methods

Reduction of Magacizumab with TCEP

In order to avoid the use of excess reducing agent, reduction optimisation studies were carried out with Magacizumab. The four solvent accessible disulfide bonds of the antibody were reduced using mild reducing agent TCEP. To a solution of Magacizumab (20 µM, 50 µL, 0.01 µmol) in borate buffer (BBS; 25 mM sodium borate, 25 mM NaCl, 0.5 mM EDTA, pH 8.0) was added TCEP (20 mM in dH_2O, 10-150 eq.), and the reaction mixture incubated for 4 h at 37 °C under mild agitation (300 rpm). After this time excess TCEP was removed by diafiltration into EDTA-containing BBS to inhibit re-oxidation of the reduced disulfides. Confirmation of reduction was achieved by SDS- PAGE and by mass spectrometry. For the purposes of analysis by mass spectrometry, excess *N*-methylmaleimide was added to the reduced samples to cap the liberated thiols and prevent oxidation of the reduced samples. Deglycosylation of samples was achieved by incubation with the enzyme PNGase F at 37 °C for 24 h prior to analysis by mass spectrometry.

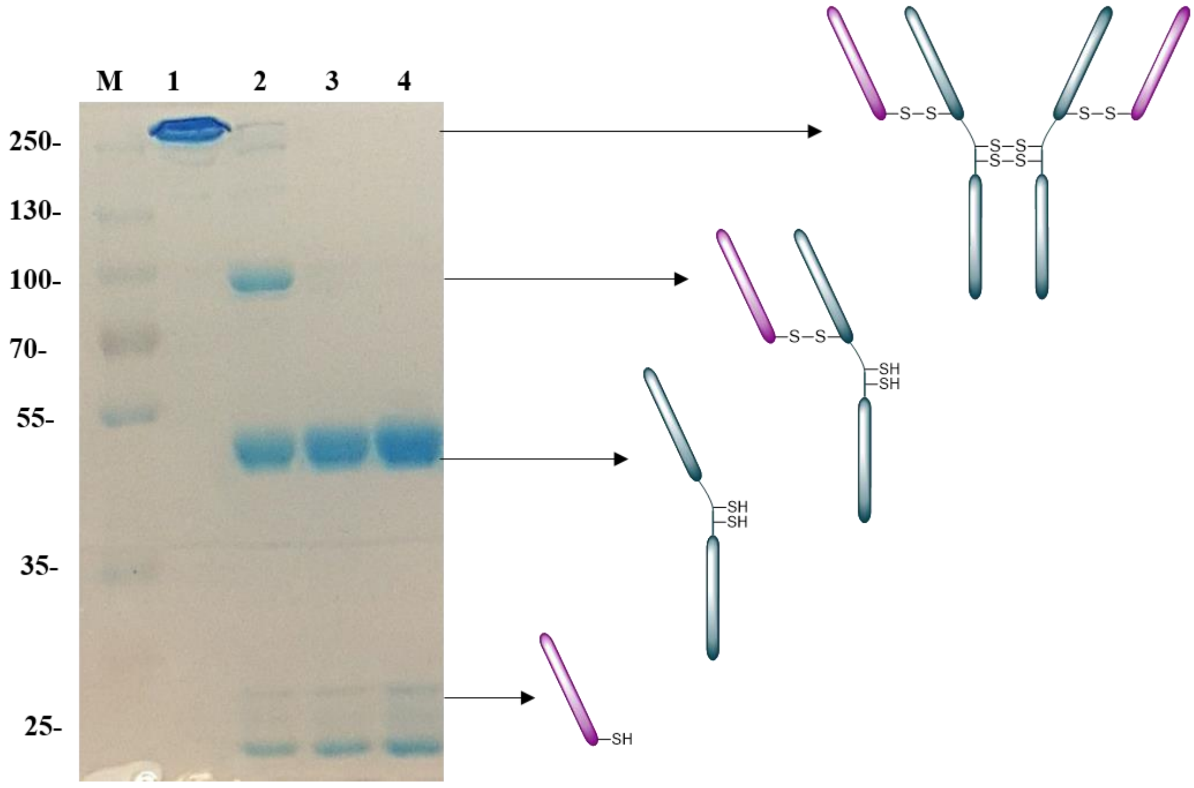

Figure S6 *SDS-PAGE analysis of Magacizumab reduction by TCEP in borate buffer pH 8.0 at 37 °C for 4 h. M = molecular weight marker; Lane 1 – native Magacizumab; 2 – 50 eq. of TCEP; 3 – 100 eq. of TCEP; 4 – 150 eq. of TCEP.*

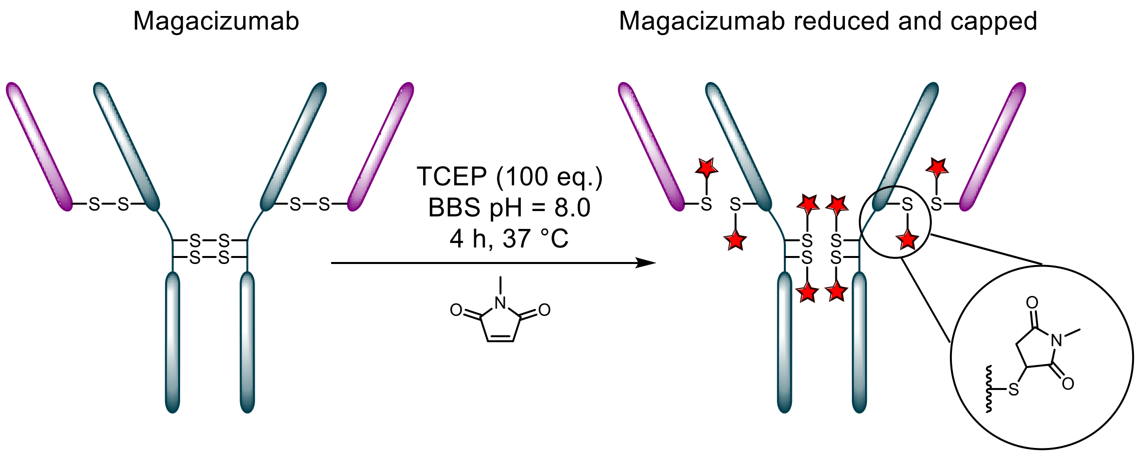

Figure S7 *Reagents and conditions for Magacizumab reduction: 100 eq. TCEP for 4 h, then thiol capping with maleimide, BBS pH 8.0, 37 °C.*

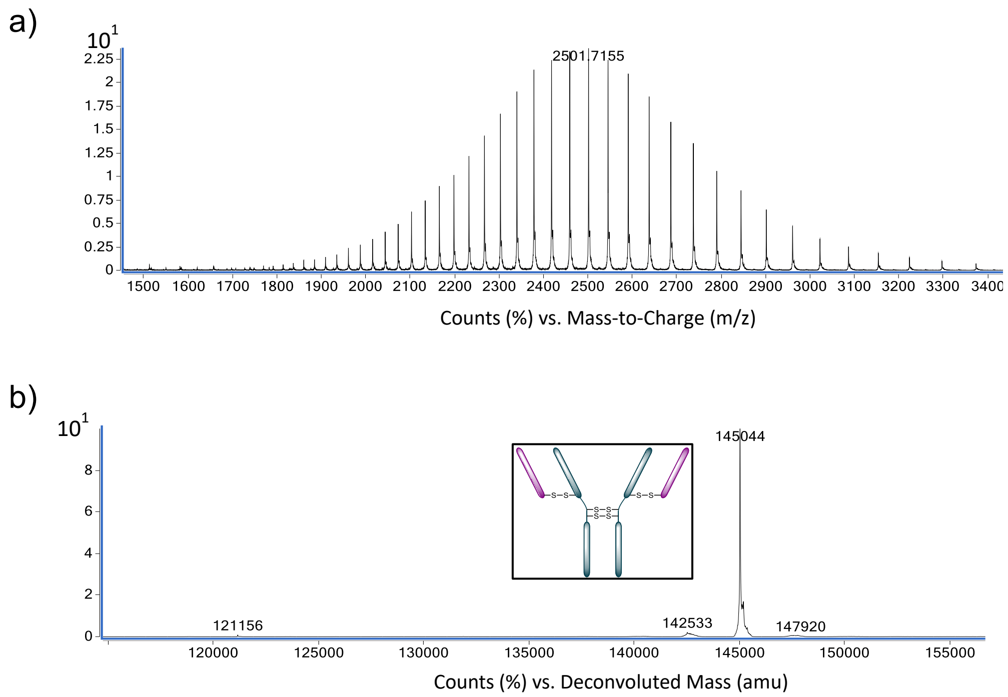

Figure S8 *a) Non-deconvoluted and b) de-convoluted LCMS data for native Magacizumab (deglycosylated).*

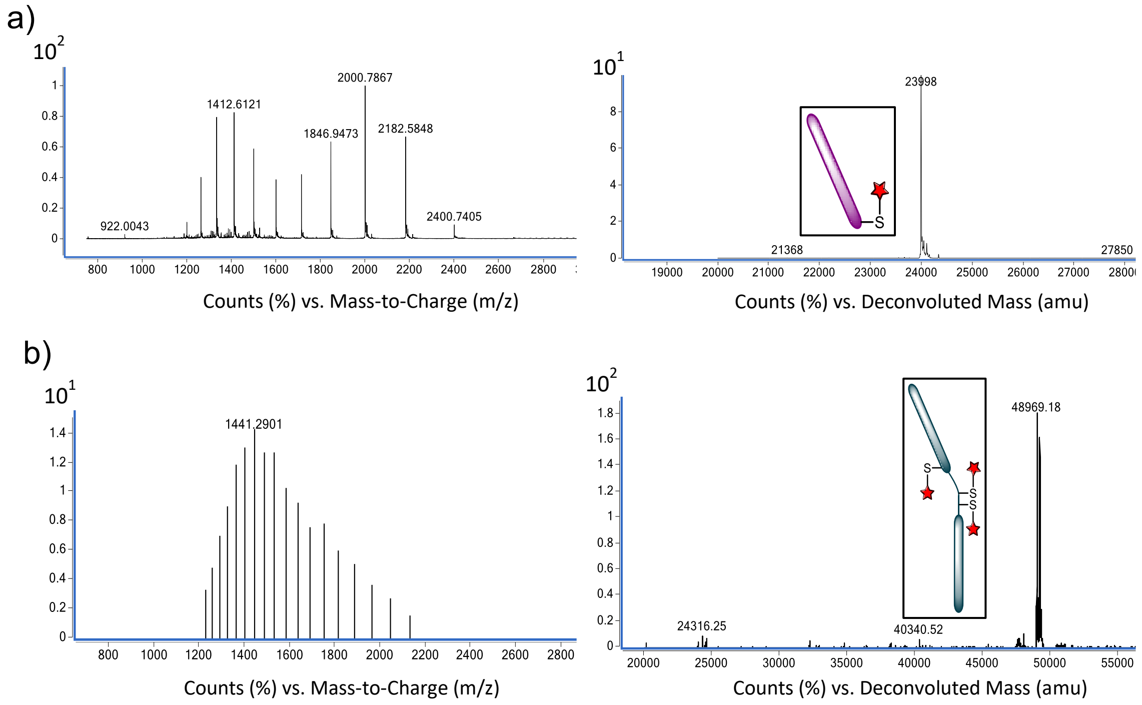

Figure S9 *Non-deconvoluted and deconvoluted LCMS data for Magacizumab reduced with 100 eq. 20 mM TCEP and capped with maleimide (deglycosylated) displaying a) light chain and b) heavy chain fragments.*

Step-wise reduction of Magacizumab and reaction with PD 1 at 21 °C to form conjugate 3a

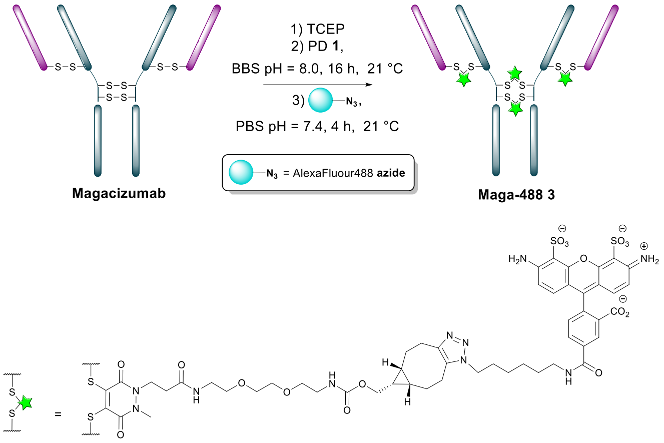

To a solution of Magacizumab (20 µM, 100 µL) in borate buffer (BBS; 25 mM sodium borate, 25 mM NaCl, 0.5 mM EDTA, pH 8.0) was added TCEP (10 µL, 20 mM in ddH_2O, 100 eq.), and the reaction mixture incubated for 4 h at 37 °C under mild agitation (300 rpm). After this time excess TCEP was removed by diafiltration into fresh BBS buffer using PD Minitrap G-25 columns (GE Healthcare) and the concentration was corrected to 20 µM. PD **1** (2 µL, 20 mM in DMSO, 20 eq.) was added to the solution of reduced Magacizumab at 21 °C and the solution incubated for 16 h. Excess reagents were removed by repeated diafiltration into fresh PBS buffer (140 mM sodium chloride, 1 mM potassium phosphate monobasic and 8 mM sodium phosphate dibasic at pH 7.4) using diafiltration columns (GE Healthcare, 10,000 Da MWCO), and the volume was corrected to 100 µL. The samples were analysed by SDS-PAGE and UV-Vis spectroscopy, which was used to determine a PAR of 3.79. Following this, Alexa Fluor^{TM} 488 Azide (Molecular Probes) (1 µL, 20 mM in DMSO, 10 eq.) was added and the reaction mixture was incubated at 21 °C for 4 h. Excess fluorophore was removed by repeated diafiltration into fresh PBS using diafiltration columns (GE Healthcare, 10,000 Da MWCO). Following this, sample analysis by SDS-PAGE and UV-vis spectroscopy revealed conversion to the desired antibody-PD Alexa Fluor^{TM} 488 Azide bioconjugate **3a** with a fluorophore to antibody ratio (FAR) of 3.80. Expected mass by LCMS (method 2): 149,692 Da. Observed mass by LCMS (method 2): 149,684 Da.

A TCEP reduction control was also performed on Magacizumab: TCEP (5 µL, 20 mM in ddH_2O, 100 eq.) was added to Magacizumab (20 µM, 50 µL, 0.02 µmol) in BBS and the reaction mixture was incubated at 37 °C for 4 h.

Compound	Molecular weight (Da)
PD **1**	662
Alexa Fluor ™ 488 azide	656
PD-Alexa Fluor™ 488	1322

LCMS (method 1) data for click reaction of PD **1** with N_3-PEG_3-vc-PAB-MMAE, including controls:

LCMS data of PD **1**:

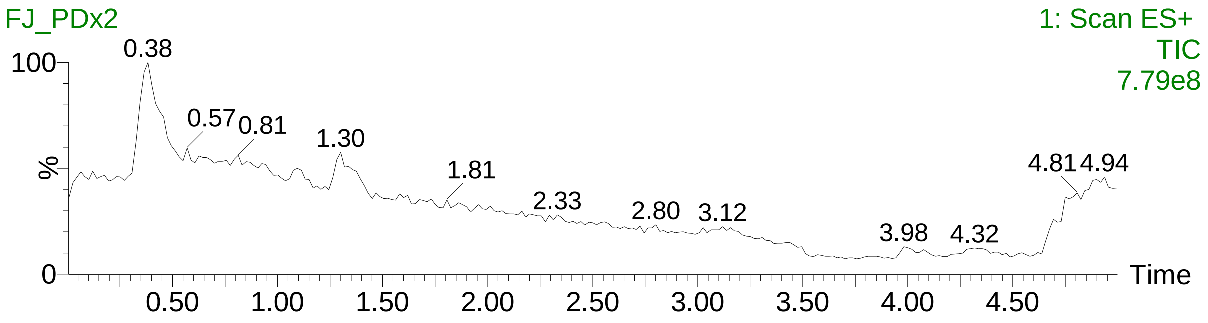

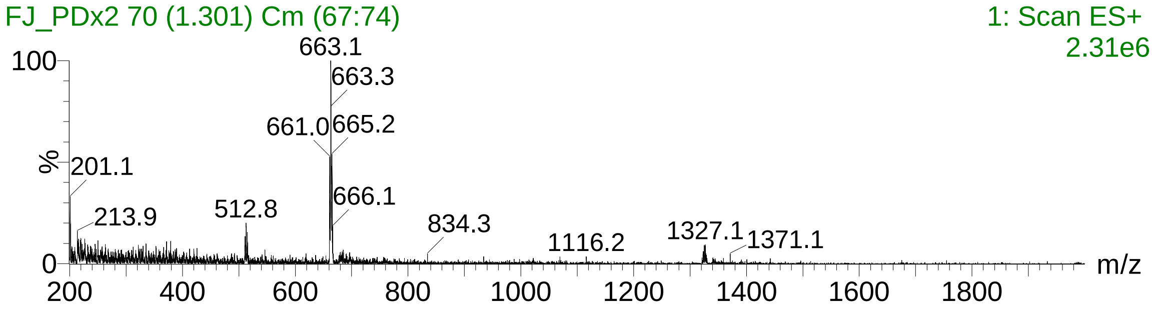

LCMS data of Alexa Fluor ™ 488 azide:

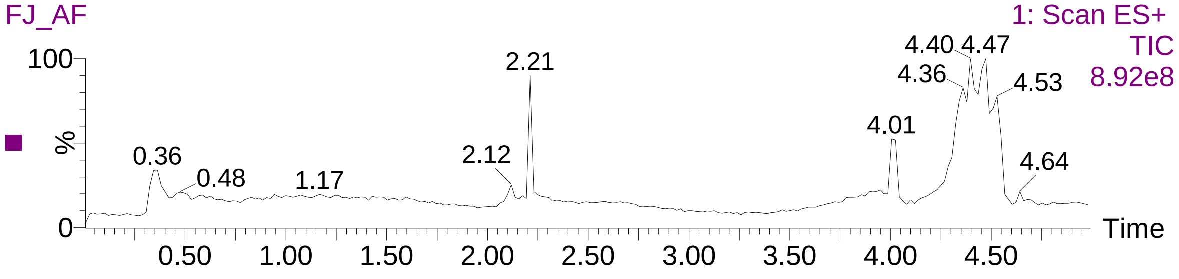

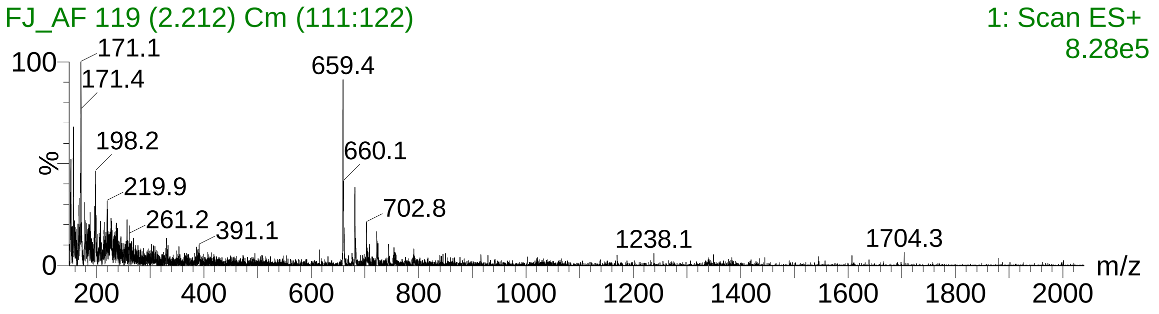

LCMS data of reaction of PD **1** with Alexa Fluor ™ 488 azide showing formation of clicked product and complete consumption of PD **1**:

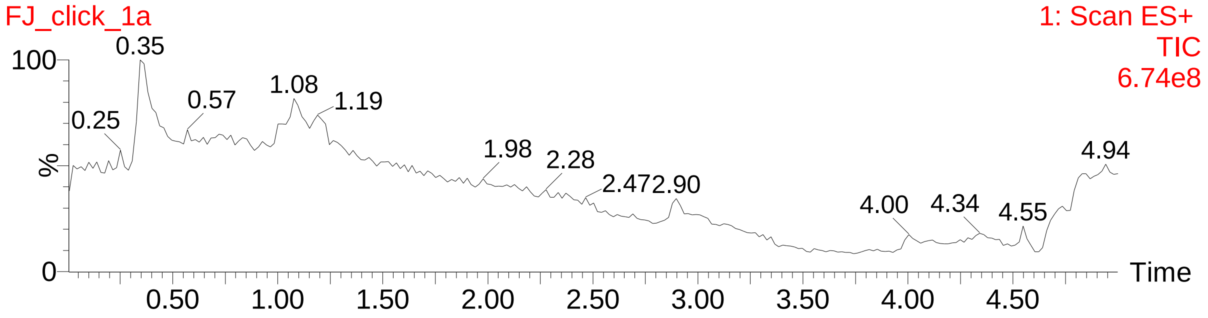

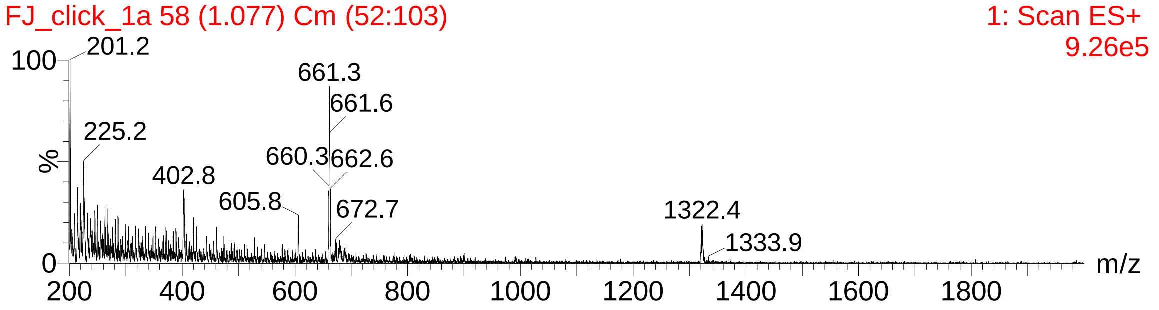

LCMS (method 2) data for Maga-488 **3a**:

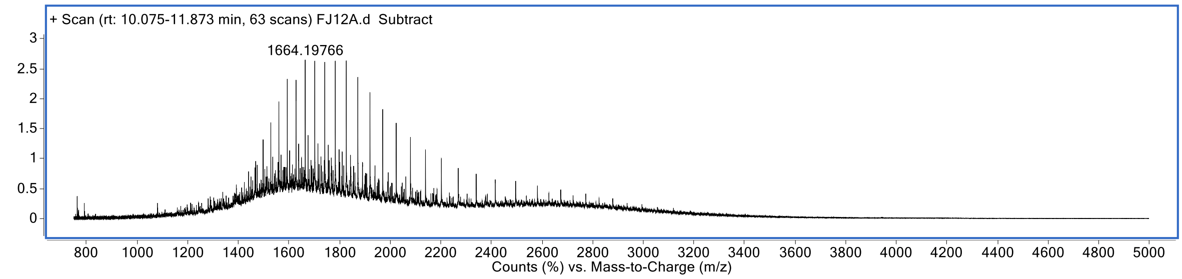

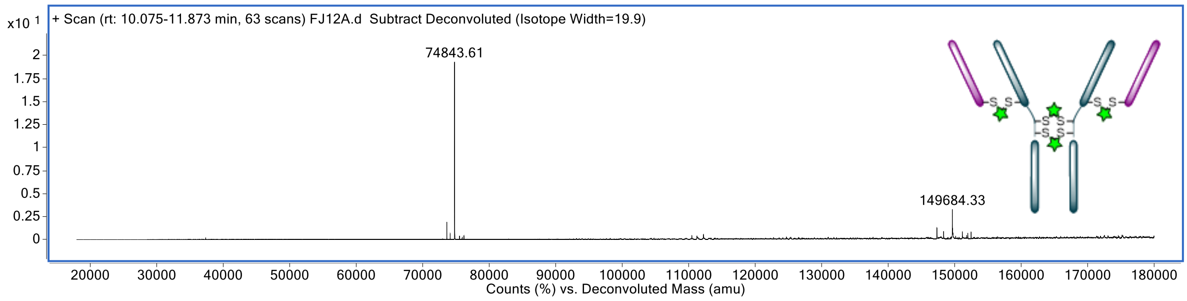

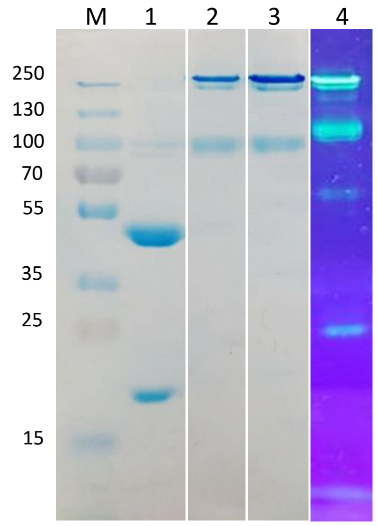

M – Marker

1 – Magacizumab, reduced

2 – Magacizumab, rebridged

3 - Magacizumab; rebridged and clicked

4 - Magacizumab; rebridged and clicked

Figure S10 *SDS-PAGE analysis of conjugate* ***3a*** *(at 21 °C); Lane M – Molecular weight marker; Lane 1 – Magacizumab reduced with 100 eq. 20 mM TCEP; Lane 2 – PD-rebridged Magacizumab* ***2a****; Lane 3,4 – Maga-AF488 conjugate (***conjugate 3a****).*

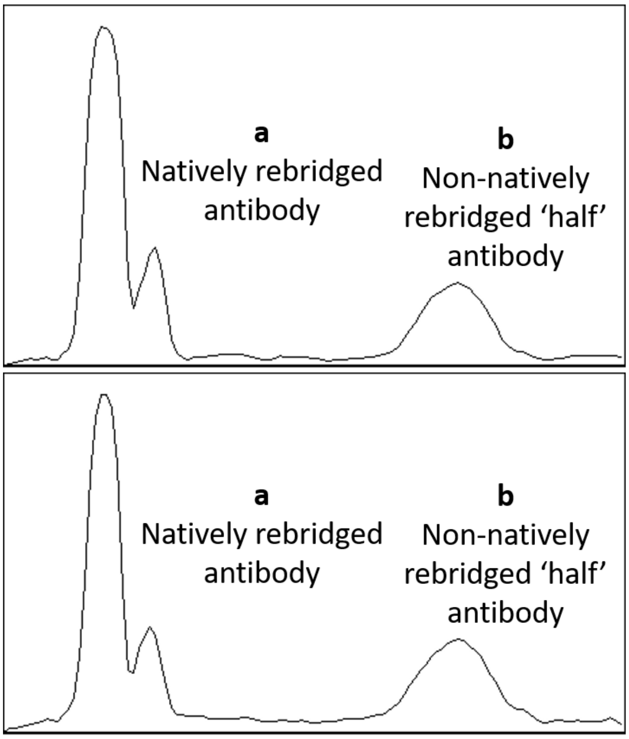

Figure S11 *Densitometry traces showing a) natively rebridged and b) non-natively rebridged portions of antibody–fluorophore conjugates (AFCs)* ***3a*** *and* ***3b*** *prepared at 21°C (top) and 4 °C (bottom), respectively.*

Conjugation performed at 21 °C	
**Species**	**Abundance (%)**
a	69
b	31

Table S1 *Quantification of a) natively rebridged and b) non-natively rebridged portions of antibody–fluorophore conjugate (AFC)* ***3a*** *prepared at 21 °C by densitometry analysis.*

Conjugation performed at 4 °C	
**Species**	**Abundance (%)**
a	74
b	26

Table S2 *Quantification of a) natively rebridged and b) non-natively rebridged portions of antibody–fluorophore conjugate (AFC)* ***3b*** *prepared at 4 °C by densitometry analysis.*

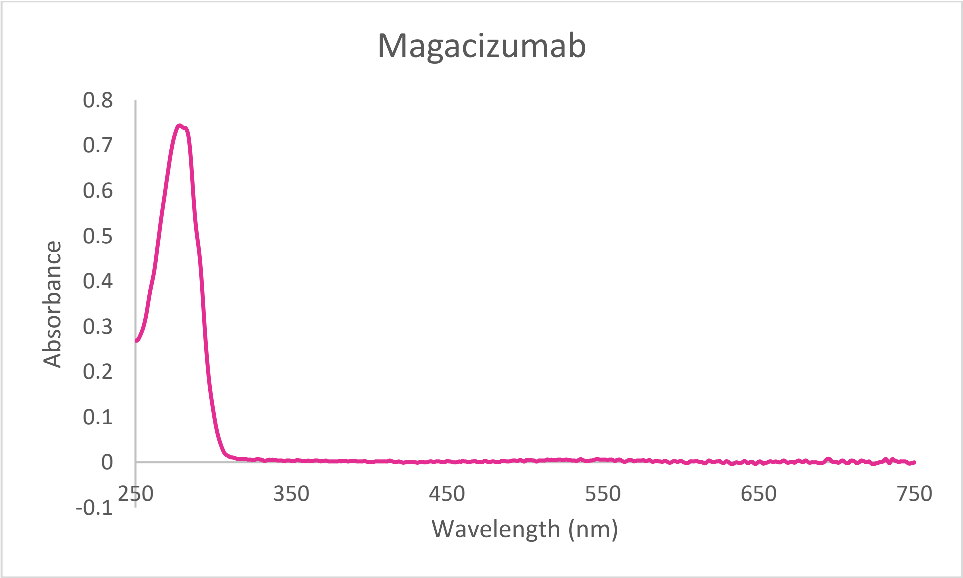

Figure S12 *UV-Vis data for Magacizumab*

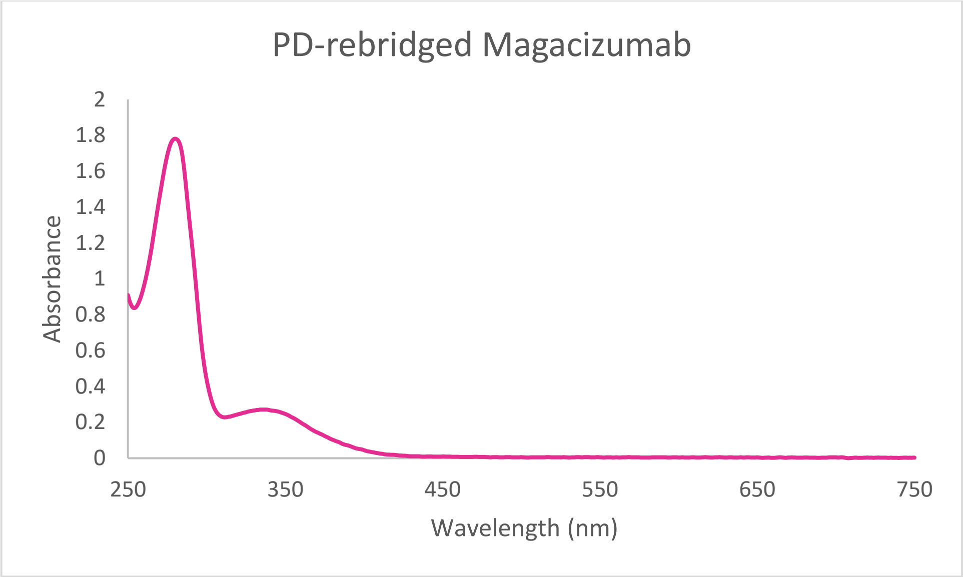

Figure S13 *UV-Vis data for PD-rebridged Magacizumab* ***2a****.*

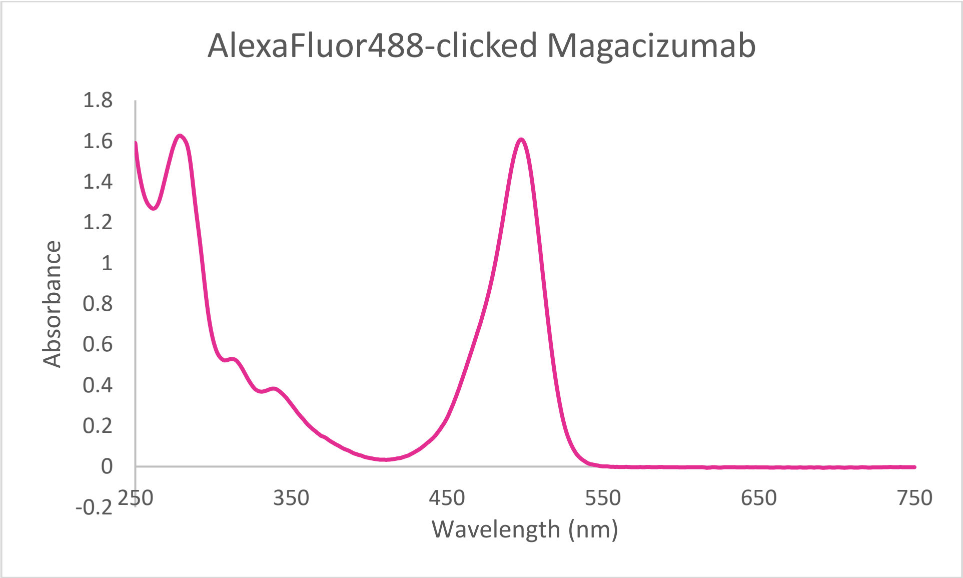

Figure S14 *UV-Vis data for Alexa Fluor* ^{TM} *488-clicked Magacizumab* ***3a****.*

Preparation of PD-MMAE

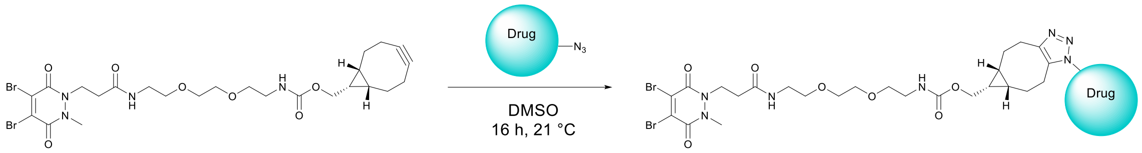

1 **4**

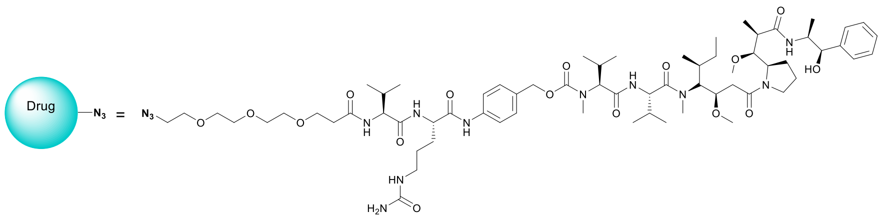

To a solution of PD **1** (4 µL, 20 mM in DMSO, 1.08 mol) was added N_3-PEG$_3$-vc-PABC-MMAE (120 µL, 10 mM in DMSO, 1.20 mol). The reaction mixture was left to react at 21 °C overnight. The product was analysed by LCMS to reveal the formation of PD **4** with complete consumption of limiting reagent PD **1**. Whilst excess unreacted N_3-PEG$_3$-vc-PABC-MMAE was observed by LCMS, this species is likely to be unreactive towards proteins, and so further purification was not conducted prior to conjugation to Magacizumab.

Compound	Molecular weight (Da)
PD **1**	662
N_3-PEG$_3$-vc-PABC-MMAE	1353
PD **4**	2015

LCMS (method 1) data for click reaction of PD **1** with N_3-PEG$_3$-vc-PAB-MMAE, including controls:

LCMS data of PD **1**:

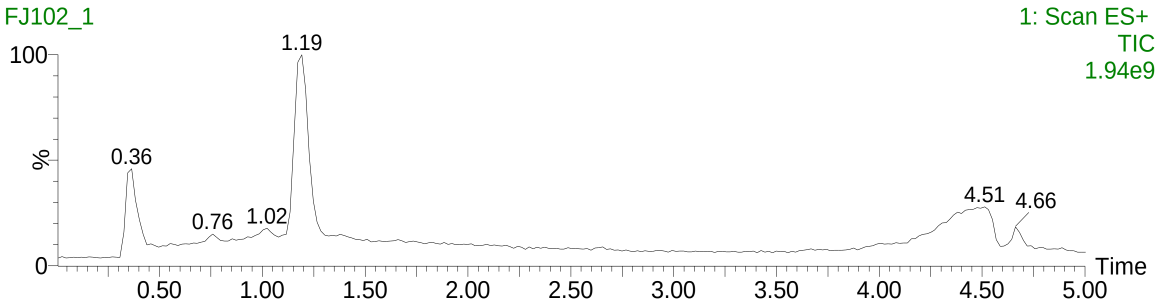

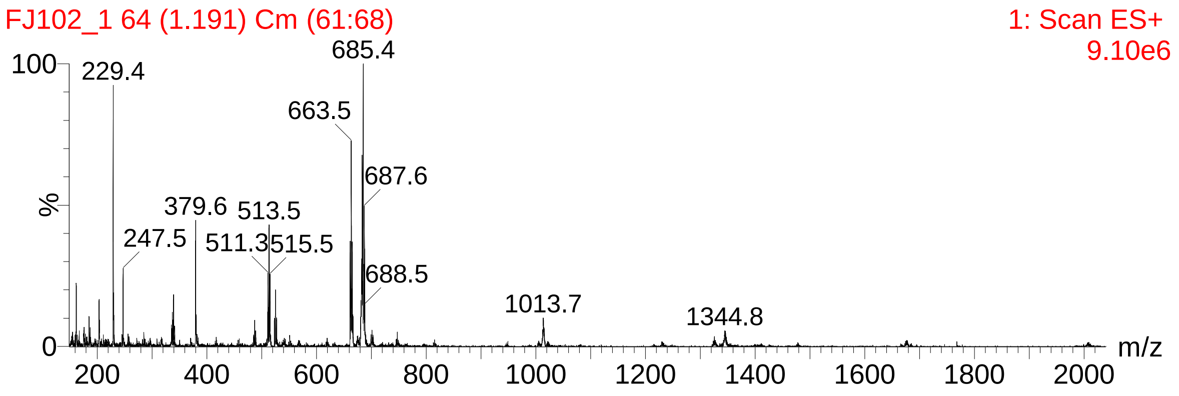

LCMS data of N_3-PEG$_3$-vc-PAB-MMAE:

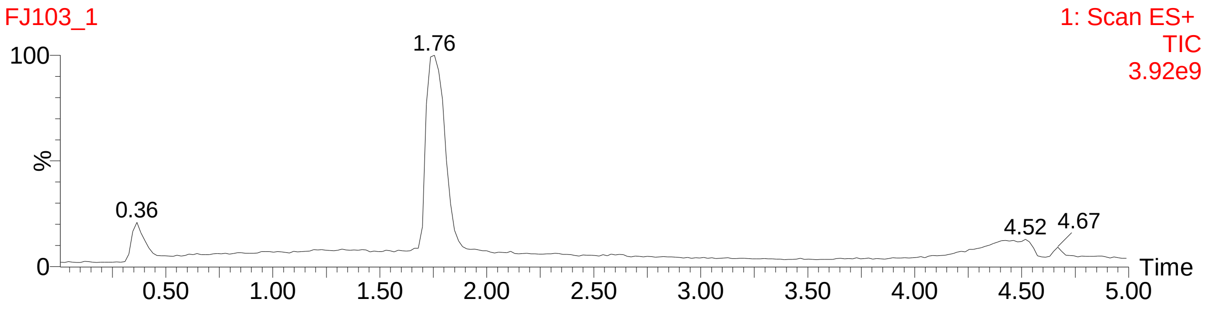

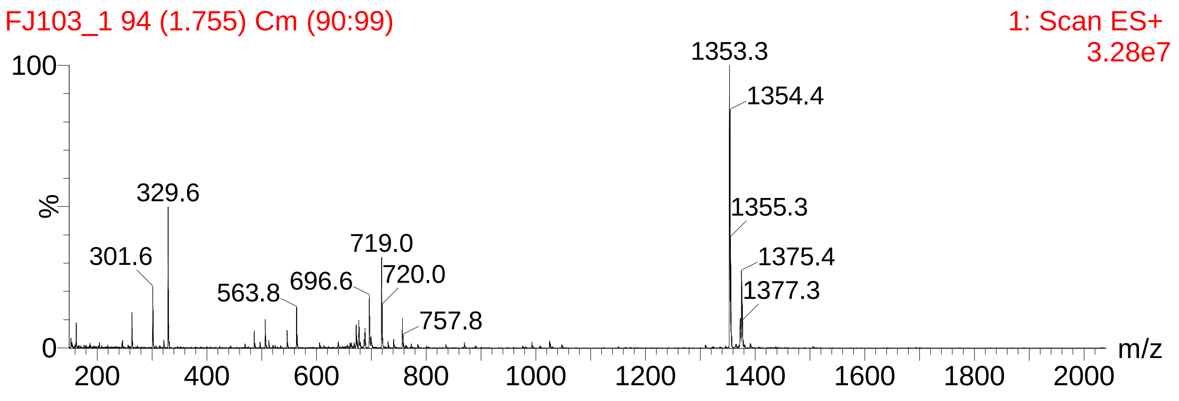

LCMS data of reaction of PD **1** with N_3-PEG_3-vc-PAB-MMAE showing formation of PD **4** and complete consumption of PD **1**:

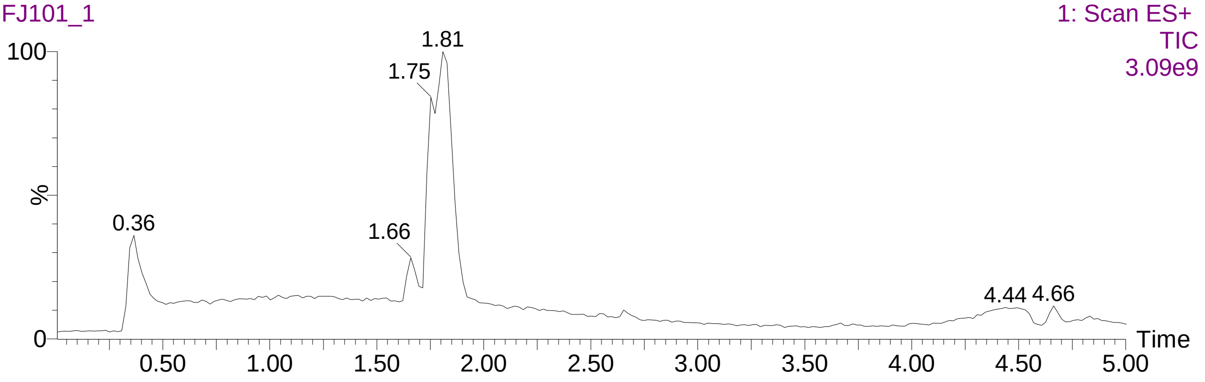

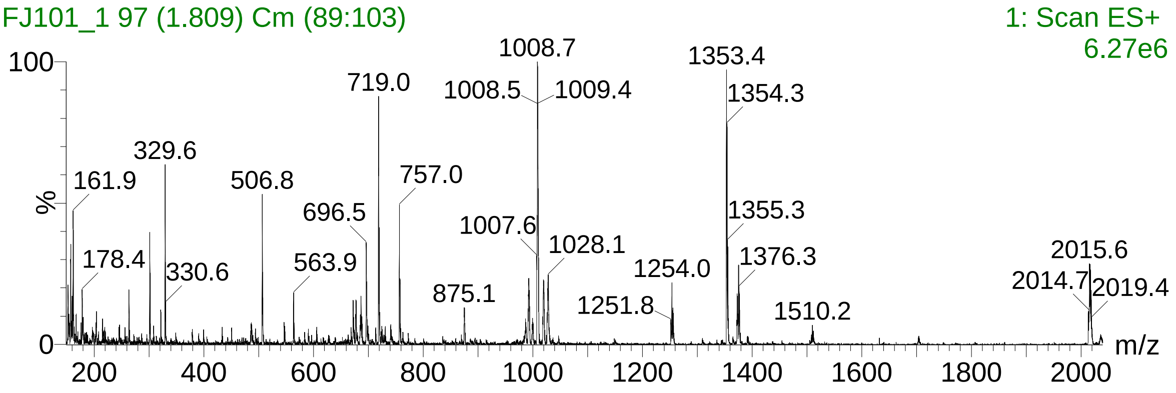

Protocol comparison for the conjugation of Magacizumab with PD-MMAE 4 to form ADC 5

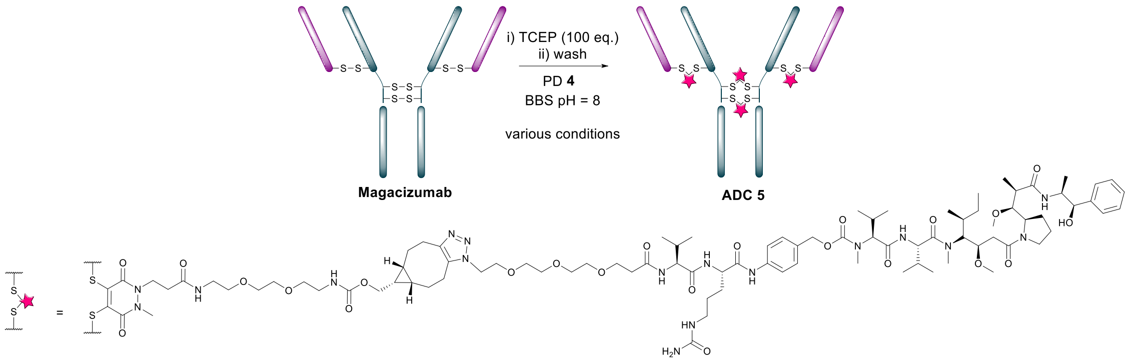

Reaction conditions are in Table S1

To a solution of Magacizumab (20 µM, 100 µL, 0.02 µmol) in BBS pH 8.0 was added TCEP (20 mM, 10 µL, 100 eq.) and the reaction incubated at 37 °C for 4 h under mild agitation (300 rpm). After this, excess TCEP was removed by ultrafiltration (10,000 Da MWCO) and the reduced Magacizumab concurrently transferred into fresh BBS pH 8.0. Following this, PD-MMAE **4** (6.25 mM, 10 eq. or 20 eq., in DMSO) was immediately added to the reduced Magacizumab solution, thus giving a solution with a final DMSO content of 6% or 8% (v/v). The reaction was incubated for 16 h at 21 °C or 37 °C. After this, excess reagents were removed by ultrafiltration (10,000 Da MWCO) into PBS pH 7.4 to afford the modified Magacizumab PD-MMAE ADC **5** in PBS at a final concentration of 20 µM. Average PAR by UV-Vis is reported in Table S1.

Reaction #	Temperature (°C)	PD equivalents	Final DMSO%	PAR
**1**	21	20	6%	3.60
**2**	37	20	6%	3.77
**3**	21	20	8%	3.63
**4**	37	20	8%	3.89
**5**	21	10	6%	3.61
**6**	37	10	6%	3.72

***Table S3** Reaction conditions for conjugation of PD-MMAE **4** to Magacizumab to form Magacizumab PD-MMAE ADC **5**. PAR represents PD (and hence drug) loading on the antibody.*

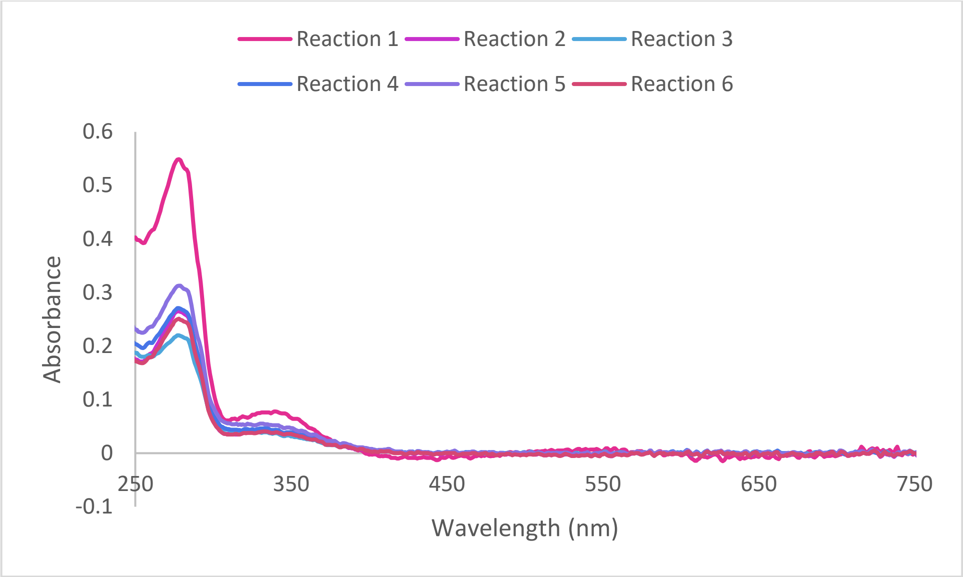

Figure S15 *UV-Vis analysis of ADC products prepared according to Table S1. The UV-Vis trace was used to calculate PD (drug) loading as reported in Table S1.*

AlexaFluor 'Click' control experiment:

An Alexa Fluor ™ 488 click control was performed on the Magacizumab PD-MMAE ADC **5** (reaction 6) to ascertain whether all clickable handles on the functionally rebridged ADC had reacted with MMAE-azide: Alexa Fluor ™ 488 (10 mM, 1 µL, 20 eq.) was added to Magacizumab PD-MMAE ADC **5** (20 µM, 50 µL) in PBS. Sample analysis by and UV-vis spectroscopy gave an FAR of 0.2.

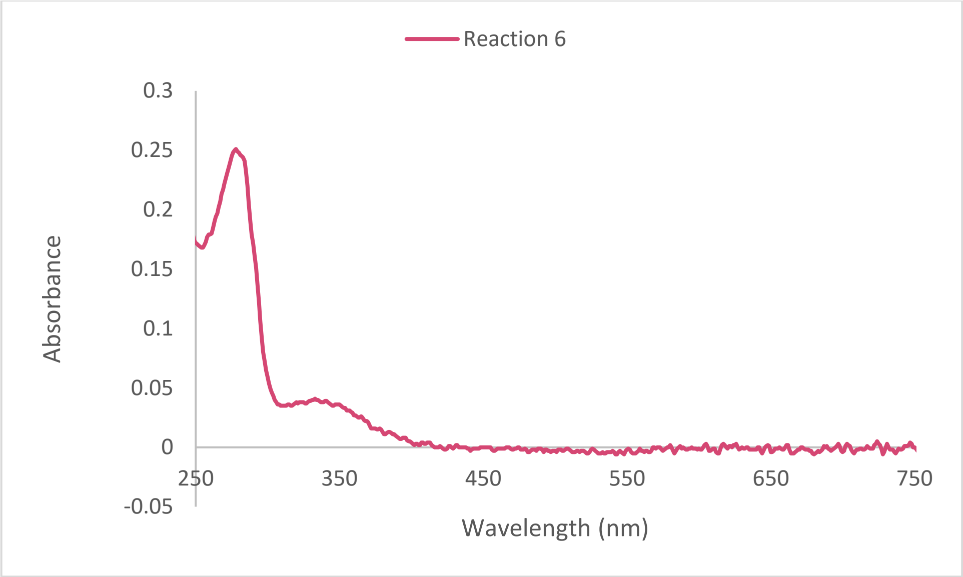

__Figure S16__ UV-Vis analysis of ADC (reaction 6) after incubation with Alexa Fluor™ 488 azide (control experiment). The UV-Vis trace was used to calculate PD and fluorophore loading (PAR = 3.72, FAR = 0.2).

Reaction of Magacizumab with a PD lacking a strained alkyne clickable handle (dibromo diethyl pyridazinedione 6), followed by incubation with azide bearing Alexa Fluor™ 488 azide

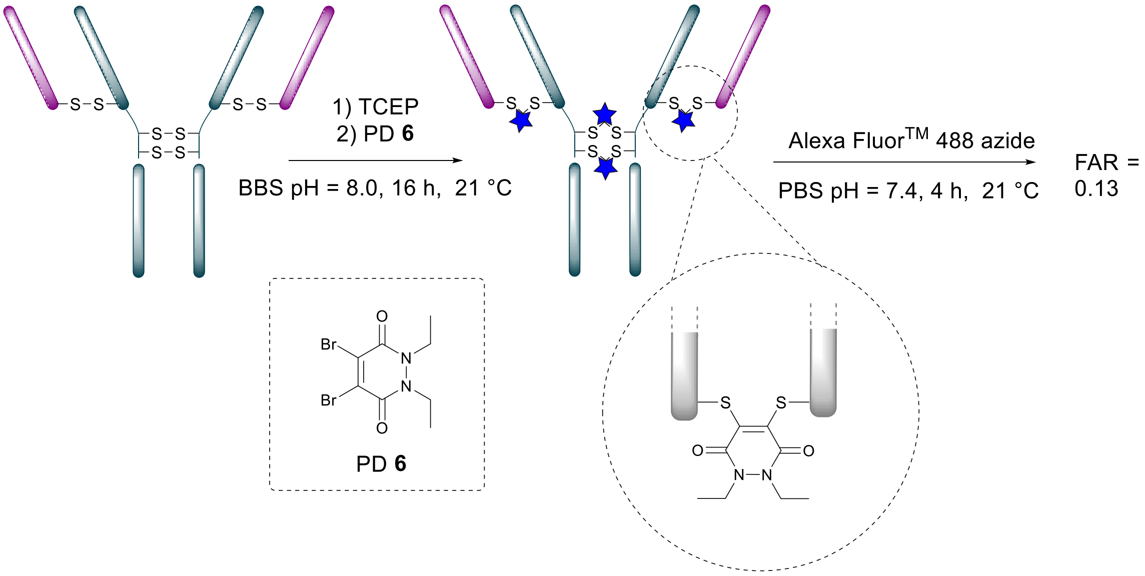

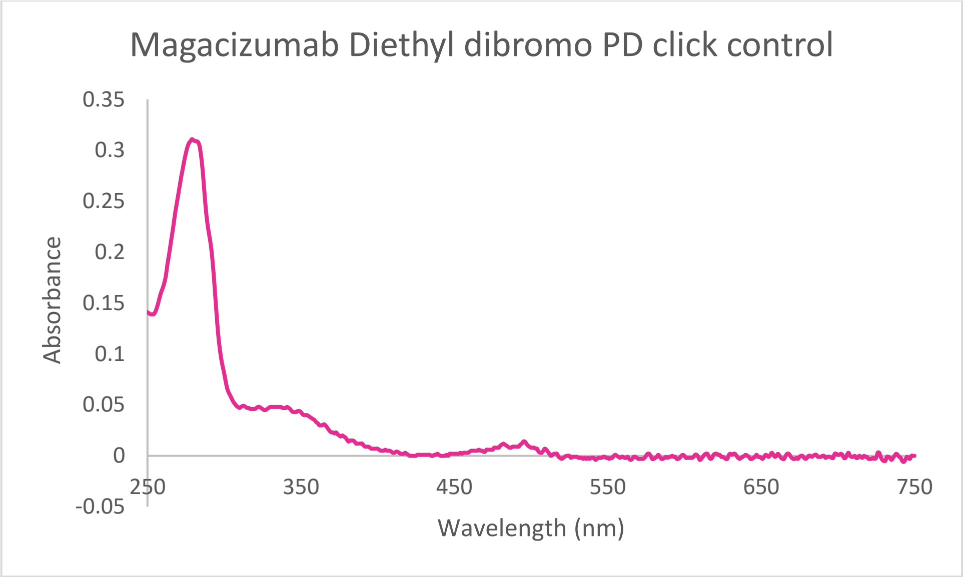

Figure S17 *UV-Vis data for reaction of Magacizumab with dibromo diethyl pyridazinedione* **6**, *bearing no 'clickable' handles, followed by incubation with Alexa Fluor ™ 488 azide. FAR = 0.13*

Conjugation of Magacizumab with PD-MMAE 4 to form ADC 5

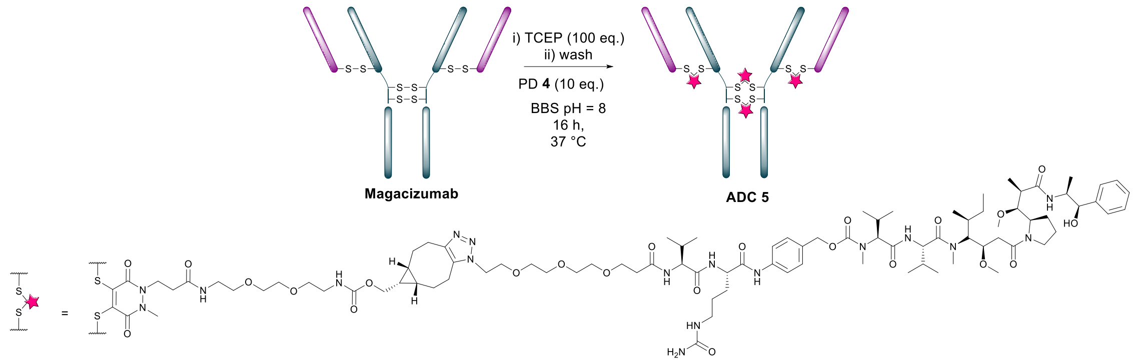

To a solution of Magacizumab (20 µM, 3.3 mL) in BBS pH 8.0 was added TCEP (330 µL, 20 mM in ddH_2O, 100 eq.) and the reaction incubated at 37 °C for 4 h under mild agitation (300 rpm). After this, excess TCEP was removed by ultrafiltration (10,000 Da MWCO) and the reduced Magacizumab concurrently transferred into fresh BBS pH 8.0. Following this, PD-MMAE **4** (6.25 mM, 106 µL, 10 eq., in DMSO) was immediately added to the reduced Magacizumab solution, thus giving a solution with a final DMSO content of 6% (v/v). The reaction was incubated at 37 °C for 16 h. After this, excess reagents were removed by ultrafiltration (10,000 Da MWCO) into PBS pH 7.4 to afford the modified Magacizumab PD-MMAE ADC **5** in PBS at a final concentration of 20 µM. Yield 90%, average PDAR by UV-Vis was 4.0. A TCEP reduction control was also performed on Magacizumab PD-MMAE ADC **5** to demonstrate that all solvent accessible disulfide bonds were functionally rebridged: TCEP (5 µL, 20 mM in ddH_2O, 100 eq.) was added to Magacizumab PD-MMAE ADC **5** (20 µM, 50 µL) in BBS.

Additionally, an Alexa Fluor ™ 488 click control was also performed on Magacizumab PD-MMAE ADC **5** to demonstrate that that all clickable handles on the functionally rebridged ADC had reacted with MMAE-azide: Alexa Fluor 488 (1 µL, 10 mM in DMSO, 20 eq.) was added to Magacizumab PD-MMAE ADC **5** (20 µM, 50 µL) in PBS and the reaction mixture was incubated at 21 °C for 4 h. Excess fluorophore was removed by repeated diafiltration into fresh PBS using diafiltration columns (GE Healthcare, 10,000 Da MWCO).

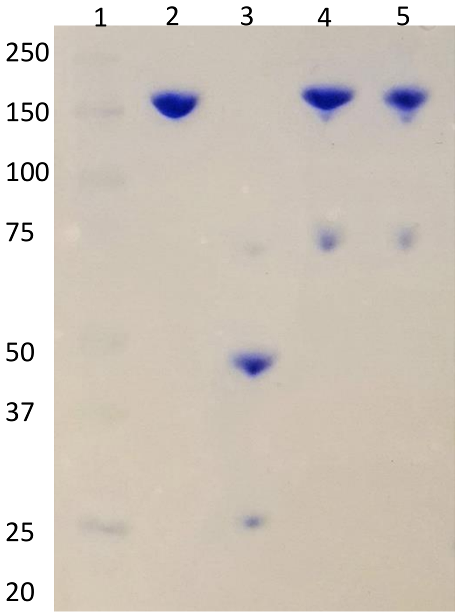

1 Ladder
2 Magacizumab, native
3 Magacizumab, reduced
4 ADC **5**
5 ADC **5**, reduced

Note: reduced with TCEP (100 eq.)

Figure S18 *SDS-PAGE analysis of ADC* ***5****; Lane 1 – Molecular weight marker; Lane 2 – Native unmodified Magacizumab; Lane 3 – Magacizumab reduced with 100 eq. 20 mM TCEP; Lane 4 – ADC* ***5****; Lane 5 – ADC* ***5*** *reduced with 100 eq. 20 mM TCEP.*

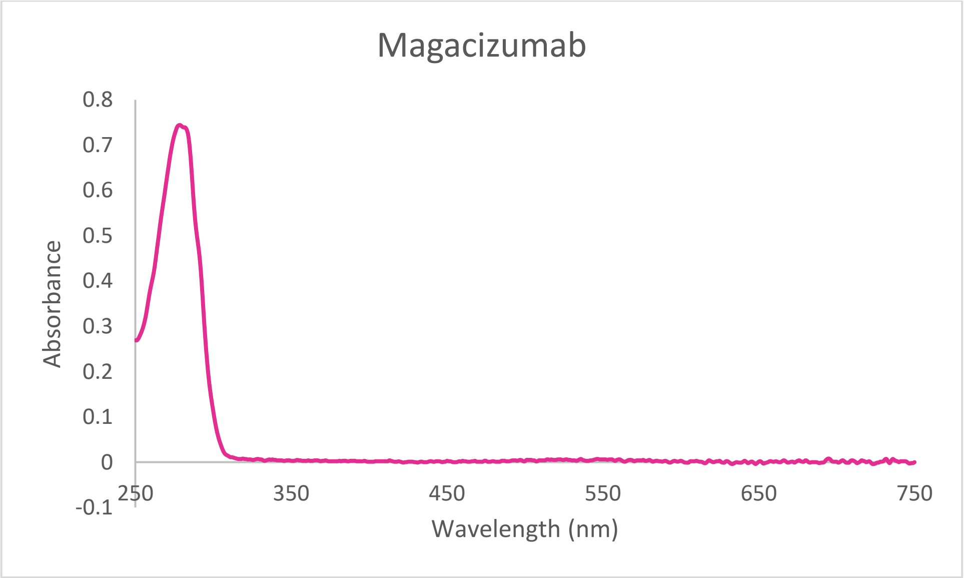

Figure S19 *UV-Vis data for Magacizumab*

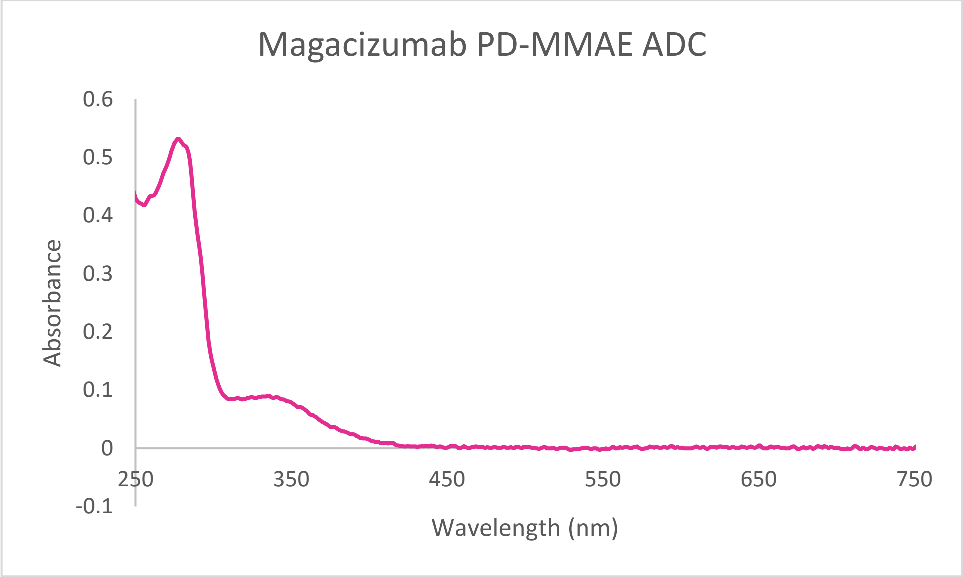

Figure S20 *UV-Vis data for Magacizumab PD-MMAE ADC* ***5***

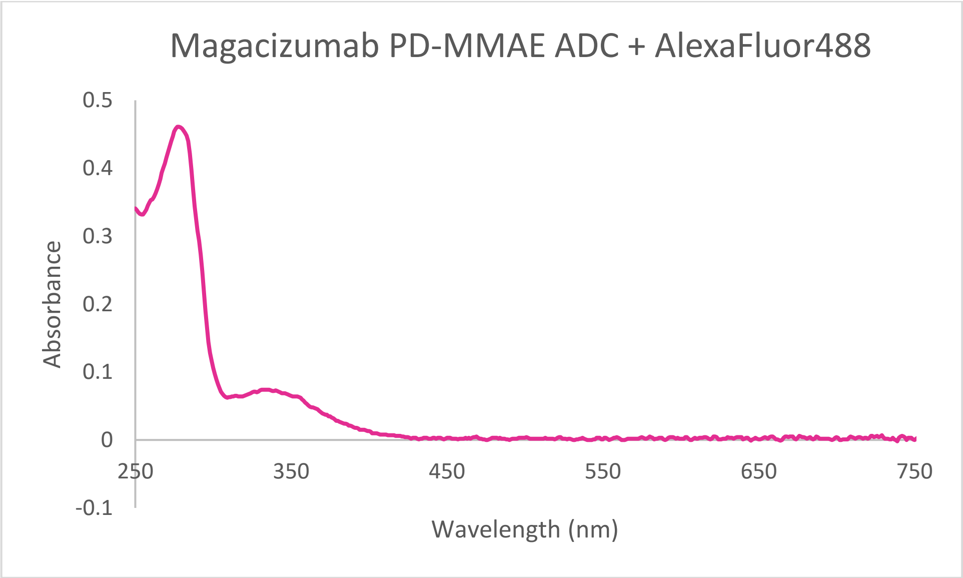

Figure S21 *UV-Vis data of Magacizumab PD-MMAE ADC* ***5*** *clicked with Alexa Fluor* ^{TM} *488*

Cell Biology

Cell culture

B16F0 mouse melanoma cells (ATCC) were maintained in Dulbecco's Modified Eagle's Medium (DMEM) supplemented with glucose (4.5 g/L), sodium pyruvate (110 mg/L), 10% Fetal bovine serum (FBS), penicillin (100,00 U/L) and streptomycin sulphate (100 mg/L). Transfected B16F0 cells were also cultured as described above and additionally supplemented with G418 antibiotic (1.0 mg/mL). All cultures were maintained at 37 °C in 5% CO_2.

Human *Lrg1* expression in mammalian cells

Competent MC1061 cells were transformed with the construct, single colonies were picked, the DNA was purified by miniprep (Qiagen) and the sequence verified by Sanger sequencing. In preparation for mammalian cell transfection, the plasmid (pcDNA3.1-h*Lrg1*, obtained from Xiaomeng Wang) was purified by maxiprep (Qiagen EndoFree Plasmid Maxi Kit).

In brief, B16F0 cells (0.5×10^6 cell/mL, plated 24 h previously) were transfected with 14 μg of plasmid, using Lipofectamine 2000 (Thermofisher) according to the manufacturer's instructions. 24 h after transfection, the plasmid/Lipofectamine solution was removed and the medium was replaced with fresh cell growth medium without added antibiotics. 48 h following transfection, cells were diluted to a density of 0.5×10^6 cell/mL and cultured in fresh cell growth medium containing G418 (1.0 mg/mL, Sigma) to select for transfected cells only. After several selections, transfected cells were isolated.

Quantitative Polymerase Chain Reaction (qPCR)

RNA from transfected and wild type B16F0 cell lines was extracted using the RNeasy mini kit (Qiagen) and analysed for quality using the 4200 TapeStation (Agilent). cDNA was synthesised using the QuantiTect Reverse transcription kit (Qiagen). qPCRs were run on the QuantStudio 6 Flex System (Applied Biosciences) using Power SYBR Green PCR Master Mix (ThermoFisher). All the reactions performed were run in triplicate in a 96-well plate and a housekeeping gene (mouse *Gaphdh*) was used to normalise the results. Normalised expression values were obtained using the formula $2^{-\Delta Ct}$, where C_t represents cycle threshold values. Delta (Δ) C_t values were calculated for each gene subtracting the C_t value of the house keeping gene

from the C_t value of the target gene. Mouse *gapdh* was used as the housekeeping gene. Student's t test was used to calculate significance.

Primer sequences used were as follows: Mouse *Gapdh*: forward, 5'-ACTGAGGACCAGGTTGTCTCC-3'; reverse, 5'- CTGTAGCCGTATTCATTGTCATACC-3'. Mouse *Lrg1*: forward, 5'- CCATGTCAGTGTGCAGATTC -3'; reverse, 5' AAGAGTGAGAGGTGGAAGAG -3'. Human *LRG1*: forward, 5'-CAGCGACCAAAAAGCCCAG -3'; reverse, 5'- ATTTCGGCAGGTGGTTGACA -3'.

Component	Volume per reaction (µL)
Dd water	6.6
Power SYBR	10
Forward primer	1.2
Reverse primer	1.2
Template cDNA	1
Total	15

Table S4 *qPCR components*

Step number	Temperature	Time	No. of cycles
Step 1	95 °C	10 min	1
Step 2	95 °C	15 sec	40
	60 °C	1 min	
Step 3	95 °C	15 sec	1
	60 °C	15 sec	
	95 °C	15 sec	

Table S5 *qPCR program*

Sample preparation for western blotting

Acetone precipitation of hLRG1 from B16F0 cells

Supernatants were collected from transfected and wild type B16F0 cells and to the supernatants were added four volumes of ice-cold acetone. The mixture was incubated at -20 °C for 60 min, followed by spinning at full speed in a centrifuge for 10 min. After this, the supernatant was carefully discarded and excess acetone was allowed to evaporate for 30 min. The pellet was resuspended in 100 µL of 1 × SDS sample buffer ready to be analysed by western blotting.

Western blotting

Protein samples were boiled with sample buffer for 5 minutes at 90 °C. Protein along with 12 µL Protein ladder (Dual colour precision plus protein standards, Biorad) were loaded onto and run on 10% precast polyacrylamide mini-gels (Biorad). The gels were run at 100 V for 90 min and then transferred onto polyvinylidene fluoride (PVDF) membranes (GE Healthcare) by running at 400 mA for 60 min, immersed in transfer buffer (100 mL 10X Tris Glycine + 200 mL methanol + 700 mL water). Membranes were blocked for 60 min at room temperature with TBS-T buffer (Tris-Buffered Saline: 50 mM Tris, 150 mM NaCl, adjusted to pH 7.6, 0.1% Tween-20 (Sigma) and 3% BSA to saturate non-specific binding sites. Primary antibodies were diluted in blocking buffer at concentrations reported in Table S4 at 4 °C with gentle agitation. Membranes were washed 3 × 5 min in TBS-T. Horseradish peroxidase conjugated secondary antibodies were diluted in TBST-3% BSA (according to Table S4) and applied for 1 h at room temperature with gentle agitation. Following incubation, the membranes were washed with TBS-T, treated with ECL (Pierce ECL 2 Western Blotting Substrate) and developed using X-ray films.

**Primary antibody**	**Conjugated**	**Working concentration**	**Source**
Rabbit anti hLRG1		1:1000	Proteintech
**Secondary antibody**	**Conjugated**	**Working concentration**	**Source**
Goat polyclonal anti-rabbit Immunoglobulins	Horseradish peroxidase	1:2000	Dako

Table S6 *Antibodies for western blotting.*

Preparation of Alexa 555-labelled hLRG1

hLRG1 was labelled using the Alexa Fluor 555 protein labelling kit (ThermoFisher), according to the instructions provided by the manufacturer. Briefly, 50 μL of 1 M sodium bicarbonate buffer (pH 8.3) was added to 500 μL (500 μg in PBS) of hLRG1, followed by incubation with the reactive dye in the vial for 1 h at room temperature. Excess dye was removed by applying the reaction mixture to a PD-10 column (Amersham Biosciences). The internalisation of Alexa 555-labeled hLRG1 (10 μg/mL) was then assessed *via* the internalisation assay described below.

In vitro internalization analysis by confocal microscopy

hLRG1-transfected and wild type B16F0 cells on coverslips at 70% confluency were incubated with labelled constructs at 10 μg/mL for 1 h at 4 °C and then at 37 °C. Cells were washed three times with PBS to remove unbound antibodies followed by fixation with 4% formaldehyde for 15 min at room temperature. Coverslips were permeabilised and blocked as described previously. Actin was detected with phalloidin-568 (Invitrogen) and DAPI was used to stain cell nuclei.

As a positive control, cells were incubated with Transferrin-555 at 4 °C and then incubated at 37 °C. Cells were fixed and stained with phalloidin-680 for actin and DAPI for nuclear staining.

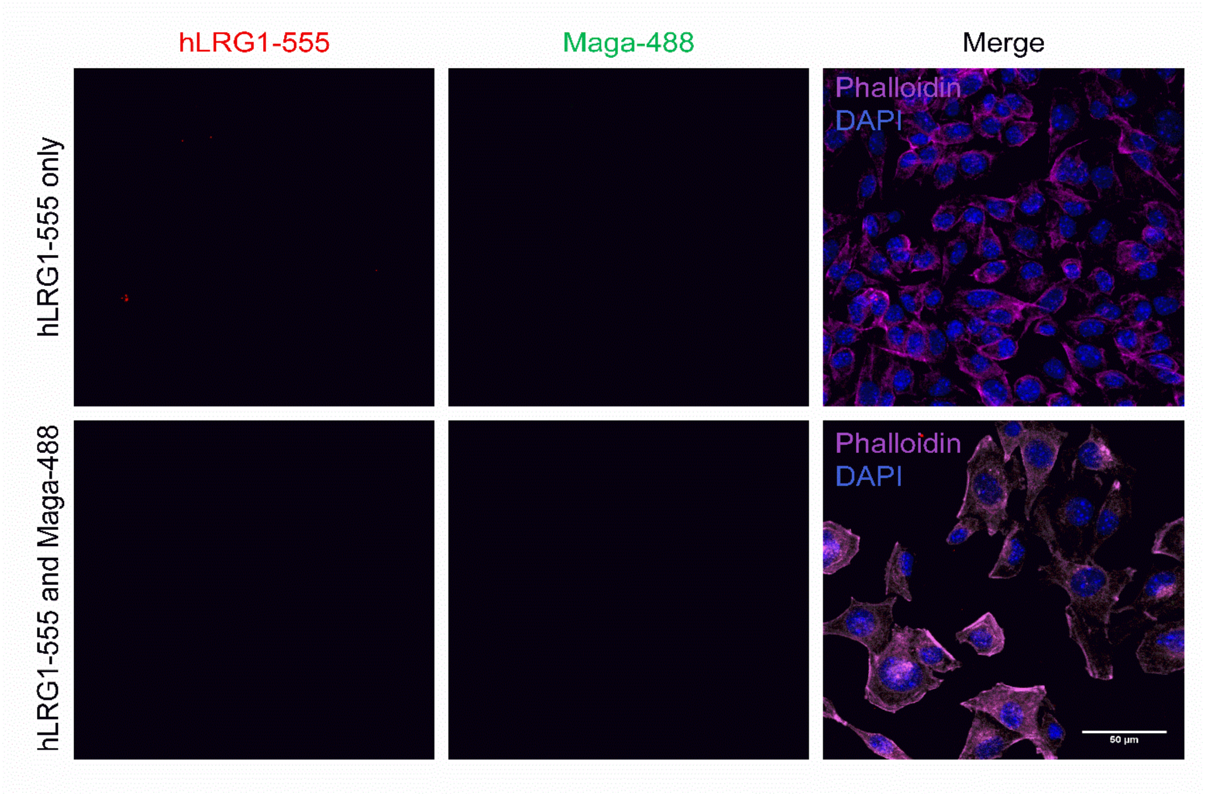

Figure S22 Internalisation analysis of hLRG1-55 by confocal microscopy. *hLRG1-positive B16F0 cells were incubated with hLRG1-555 in the absence and presence of Maga-488 at 37 °C. Phalloidin was used to stain actin and DAPI was used to stain nuclei. Scale bar, 50 µm.*

In vitro cytotoxicity assessment

In vitro cytotoxicity of compounds was evaluated in hLRG1-positive and wild type B16F0 cell lines by the MTT [3-(4,5-dimethylthiazol-2-yl)-2,5-diphenyltetrazolium bromide] colourimetric assay. Briefly, 5×10^4 cells were seeded in 96-well plates and incubated at 37 °C overnight. Cells were then exposed to a range of concentrations of the test compounds diluted in growth medium at pH 6.5 and at 37 °C as follows: MMAE (0-100 nM, 72 h), Cisplatin (0 – 66 µM, 48 h), Magacizumab (0-100 nM, 72 h), N_3-PEG_3-vc-PABC-MMAE (0-100 nM, 72 h) and ADC **5** (0-100 nM, 72h). Following each treatment, cells were washed twice with PBS and the medium was replaced with growth medium free of phenol red. MTT reagent (12 mM) was then added to each well and cells were incubated for 4 h at 37 °C, followed by the addition of DMSO and further incubation at 37 °C for 1 h. Optical density (OD) was measured at 540 nm. The percentage of viable cells was calculated as follows.

$$\text{Cell viability (\%)} = ((OD_{\text{treated cells}} - OD_{\text{blank}})/(OD_{\text{untreated cells}})) \times 100$$

ADC **5** + Cathepsin B[5]: 3.3µL of Cathepsin B (human liver, Sigma-Aldrich, 13.8 µM) was added to 11.7 µL sodium acetate buffer (2.2 M, pH = 5.8), 24 µL 30 mM DTT and 1 µL 500 mM EDTA. The resulting mixture was activated by incubation at room temperature for 15 min, then 360 µL sodium acetate buffered medium (pH 6.5) was added and to this solution was added ADC **5** (100 µL at 10 µM). Cells were treated with this ADC **5** + Cathepsin B mixture as described above for ADC **5** (0-100 nM, 72 h at 37 °C).

ADC **5** + Cisplatin: Cells were initially incubated with 5 µM of Cisplatin for 48 h. Following this, serial dilutions of ADC **5** (0-100 nM) were prepared in growth medium and cells were treated as described for ADC **5** (72 h at 37 °C).

Tumour models

C57BL/6 mice were purchased from Charles River Laboratories. Human LRG1 knock-in mice were generated by the Moss and Greenwood laboratories as described by Kallenberg et al.[3] Single-cell suspensions of 1×10^6 B16F0 cells (human-LRG1 transfected and wild type cells) were injected subcutaneously into the back of *Lrg1*$^{+/+}$ C57BL/6 mice in 100 µL PBS . Mice were randomised by age prior to inoculation. Tumours were measured at defined intervals using calipers and tumour volume was calculated using the formula:

$$V = 4\pi/3\ (1/2\ \text{length} \times 1/2\ \text{width} \times 1/2\ \text{height})$$

Mice were sacrificed at the end of the experiment, or when tumours reached a maximum of 1.5 cm^3 or weight loss exceeded 15% of the total body weight. All procedures were performed in accordance with the UK Animals (Scientific Procedures) Act and the Animal Welfare and the Ethical Review Bodies of the UCL Institute of Ophthalmology.

In vivo tumour localisation analysis of Magacizumab-488 3a

Lrg1$^{+/+}$ C57BL/6 mice were injected subcutaneously into the back with single-cell suspensions of 1×10^6 hLRG1-transfected or wild type B16-F0 cells in 100 µL PBS. Tumours were allowed to grow, and on day 12 post tumour cell inoculation mice were subjected to Magacizumab-AF488 (conjugate **3**) by a single intraperitoneal injection of 100 µg of the conjugate. Mice were

sacrificed 4 h after injection. Tumours were excised and fresh frozen on dry-ice and embedded in optimal-cutting-temperature medium (OCT).

In vivo efficacy assessment

Therapy studies

Lrg1$^{+/+}$ C57BL/6 mice (age, 6–11 wk; weight, 19-31 g) were injected subcutaneously into the back with single-cell suspensions of 1 ×10^6 hLRG1-transfected B16F0 cells in 100 µL PBS. Animals were randomised and allocated to the following groups prior to treatment: (1) Untreated (n=9); (2) Magacizumab (n=9); (3) Cisplatin (n=8); (4) Magacizumab PD-MMAE ADC **5** (n=9); (5) ADC **5** + Cisplatin (n=8). Tumours were measured and therapy was initiated when tumour volumes reached 0.1 cm^3. Magacizumab and ADC **5** were administered at a dose of 20 mg/kg, and cisplatin at 2.5 mg/kg. All treatments were administered by a single intraperitoneal injection every 7 days for 3 weeks. Tumour volumes were measured, and mouse weights were monitored throughout the duration of the study. In addition to weight loss, disease progression was also evaluated qualitatively by observation of behaviour and muscle wasting. Tumour growth curves and survival curves were used to evaluate treatment efficacy.

Immunofluorescence studies

Subcutaneous B16F0 tumour models were fresh frozen on dry ice in Optimal cutting temperature compound (OCT). Contiguous frozen tissue sections were cut at a thickness of 10 µm for therapy studies and 30 µm for localisation studies and stored at -80 °C. Sections were fixed in 4% paraformaldehyde for 15 min at room temperature or 100% methanol for 5 min at -20 °C, depending on antibodies used. After this, sections were washed with PBS and permeabilised with 0.1% Tween in PBS for 10 min. Sections were blocked in 1% BSA prior to overnight incubation with primary antibody at 4 °C. Sections were washed in 0.01% Tween-20 in PBS and incubated with secondary antibodies for 1h at room temperature. Antibodies used to label mouse endothelium were anti-CD31 (Dianova), mouse cathepsin B was labelled using an anti-cathepsin B polyclonal antibody (Invitrogen) and apoptosis was measured using an anti-γ-h2ax antibody (Abcam). Alexa Fluor labelled secondary antibodies were from Thermofisher. Sections were imaged using a using Zeiss 710 confocal microscope.

Maximum intensity projections of z-stacks were analysed using NIS elements software (Nikon).

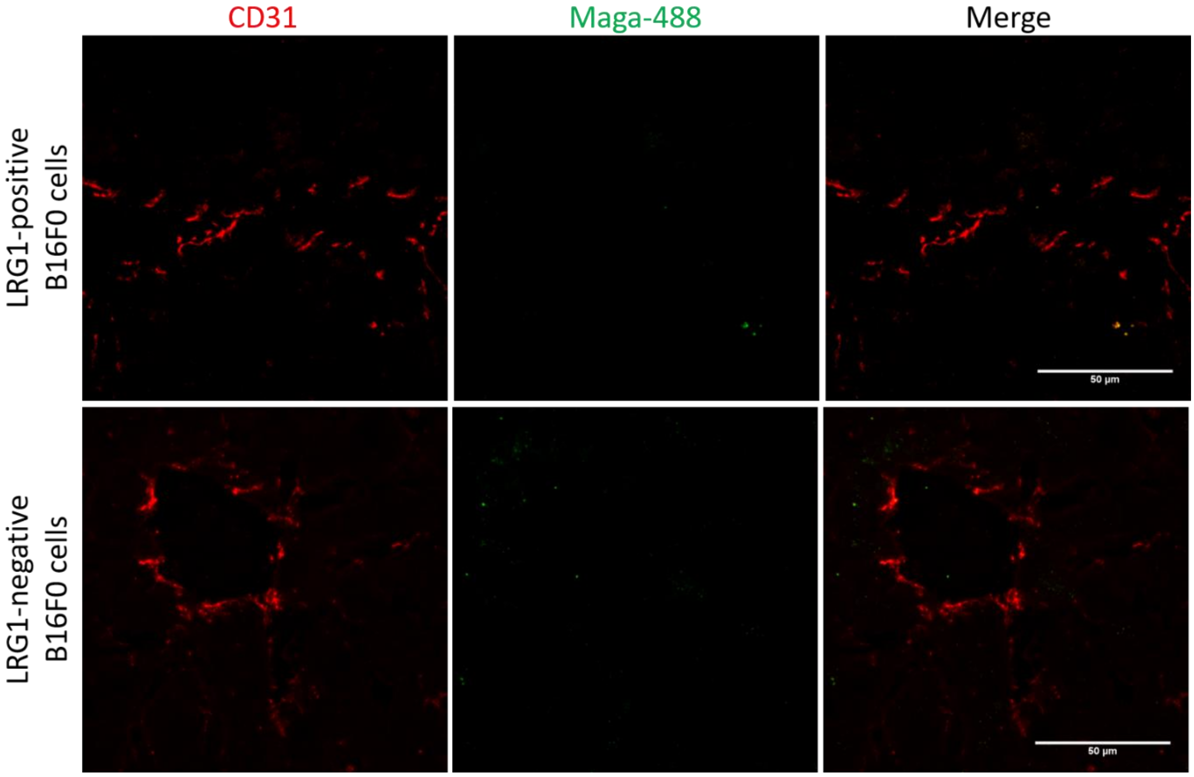

Figure S23 *Magacizumab-488* ***3a*** *does not accumulate in livers of mice expressing hLRG1. Liver sections were collected from mice that received a single injection of Maga-488* ***3*** *at a dose of 100 µg. Livers were excised from mice bearing hLRG1-positive and hLRG1-negative tumours, sectioned and subjected to immunofluorescence staining. Blood vessels were stained using anti-CD31 (red). Scale bars, 50 µm.*

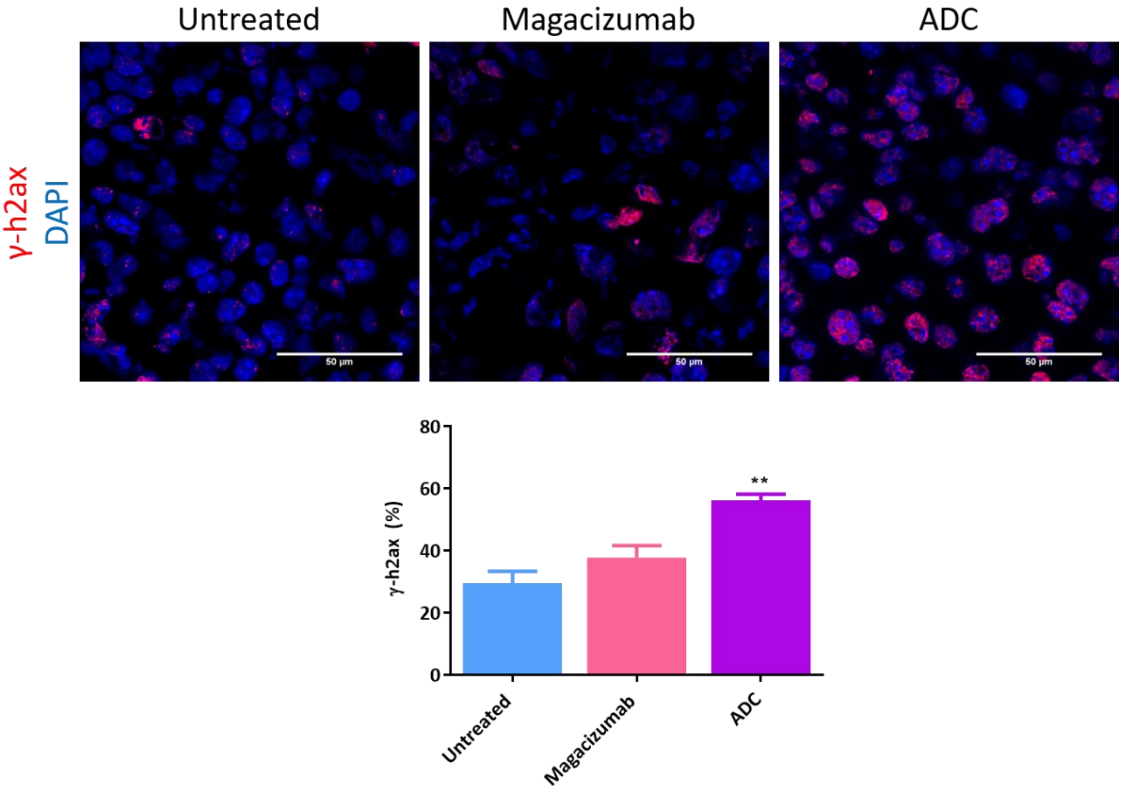

Figure S24 *Analysis of apoptosis using the DNA double-stranded break marker γ-h2ax. DNA double strand breaks detected with antibody against γ-H2AX (red). Scale bar, 50 µm. Data represents percentage of γ-h2ax-positive nuclei, expressed as mean ± SEM (**P < 0.01, One-way ANOVA on ranks with Dunn's multiple comparisons). Untreated, n= 5; Magacizumab, n=5, ADC* ***5****, n= 5.*

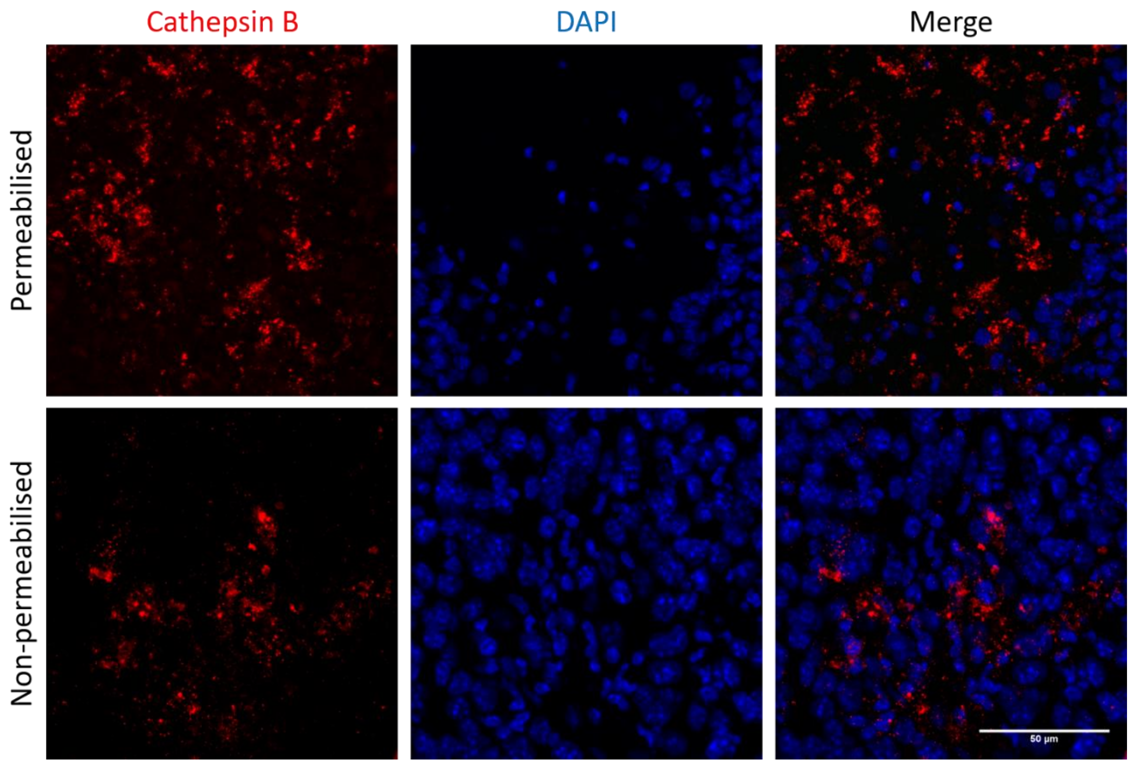

Figure S25 *Cathepsin B is present in the tumour microenvironment of B16F0 melanoma tumour models with or without permeabilisation of tumour sections. Nuclei were stained using DAPI. Scale bar, 50 µm.*

Statistical analysis

Statistical analysis was performed using Graphpad Prism version 6.0 for Windows, Graphpad software (La Jolla California USA, www.graphpad.com). Error bars and statistical tests used for each experiment are indicated in the figure legends. A *P* value of less that 0.05 was considered statistically significant.

1 A. Maruani, H. Savoie, F. Bryden, S. Caddick, R. Boyle and V. Chudasama, *Chem. Commun.*, 2015, **51**, 15304–15307.

2 C. Bahou, D. A. Richards, A. Maruani, E. A. Love, F. Javaid, S. Caddick, J. R. Baker and V. Chudasama, *Org. Biomol. Chem.*, 2018, **16**, 1359–1366.

3 D. Kallenberg, V. Tripathi, F. Javaid, C. Pilotti, J. George, S. Davis, J. W. D. Blackburn, M. O'Connor, L. Dowsett, C. Bowers, S. E. Liyanage, M. Gourlaouen, A. Hoeh, F. Mota, D. Selwood, J. Bainbridge, V. Chudasama, J. Greenwood and S. E. Moss, *bioRxiv*, 2020, 2020.07.25.218149.

4 E. Robinson, J. P. M. Nunes, V. Vassileva, A. Maruani, J. C. F. Nogueira, M. E. B. Smith, R. B. Pedley, S. Caddick, J. R. Baker and V. Chudasama, *RSC Adv.*, 2017, **7**, 9073–9077.

5 R. Gébleux, M. Stringhini, R. Casanova, A. Soltermann and D. Neri, *Int. J. Cancer*, 2017, **140**, 1670–1679.
